# Highly regioselective surface acetylation of cellulose and shaped cellulose constructs in the gas-phase†

**DOI:** 10.1039/d2gc01141g

**Published:** 2022-06-21

**Authors:** Tetyana Koso, Marco Beaumont, Blaise L. Tardy, Daniel Rico del Cerro, Samuel Eyley, Wim Thielemans, Orlando J. Rojas, Ilkka Kilpeläinen, Alistair W. T. King

**Affiliations:** Department of Chemistry, University of Helsinki AI Virtasen aukio 1 00560 Helsinki Finland tetyana.koso@helsinki.fi alistair.king@helsinki.fi; Department of Chemistry, Institute of Chemistry for Renewable Resources, University of Natural Resources and Life Sciences Vienna (BOKU) Tulln Austria; Department of Bioproducts and Biosystems, School of Chemical Engineering, Aalto University Espoo Finland; Sustainable Materials Lab, Department of Chemical Engineering, KU Leuven Campus Kortrijk Etienne Sabbelaan 53 8500 Kortrijk Belgium; Bioproducts Institute, Departments of Chemical and Biological Engineering, Chemistry and Wood Science, University of British Columbia Vancouver BC Canada; VTT Technical Research Centre of Finland Ltd Tietotie 4e 02150 Espoo Finland alistair.king@vtt.fi

## Abstract

Gas-phase acylation is an attractive and sustainable method for modifying the surface properties of cellulosics. However, little is known concerning the regioselectivity of the chemistry, *i.e.*, which cellulose hydroxyls are preferentially acylated and if acylation can be restricted to the surface, preserving crystallinities/morphologies. Consequently, we reexplore simple gas-phase acetylation of modern-day cellulosic building blocks – cellulose nanocrystals, pulps, dry-jet wet spun (regenerated cellulose) fibres and a nanocellulose-based aerogel. Using advanced analytics, we show that the gas-phase acetylation is highly regioselective for the C6-OH, a finding also supported by DFT-based transition-state modelling on a crystalloid surface. This contrasts with acid- and base-catalysed liquid-phase acetylation methods, highlighting that gas-phase chemistry is much more controllable, yet with similar kinetics, to the uncatalyzed liquid-phase reactions. Furthermore, this method preserves both the native (or regenerated) crystalline structure of the cellulose and the supramolecular morphology of even delicate cellulosic constructs (nanocellulose aerogel exhibiting chiral cholesteric liquid crystalline phases). Due to the soft nature of this chemistry and an ability to finely control the kinetics, yielding highly regioselective low degree of substitution products, we are convinced this method will facilitate the rapid adoption of precisely tailored and biodegradable cellulosic materials.

## Abstract 2

Gas-phase acetylation of celluloses is shown to be highly regioselective for 6-OH and confinement of the reaction to the surface.

## Graphical abstract

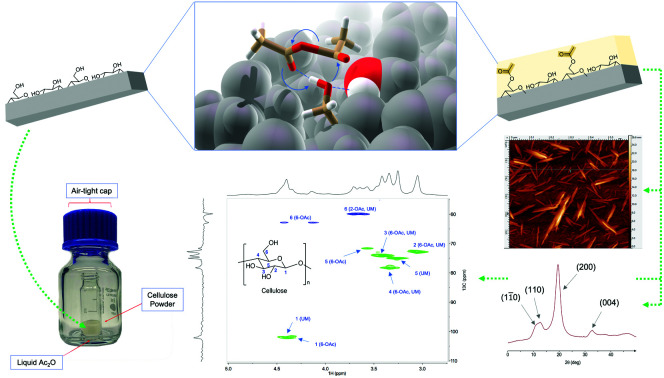

## Introduction

Cellulose has been the most important polymer in the development of humankind. In its technical form (mainly cotton, chemical wood-based pulps and flax fibre, or other plant-based fibres), it has had a multitude of applications spanning thousands of years – from papermaking to production of textiles, to the first thermoplastics and beyond.^1^ The concept of the ‘Cellulose gap’^2^ has highlighted the need to find replacement feedstocks for cotton, to satisfy our increase in population and standards of living. However, with wood-based chemical pulps, as a replacement feedstock, recent trends have shown that existing paper-grade pulp mills are decreasing in profitability, and production is generally below capacity.^3^ Therefore, significant efforts are being put into bulk valorisation of a range of chemical pulps, other cellulosic materials, and their precursor feedstocks. In the short term, there is clear interest in the use of fibrous cellulosic materials, of all length-scales (nano- to macro-fibrillar), in applications such as improved-strength composite materials.^4^ Paper-grade pulp, specifically, is finding rapid adoption in foam-forming.^5^ A longer-term goal is the use of paper-grade pulp^6,7^ and perhaps even lower purity and recycled cellulosics, in regenerated fibre production.^8^

The topic of nanocelluloses also has significant long-term interest – as substrates for materials applications,^9^ mainly based on the high aspect-ratio that nanocellulose provides in the formation of ‘nanopapers’, high-strength materials and their composites.^10,11^ However, there are also more interesting intrinsic properties, such as, nanocelluloses’ axial chirality^12^ and the potential for asymmetric or regioselective-surface chemical modification,^13,14^ that allow for the preparation of novel materials, not easily achieved through other building blocks. For example, these nano-scaled particles have been exploited to form a range of porous materials, where a complex network with various degrees of long-range order is present.^15,16^ Overall, the wide implementation of such fibrous materials, (macro to nano) foams or aerogels, in application, is currently restricted due to the challenges in altering their surface chemistry, whilst preserving their complex architectures.

An obvious dimension to develop the intrinsic qualities of nanocelluloses is controllable surface chemical modification, where the crystallinity and morphology of the native materials should be maintained, but the surface properties are modified in a controllable manner.^11,17^ This not only can be used to introduce functional chemical moieties but also for modification of surface properties, for physiochemical interaction with different media, *e.g.*, in nanocomposites and dispersions.^11,18–20^ Thus, understanding and controlling the surface chemistry of these materials is crucial. The traditional heterogeneous liquid-phase modifications of cellulose are already well studied, especially for those low-cost reactions that are performed industrially.^21,22^ One of the most successful commercial reactions is acetylation, typically performed using inexpensive acetic anhydride (Ac_2_O) and acetic acid (AcOH), with sulfuric acid (H_2_SO_4_) as catalyst.^23^ This method has been shown to yield almost exclusively cellulose triacetate (CTA), where the outer fibril chains are converted to CTA, followed by a progressive conversion of the inner chains towards the core.^24^ In the industrial process, lower degree of substitution (DS) values are only accessible by applying a ‘ripening’ stage, where water is added to the batch reaction to hydrolyse some of the acetate esters. Clearly this method cannot preserve the crystallinity and morphology of the native materials, which are often so critical for the performance of the obtained materials in application. Moreover, from an environmental perspective, ‘chemically modified’ cellulose is defined as a ‘single-use plastic’ under recent EU legislation,^25^ designed to combat plastic pollution of aquatic systems. This is partly prompted by the fact that high DS cellulose acetates, produced through this classical acetylation method, are now shown to have very poor biodegradability, despite being biopolymer in origin.^26^

Due to the amphiphilic nature of the lattice interactions for crystalline cellulose,^27^ exotic amphiphilic solvents are typically required for the complete dissolution of cellulose.^28^ However, if the DS of surface modification is high enough, the surface chains can strip off into common bulk molecular solvents, depending on the solvent and introduced functionality.^29^ This then puts limits on our ability to control surface modification, to only the surface chains using liquid-phase chemistry.

Gas-phase reactions, in contrast, potentially offer lower costs than the liquid-phase reactions, do not require hazardous solvents and do not immediately provide a bulk solvating medium for the exfoliation of cellulose chains. Another important feature is that many delicately shaped cellulose materials, *e.g.* nano-structured aerogels, do not survive immersion in liquids. In addition, due to the low-cost, high volatility and high reactivity of most common acylating reagents, acylations are an important class of reaction for the modification of the properties of cellulosics. Even high boiling aliphatic acylating agents are of use to impart significant hydrophobicity to cellulosic materials at low DS.^30^

The first patent on gas-phase modification of cellulose, using gaseous Ac_2_O, was already published at the beginning of the 20^th^ century.^31^ Similarly, regenerated cellulose fibres (rayon) have been impregnated with aqueous potassium acetate and acetylated up to an acetate content of 35% (bulk DS of 2.4 – well beyond surface confinement), using gaseous Ac_2_O.^32^ Many more modern reports concern both gas- and liquid-phase acylation of cellulose and nanocelluloses, many for the purposes of improving phase compatibility and behaviour in composites or suspensions.^33^ While many patents and publications have been made concerning gas-phase acylation of wood and cellulose, there are limited studies on the regioselectivity of these surface acylations, in part due to the absence of suitable high-resolution analytics. Consequently, our aim in the present manuscript is to better understand the regioselectivity of gas-phase reactions, using classical acetylation as a model reaction. In addition, we will showcase that this gas-phase acetylation can be selective for both native cellulose I and regenerated cellulose II architectures, without disrupting the supramolecular structure of delicate shaped nanocellulose-based objects. The implications for this on the production of biodegradable cellulose-based materials will also be discussed.

## Experimental

Full details for materials and methods are given in the ESI.†

### Gas-phase acetylation

The samples were acetylated in the gas-phase as follows: 100 mg (0.617 mmol) of cellulose was placed into an opened 4 ml vial and sealed in a 100 ml Schott bottle (ESI-Fig. S2†), containing 0.58 ml (0.63 g; 6.17 mmol) of Ac_2_O. The reaction chamber was left to stand at the specified temperature for a fixed time. After cooling, the vial with the cellulosic material was removed. The acetylated cellulose was then washed with EtOH (2–3 × 3.5 ml) and centrifuged, followed by freeze-drying, for analysis. The acetylated CNC aerogel sample was dried only using vacuum at RT, allowing for complete removal of Ac_2_O and AcOH.

### Regioselectivity analytical workflow

A suitable analytical workflow for assessing surface and 6-OH acetylation selectivity (as in Fig. 4) is as follows: acetylated samples are analysed using wide-angle X-ray scattering (WAXS – with the users preferred diffractometer geometry) and by NMR (^1^H and diffusion-edited ^1^H NMR) in the [P_4444_][OAc]:DMSO-d_6_ electrolyte.^34,35^ Changes in DS and 6-OH acetylation regioselectivity can then be determined by suitable baseline correction and peak-fitting the data (ESI-Fig. S3.4 and 3.5†), to give the 6-OH acetylation regioselectivity index (RI). The crystallinity index (CI) can be determined, preferably, using one of the recent diffraction peak and amorphous contribution fitting methods (ESI-Fig. S1.4†) or Rietveld refinement.^36^ In our case, peak-fitting of the NMR and WAXS data was achieved using ‘*Fityk*’.^37^ By combining both the RI and the CI, a good estimate of the overall regioselectivity (including if the reaction is confined to the surface of the elementary fibrils), can be made (Fig. 4).

### Transition-state modelling

The ORCA 4 package^38^ was used with the B86 GGA functional,^39,40^ def2-SVP basis set, Grimme's D3 dispersion correction^41^ with Becke–Johnson dampening^42^ and the resolution-of-identity (RI) approximation.^43,44^ An initial cellulose I_β_ fibril (hexagonal 36 chain), with polymer lengths of 4 glucose units, was generated using ‘Cellulose-Builder’^45^*via* the web interface. This was then edited in Avogadro 1.2^46^ to remove all polymer chains except for a (110) Miller index surface diagonal section of 3 stacked polymer chains with a length of 4 anhydroglucose units (AGU's) each (ESI-Fig. S48†). Hydroxyl groups were added to the reducing ends, as these are missing in the Cellulose-Builder outputs. For the relaxed potential energy surface (rPES) scans, an acetate was added to the relevant OH (2, 3 or 6) of a central AGU. rPES scans for the dihedral angles, corresponding to acetate group rotation (Fig. 3), were completed at the RI-BP86/def2-SVP-D3(BJ) level throughout the full 360°; except in the case of 3-OAc where the calculations failed at certain dihedral ranges, due to steric interactions giving highly distorted geometries (Fig. 3); constraints were used on all atoms except the 6-OAc and relevant adjacent positions on the same molecule surface – all OH oxygens, all 1,2,3,4-hydrogens attached to OHs and the 6-CH_2_ positions attached to 6-OH and 6-OAc (ESI-Fig. S49†). This prevented movement of the AGUs away from the geometry found in the typical cellulose I_β_ crystalline structure but allowed for enough freedom for formation and breakage of H-bonds, necessary for accurate stabilisation of the conformers. Transition state (TS) searches were then performed on the minima provided in Fig. 3, followed by calculation of the analytical frequencies for Gibbs free energy determination. Due to the same constraints applied during the TS search, as were applied for the rPES scans, multiple imaginary frequencies are an unavoidable artifact. The dihedral rPES scans and final TS geometries are shown in Fig. 3 (main text). The Gibbs energies of the TS's, only, were compared against the lowest TS energy (for 6-OH acetylation). Full reaction profiling was completed but not provided, as there is a significant contribution from basis set superposition error (BSSE) using the current basis set (def2-SVP). Rather, the BSSE for the TS's was assumed to be approximately the same, allowing for direct comparison of the TS energies, but not the full reaction profiles. This is not possible with minimized – starting, intermediate, and ending – reaction geometries which have much more relaxed or even fully separated species, leading to much lower basis-set overlap and hence BSSE.

## Results and discussion

### Regioselectivity on model cellulose nanocrystals

**Fig. 1:**
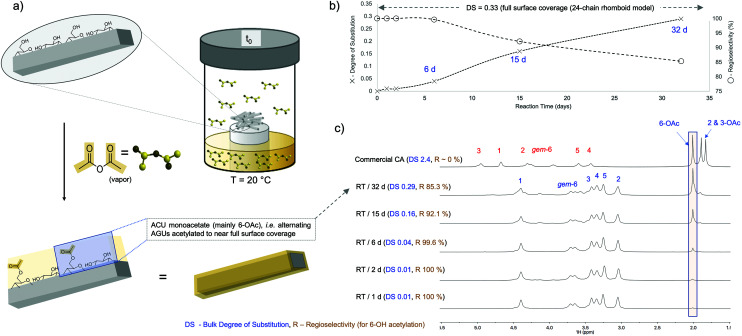
(a) Reaction setup and expected surface regioselectivity, (b) bulk degree of substitution and 6-OH acetylation regioselectivity (RI), (c) diffusion-edited ^1^H NMR spectra showing high RI (decreasing RI as DS increases) and a bulk DS (at 32 days) close to that predicted for full 6-OH surface acetylation of a 24-chain rhomboid softwood elementary fibril model^47^ – CA is cellulose acetate (DS ∼2.4).

We utilized a novel solution-state NMR method for analysis of bulk crystalline celluloses; by combining ^1^H–^13^C HSQC, ^1^H and diffusion-edited ^1^H NMR spectroscopy,^34,35^ it was possible to follow the DS and regioselectivity of acetylation of commercial freeze-dried Southern Pine cellulose nanocrystals (FD-CNCs, H_2_SO_4_-hydrolyzed). Reactions were performed in a simple atmospheric-pressure reaction vessel (a Schott bottle), with FD-CNCs exposed to Ac_2_O vapor (Fig. 1a). Considering that these FD-CNCs were derived from Southern Pine, the Fernandes *et al.* 24-chain rhomboid model for softwood serves as the only published softwood model.^47^ Using this model, full 6-OH surface acetylation corresponds to a bulk DS of 0.33. Other fibrillar models giving full 6-OH surface bulk DS values ranging between 0.22 to 0.33 (ESI-section S2†), with the commonly accepted 18-chain hexagonal model for cell-wall biosynthesis also giving a full surface 6-OH acetylation DS value of 0.33. By gas-phase acetylation of the FD-CNCs over a period of 32 days at room temperature (RT), the combination of ^1^H NMR (including peak-fitting to determine DS, ESI-section S3†) and diffusion-edited ^1^H NMR showed a steady increase to a DS of 0.29 with visibly high RI – regioselectivity for 6-OH acetylation (Fig. 1b and c). This value is also rather close to the experimentally determined bulk DS value of 0.24 for the succinylation of hardwood-based cellulose nanofibrils (CNF), using a novel regioselective aqueous-based method.^48^ As the FD-CNCs were prepared by H_2_SO_4_ hydrolysis, the presence of sulphate half esters is expected. This was confirmed and they were still present after the gas-phase acetylation, as demonstrated in the diffusion-edited ^1^H spectra (ESI-Fig. S28†) and compared against literature assignments.^35^

**Fig. 2:**
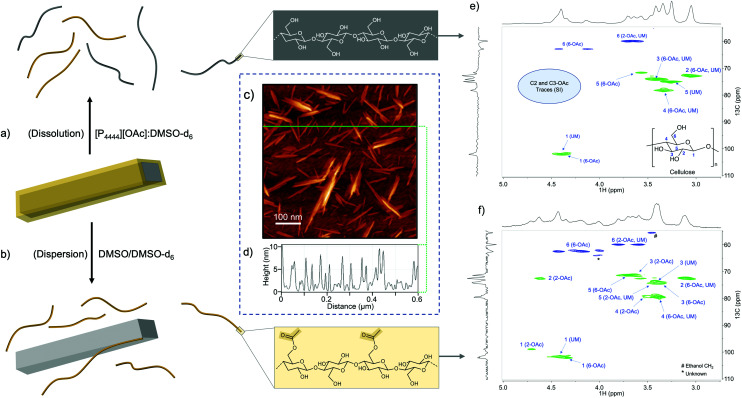
(a and b) Analytical methodology leading to AFM and NMR analyses, (c and d) High resolution AFM for the 32 day acetylated FD-CNCs (with cross-section profiles), and HSQC spectra for: (e) 32 day acetylated FD-CNCs in the [P_4444_][OAc]:DMSO-*d*_6_ electrolyte (bulk analysis), (f) 32 day acetylated FD-CNCs dispersed into DMSO-*d*_6_ (surface chains detectable only).

The 32 day sample was further analysed using 2D ^1^H–^13^C heteronuclear single-quantum correlation (HSQC) spectroscopy. The sample was analysed initially in the [P_4444_][OAc]:DMSO-*d*_6_ electrolyte, to further confirm the bulk regioselectivity of acetylation (Fig. 2a). The sample was also suspended in DMSO-*d*_6_, where only surface chains can be exfoliated into solution (Fig. 2b), further allowing determination of regioselectivity. The nanocrystalline nature for the 32 day acetylated sample was also confirmed to be preserved by high-resolution AFM after casting from DMSO (Fig. 2c and d) – importantly, the acetylation resulted in partial recovery of the elementary nanocrystals (Fig. 2c) from the typically observed pristine clusters (ESI-Fig. S42†). While the cross-sections of the pristine CNCs highlight a clean baseline, with features below 0.3 nm (ESI-Fig. S42†), features larger than 2 nm that are not associated with pristine CNC morphology are observed after acetylation (Fig. 2d). The larger features on the baseline indicate exfoliation of the functionalized cellulose chains with DMSO, subsequently aggregated onto the support substrate. The multiplicity-edited HSQC in the [P_4444_][OAc]:DMSO-*d*_6_ electrolyte confirmed the high regioselectivity for 6-OH acetylation, by the appearance of a new set of *geminal*-CH_2_ 6 (6-OAc) correlations (^1^H – 4.43 & 4.14: ^13^C – 62.89 ppm). Resonances corresponding to 2-CH (2,3-OAc) and 3-CH (2,3-OAc) correlations are apparent, in comparison to literature assignments,^49^ but only after significant scaling (indicating very low relative intensity). When the multiplicity-edited HSQC of the CNCs dispersed in DMSO-*d*_6_ (where CNC surface chains can exfoliate – only solvated chains are ‘visible’ using solution-state NMR) was analysed, an approximate 1 : 1 molar ratio of *geminal*-CH_2_ 6 (6-OAc) and unmodified *geminal*-CH_2_ 6 correlations were apparent, from the 2D correlation peak volumes (ESI-Fig. S41†). This is consistent with surface 6-OH acetylation of the cellulose I_β_ crystalline structure,^27^ where rotation of adjacent AGUs in the crystal lattice results in every alternate polymer AGU 6-OH surface-facing. This further supports the regioselective surface coverage model. As shown in Fig. 1, at bulk DS levels close to the complete C6-OH substitution, the regioselectivity starts to decrease. In addition, a small amount of 2-CH (2-OAc), consistent with the diffusion-edited ^1^H results, is also visible.

### Regioselectivity by DFT transition-state modelling

While it is expected that primary alcohols should react faster, the observed high regioselectivity for gas–solid 6-OH acetylation was pleasantly surprising, and has been so far only recently reported in the case of aqueous-based solid and liquid acylations using *N*-acylimidazoles.^19,48,50^ In comparison to acetylation in the dissolved state,^51,52^ selectivity is clearly improved, although most published bulk DS values for liquid-phase acylation are also typically well above 1 – negating high regioselectivity.

**Fig. 3:**
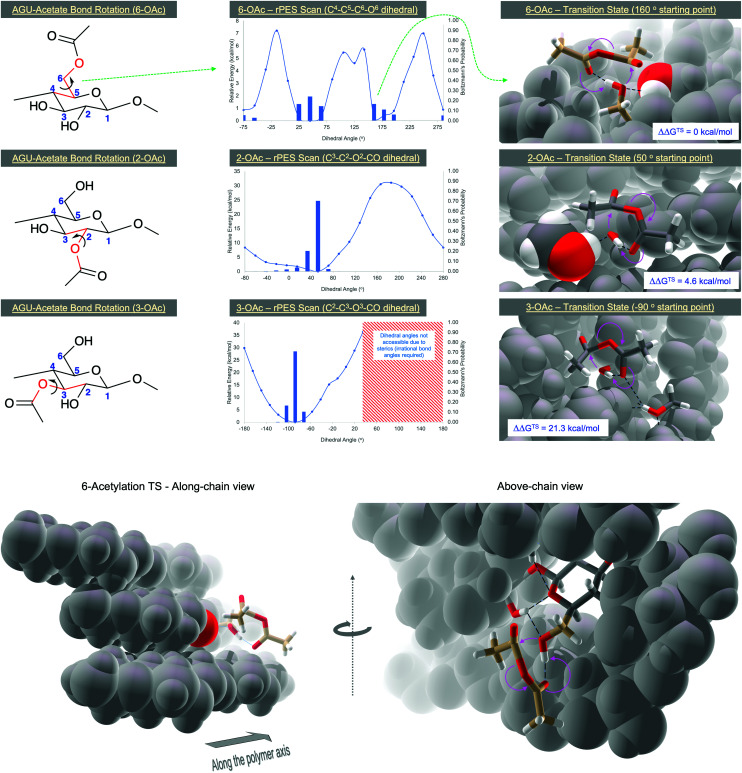
DFT rPES's for acetate-AGU dihedral angle rotation and transition-state calculation of the acetylation mechanism, for 2-, 3- and 6-OH acetylation, using Ac_2_O. H-bonds are in black dotted lines. Movement of electrons on going from OH to OAc form are indicated by magenta arrows.

To further understand why regioselectivity of 6-OH acetylation apparently increases, for gas-phase reactions, DFT transition-state (TS) modelling was performed to locate the TS for acetylation of 2-, 3- and 6-OH groups, and directly compare their Gibbs free energies, relative to the lowest TS (ΔΔ*G*^TS^). All calculations were gas-phase (no implicit solvation) performed at the BP86-D3(BJ)-def2-SVP DFT level of theory, using the ORCA 4 software package.^38^ The use of larger basis sets was not practical due to the system size (274 atoms). ΔΔ*G*^TS^ values were determined by initial calculation of the rPES, for rotation around the dihedral angles where the acetate groups are attached directly to the ring carbon atom (Fig. 3). Due to the additional methylene between the acetate and sugar unit for the 6-OAc, barriers to full rotation were obviously lower and peaked at around 7 kcal mol^−1^ – two main minima were observed. For the 2-OAc, full rotation was presented with an ∼30 kcal mol^−1^ barrier. For the 3-OAc, full rotation was not achievable and the maximum, before failure of the calculation, approached 40 kcal mol^−1^. The minima were used for calculation of the respective TS's. Relative to the 6-OAc TS (ΔΔ*G*^TS^ = 0 kcal mol^−1^), the 2-OAc TS ΔΔ*G*^TS^ = 4.6 kcal mol^−1^ and the 3-OAc TS ΔΔ*G*^TS^ = 21.3 kcal mol^−1^. For comparison purposes – at room temperature, the Boltzmann population of an energy level of 4.6 kcal mol^−1^, relative to 0 kcal mol^−1^, is 0.00042. There are some potential sources of error for this calculation, including basis set superposition error (BSSE), afforded using such a small basis set, or a lack of dynamics, or degrees of freedom due to applied atom constraints. However, such large differences in energy clearly indicate that 6-acetylation in the gas-phase should be strongly favoured. In all cases, intra-unit H-bonding plays an important part in stabilization of each TS, as do steric interactions with adjacent atoms and chains (reduced conformational freedom) in increasing ΔΔ*G*^TS^ values for the 2- and 3-OH acetylations.

### Gas- *vs*. liquid-phase acetylation regioselectivities

**Fig. 4:**
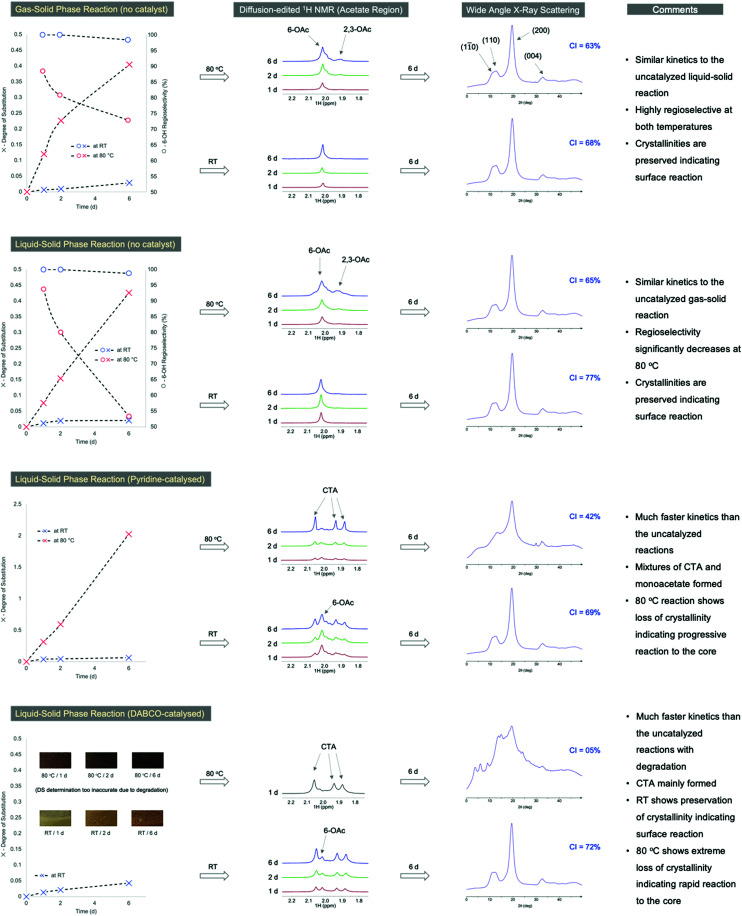
Comparison of regioselectivity (surface reaction confinement and RI) and cellulose I_β_ CI changes, through NMR and WAXS analysis, for gas- *vs*. liquid-phase acetylations (in presence or absence of common neutral organic bases).

To effectively compare whether gas-phase acetylation truly is more regioselective than the alternative liquid-phase methods, an analytical workflow was set up whereby the FD-CNCs were acetylated under different conditions: in gas or liquid Ac_2_O, catalysed or uncatalyzed, at two temperatures (RT & 80 °C) with 3 kinetic time points (1, 2 & 6 days) (Fig. 4). The bulk DS of acetylation and RI's were determined for each by ^1^H NMR measurement and peak fitting (ESI-section S3.4†). Regioselectivity is also visualized using diffusion-edited ^1^H NMR (Fig. 4). The crystallinities also were visualized using the WAXS diffractograms, alongside calculation of the CI values, for the 6 day samples (Fig. 4). Full DS values and crystallinity details are in the ESI (ESI-Tables S2 and 3†). As acid-catalysed methods of liquid-phase acetylation are already well studied, and known to lead to CTA, we focused on non-catalysed (pure Ac_2_O), pyridine catalysed and 1,4-diazabicyclo[2.2.2]octane (DABCO)-catalysed liquid-phase reactions, for comparison with the gas-phase reaction. Regioselectivity for both the gas and liquid Ac_2_O non-catalysed reactions are mostly excellent at lower DS values. Crystallinity is preserved and the acetylation kinetics are fortunately very similar for the uncatalyzed liquid and gas-phase reactions (up to DS ∼0.4 over 6 days at 80 °C). However, the RI for the liquid–solid reaction clearly suffers most at the higher temperature, compared to the gas–solid reaction, dropping down to 53% at 6 days (73% for the gas–solid reaction). When pyridine is used as co-solvent/catalyst, the RI decreases further, with CTA motifs becoming apparent, from the acetate region in the diffusion-edited ^1^H NMR spectra (Fig. 4). This indicates enhanced chain exfoliation and conversion to CTA in the basic liquid-phase environment. Despite this, the crystallinities are largely preserved at RT, indicating confinement of the reaction to the surface. However, at 80 °C the CTA motifs are more prominent, and crystallinity clearly decreases indicating a progression of the reaction towards the CNC cores. In the case of the DABCO-catalysed reaction, the samples colorize at RT and quickly blacken at 80 °C (pyridine- and non-catalysed samples did not colorize), where CTA formation is predominant. Yet, at RT the crystallinity is still largely preserved indicating surface confinement of the reaction.

These comparisons are a clear indication that the gas-phase protocol has much better control over surface and 6-OH acetylation regioselectivities, especially at higher temperature, compared to the liquid-phase methods (minimal surface exfoliation in the gas-phase). This, combined with the low chemical demand, ease of product recovery and benign nature of gas-phase reactions, make it highly suitable for industrial implementation. Moreover, with the base-catalysed liquidphase reactions, the formation of CTA seems to be unavoidable, indicating rapid chain exfoliation and conversion to CTA in the liquid-phase, as with the acid-catalysed process.

When it comes to the reactivity of surface hydroxyls for different elementary fibril cross-sectional models (ESI-section S2†), the bulk kinetics are not expected to change considerably. The literature is not totally clear yet on what is the accepted cross-sectional model. There may also be thermodynamically more stable forms which are accessible using different processing conditions,^53^ that lead to different models and available hydrophobic *vs*. hydroxylated surfaces. However, this may be more relevant towards aggregation of the materials under different processing conditions, which in turn affects the porosity and accessibility of different surfaces to reagents. This is a continuing challenge to fathom.

### Substrate scope & future directions

**Fig. 5:**
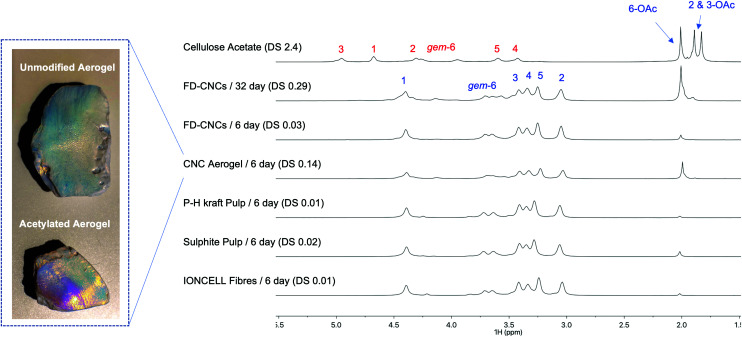
Range of cellulose substrates evaluated where the overall 6-OAc regioselectivity and comparative kinetics are shown through the diffusion-edited ^1^H NMR spectra.

Finally, we decided to assess the regioselectivity, and provide broader insight on acetylation kinetics, for a series of different cellulose substrates (Fig. 5): (1) FD-CNCs, (2) spray-dried CNCs (SD-CNCs), (3) CNC Aerogel, (4) IONCELL® fibres (regenerated cellulose II air-gap spun fibres, using an ionic liquid direct-dissolution solvent),^54^ (5) pre-hydrolysis hardwood (mainly birch) kraft pulp (P–H kraft Pulp, 6.8% hemicellulose), and (6) beech sulphite pulp (3.8% hemicellulose). All samples were analysed after reaction at RT for 6 days. DS and crystallinity data are given in the supporting info (ESI-Table S2†).

Not surprisingly, the SD-CNCs showed very low reactivity (by ATR-IR, ESI-Fig. S44†), compared to the FD-CNCs. It is expected that spray-drying results in much reduced surface area, through aggregation, compared to freeze-drying. XPS analysis (ESI-Fig. S45†) of the samples also showed the presence of silicone (potentially silicates) which may occupy reactive sites and facilitate aggregation during spray-drying. Similarly, the CNC aerogel sample is expected to maintain a high surface area. Surprisingly, this resulted in a higher DS and high 6-OAc regioselectivity, compared to the FD-CNCs. Naturally, the P–H kraft and sulphite pulps should have rather low surface areas, due to their much more complex morphology and hornification during the drying procedures. This is reflected in their lower DS values, which however do seem to maintain high 6-OAc regioselectivity. Finally, the IONCELL® fibres also gave similar DS values to the pulp samples, indicating a large degree of aggregation and low surface area after drying, preventing further reaction under ambient conditions. In the case of the patent concerning gas-phase acetylation of regenerated cellulose fibres,^32^ much higher degrees of acetylation were possible (DS values up to 2.4 – clearly indicating non-selective CTA formation), but only after pre-swelling with potassium acetate solution. Thus, in the application of gas-phase chemistry, specific surface area and porosity of dried materials are of clear importance for reaching high bulk DS values, yet, still maintaining very high regioselectivities. The low reactivity of the fibrillar cellulose samples will likely also be alleviated to some extent using higher temperatures, catalysts, or application of pre-swelling/fibrillating conditions. Initial indications in this study suggest that the 6-OAc regioselectivity may decrease with increasing temperature. However, this can be minimized by targeting lower DS values, as full crystallite surface coverage is likely not required for all applications. This may also be critical in some cases to prevent exfoliation of chains, under subsequent solvating conditions, or to maintain the biodegradability of the native-unmodified polymer/material.

### Biodegradation and legislation

In this context, there are clearly further important future implications of this research, in relation to the recent European Union Single-Use Plastics Directive 2019/904^25^ and other imminent international legislation on the topic – particularly concerning the reduction of the impact of non-degradable and oxo-degradable plastics on the environment, and on aquatic systems, especially. Under the definitions of this directive, it would seem that biopolymers (*e.g.* cellulose), that are not chemically modified, do not come under the definition of ‘single use plastics’, despite the obvious fact that non-modified cellulose is not ‘plasticizable’, without chemical modification, and chemistry is typically involved in their isolation, to technical purity. Nevertheless, controlled chemical surface modification of celluloses to low bulk DS, yet with large effect on the surface properties, seems likely to come under the definition of a ‘single-use plastic’. This is a weakness of the directives’ current definitions and further review of the directive or international legislation will likely depend on future studies into the biodegradability of modified materials, combined with regioselectivity information and including full life cycle analyses for each ‘plastic’ product. A relevant example of this is the use of fibrous cellulose acetate tow in cigarette filters. A recent review^26^ has highlighted this issue, including the broader issue of polymer biodegradation. It was shown that biodegradation of cellulose acetate is very much dependent on DS, with high DS cellulose acetate cigarette filters as one of the main types of disposable single-use plastic not initially covered by the directive. This implies that a controlled bulk modification may still yield highly biodegradable materials, provided the DS is low. This is also consistent with the numerous reports of water solubility of cellulose acetate at low DS^55–58^ – as surface chain exfoliation into water, significant swelling of the polymer or even dissolution are undoubtedly preliminary steps in their rapid biodegradation. Therefore, it must be emphasized that careful control over surface regioselectivity will be critical in the future for wide application of these materials, with cellulose acetylation only one such example of chemical modification.

## Conclusions

In conclusion, we have shown that gas-phase acetylation with acetic anhydride presents a unique opportunity for 6-OH selective modification of a wide range of cellulose I and II substrates. The regioselectivity was more controllable than the corresponding non-, acid- or base-catalysed liquid-phase systems, especially at higher temperatures, where kinetics are more amenable for continuous production. This is highly beneficial for controlled surface modification strategies but also for situations where supramolecular 3D morphology should be retained, as with many delicate shaped cellulose objects, *e.g.*, structurally coloured materials and aerogels. Moreover, fine control over DS and regioselectivity will be critical in the future to co-maintain physiochemical properties and biodegradability. It is intended that this publication will contribute the chemical and analytical know-how required to quickly expand the scope, and the scale, of application for a wide range of sustainable cellulosic materials, including those with complex 3D networks and morphologies. While the current uncatalyzed reaction kinetics are rather slow, the use of different catalysts for gas-phase acetylation will be important to study. Moreover, we anticipate that the analytical methodology presented within will become critical for wider adoption of single-use (biodegradable) cellulosic products, as legislation develops to mitigate environmental damage.

## Author contributions

The experimental work was mostly completed by Tetyana Koso. Supporting analytics (NMR, AFM, WAXS, XPS), were completed by Tetyana, Marco Beaumont, Blaise Tardy, Daniel Rico del Cerro, Samuel Eyley and Alistair King. Tetyana, Marco, Blaise, Wim Thielemans and Alistair did the conceptualisation, with all contributing to editing the manuscript. Alistair performed the computations. All authors contributed to final editing.

## Conflicts of interest

There are no conflicts to declare.

## Supplementary Material

GC-024-D2GC01141G-s001
