# Supplementary material for: Highly regioselective surface acetylation of cellulose and shaped cellulose constructs in the gas-phase
Source: Green Chem. 2022 Jun 21;24(14):5604–13. doi: 10.1039/d2gc01141g (PMC9290444; doi:10.1039/d2gc01141g)
Supplement: GC-024-D2GC01141G-s001 [file GC-024-D2GC01141G-s001.pdf]

Electronic Supplementary Material (ESI) for Green Chemistry.
This journal is © The Royal Society of Chemistry 2022

Highly regioselective surface acetylation of cellulose and shaped cellulose constructs in the gas-phase

Tetyana Koso[1], Marco Beaumont[2,3], Blaise L. Tardy[3], Daniel Rico del Cerro[1], Samuel Eyley[4], Wim Thielemans[4], Orlando J. Rojas[3,5], Ilkka Kilpeläinen[1], Alistair W. T. King[1,6*]*

[1] Department of Chemistry, University of Helsinki, AI Virtasen aukio 1, 00560, Helsinki

[2] Department of Chemistry, Institute of Chemistry for Renewable Resources, University of Natural Resources and Life Sciences Vienna (BOKU), Tulln, Austria

[3] Department of Bioproducts and Biosystems, School of Chemical Engineering, Aalto University, Aalto, Finland

[4] Department of Chemical Engineering, KU Leuven, Campus Kortrijk, Etienne Sabbelaan 53, 8500 Kortrijk, Belgium

[5] Bioproducts Institute, Departments of Chemical and Biological Engineering, Chemistry and Wood Science, University of British Columbia, Vancouver, BC, Canada

[6] VTT The Technical Research Centre of Finland Ltd, Tietotie 4e, 02150 Espoo, Finland

Table of contents

1. Materials and methods ... 3

1.1 Preparation of the $[P_{4444}][OAc]$:DMSO-d_6 Electrolyte for NMR Analysis ... 3

1.2. Typical gas-solid phase acetylation of the cellulosic material ... 4

1.3. Typical liquid-solid phase acetylation of the FD-CNCs ... 5

1.4. Crystallinity index and periodic plane size determination ... 5

2. Crystallite Models ... 17

3. NMR supplementary data ... 17

3.1. NMR sample preparation ... 17

3.2. Diffusion-edited ^{1}H experiments ... 18

3.3. Multiplicity-Edited HSQC Experiments ... 18

3.4. DS determination from ^{1}H NMR spectral data ... 18

3.5. Regioselectivity determination from ^{1}H NMR spectral data ... 20

3.6. The NMR data ... 23

4. AFM ... 31

5. ATR-IR ... 33

6. XPS ... 34

7. Computational Experimental ... 36

8. References ... 65

1. Materials and methods

Different celluloses were used in our reactivity studies; FD-CNCs (freeze-dried softwood CNCs, derived from sulphuric acid digestion of southern pine dissolving pulp, FPL/UMaine PDC), Enocell bleached hardwood pre-hydrolysis kraft pulp (P-H Kraft pulp, 6.8 % hemicellulose), beech bleached sulphite pulp (Sulphite pulp, 3.8 % hemicellulose), CNC aerogel as well as regenerated fibres air-gap spun from [DBNH][OAc] (Sixta et al. 2015)) using the IONCELL technology (IONCELL Fibres). Tetrabutylphosphonium acetate ($[P_{4444}][OAc]$) was prepared to high purity, according to the literature procedures (King et al. 2018; Koso *et al.*, 2020). All the reagents and solvents were high purity (≥98%) and were used as obtained from the commercial suppliers, without further purification. Acetic anhydride (AA) 98% was used as acetylating agent. Products were characterized by Attenuated Total Reflection Infra-Red spectroscopy (ATR-IR) and liquid-state NMR spectroscopy on a Bruker NEO Avance (600 MHz ^{1}H-frequency) spectrometer. Wide-angle X-ray scattering (WAXS) measurements were performed on a PANalytical X'Pert Pro MPD system, with Bragg-Brentano (reflectance) geometry. The diffracted intensity of Cu(Kα) radiation (λ = 1.54 Å, under a condition of 45 kV and 40 mA) was measured in a 2θ range between 5° and 50°; The samples for WAXS were prepared by pressing 50 mg of sample in a KBr-IR press, before calibrating on a glass slide. FE-SEM/STEM (Hitachi S4800) was used for crystallinity characterization of selected samples.

1.1 Preparation of the $[P_{4444}][OAc]$:DMSO-d_6 Electrolyte for NMR Analysis

Tri-*n*-butylphosphine (35 ml, 28.7 g, 142 mmol) and *n*-butyl chloride (30 ml, 26.7 g, 288 mmol) were added sequentially and in one portion to a Teflon-lined 125 ml Parr acid digestion vessel. The vessel was sealed and its contents reacted at 120 °C for 24 h under magnetic stirring. Note: a sealed vessel is necessary as trialkylphosphines of the like rapidly oxidize in the presence of air. Moreover, tributylphosphine is pyrophoric in air. After letting the vessel cool to room temperature, the crude and still mostly liquid product mixture was transferred to a round-bottomed flask (during this stage, rapid crystallization may occur). Excess *n*-butyl chloride (bp 78 °C) was evaporated off using a rotary evaporator. Finally, the product was dried using a high-vacuum rotary evaporator at 80 °C for 5 h, yielding a white crystalline mass (40.3 g, 137 mmol, 98% of theory); mp = 60-65 °C (from the melt) ^{1}H NMR (600 MHz, DMSO-d_6) δ 2.24 – 2.16 (m, 8H), 1.51 – 1.36 (m, 16H), 0.92 (t, *J* = 7.2 Hz, 12H). Dry $[P_{4444}]Cl$ (5.00 g, 16.96 mmol) and potassium acetate (1.67 g, 17.0 mmol) were added to isopropyl alcohol (50 ml, HPLC grade). These were mixed and refluxed with stirring for 20 h. After letting the mixture cool to room temperature and then cooling at -20 °C for 18 h, precipitated potassium chloride was filtered off over Celite 545 and the filtrate evaporated in a rotary evaporator. Chloroform (50 ml) was added and the mixture was again cooled to -20 °C for 18 h, to precipitate further salts, followed by filtration through Celite 545. Finally, the solvent was evaporated and the product dried in a high vacuum rotary evaporator at 90 °C for 6 h to give a pale-yellow crystalline mass (5.20 g, 16.32 mmol, 96% of theory); mp = 46 °C (from the melt); ^{1}H NMR (600 MHz, DMSO-d_6) δ 2.27 – 2.17 (m, 8H), 1.62 (s, 3H), 1.51 – 1.36 (m, 16H), 0.91 (t, *J*= 7.2 Hz, 12H). The electrolyte was prepared by weighing dry $[P_{4444}][OAc]$ into DMSO-d_6 in a 1:4 w/w proportion. This was stored in a sealed vessel to avoid water uptake. The sample was analyzed by NMR to assess purity (Fig. S1).

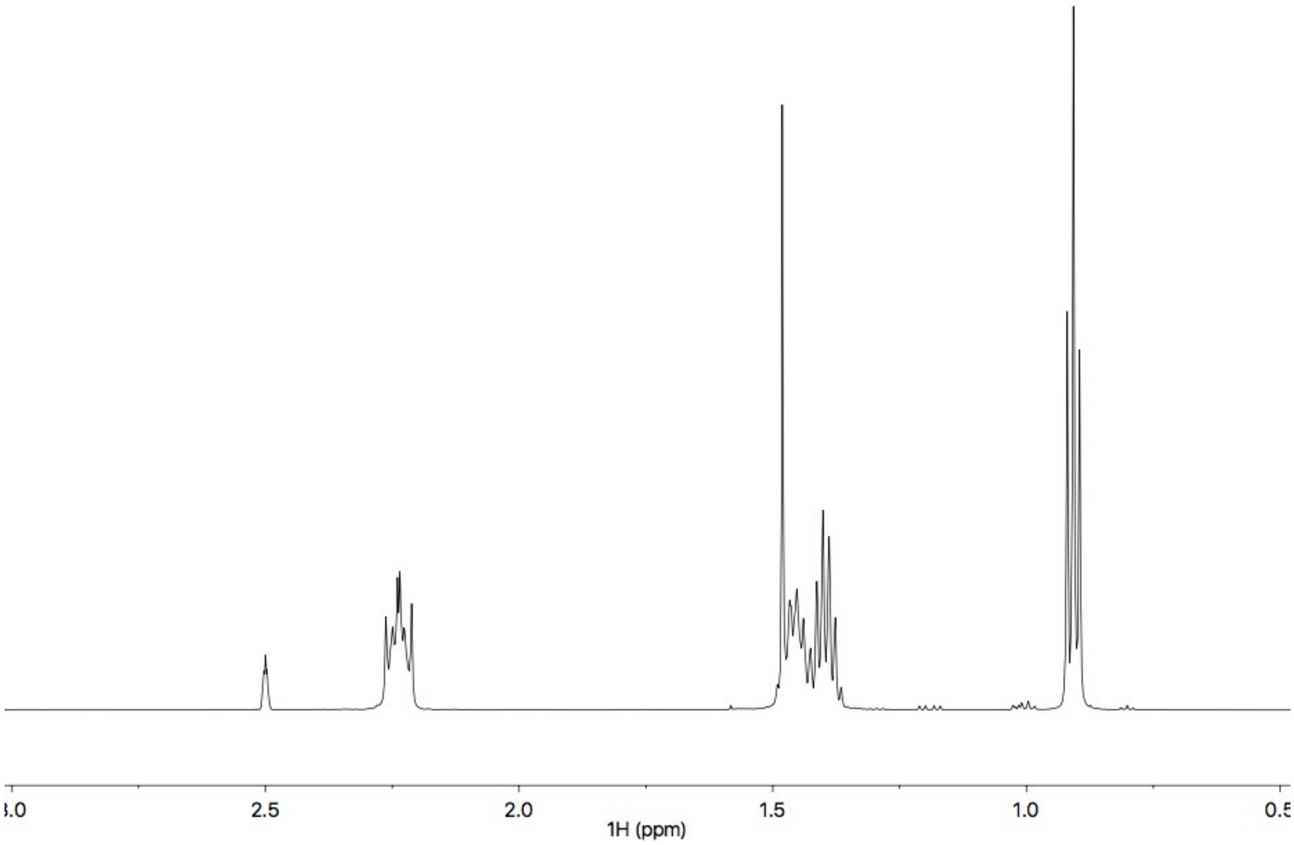

Fig. S1 ^{1}H NMR of the prepared $[P_{4444}][OAc]$:DMSO-d_6 electrolyte

1.2. Typical gas-solid phase acetylation of the cellulosic material

100 mg (0.617 mmol) of cellulose was placed into an opened 4 ml vial and sealed in a 100 ml Schott bottle (**Figure S2**), containing 0.58 ml (0.63 g; 6.17 mmol) of acetic anhydride. The reaction chamber was left to stand at the specified temperature for a fixed time. After cooling, the vial with the cellulosic material was removed. The acetylated cellulose was then washed with EtOH (2-3 $\times$ 3.5 ml) and centrifuged, followed by freeze-drying, for analysis. The acetylated CNC aerogel sample was dried only using vacuum at RT, allowing for complete removal of acetic anhydride or acetic acid.

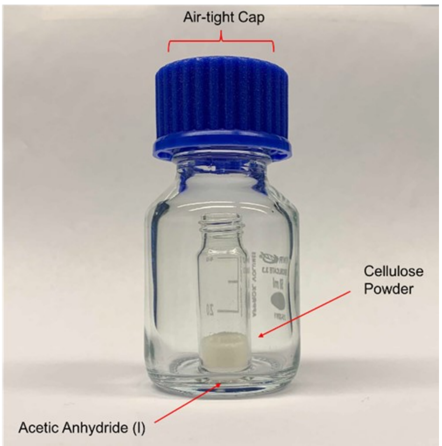

Fig. S2 Typical reaction vessel

1.3. Typical liquid-solid phase acetylation of the FD-CNCs

100 mg (0.617 mmol) of FD-CNCs were placed in the vial and 0.58 ml (0.63 g; 6.17 mmol) of acetic anhydride was added. If required, catalyst (1.85 mmol, 3 eq. to the amount of AGU) was then added. The vessel was sealed and left to stand at ambient temperature (unless stated otherwise) for stated amount of time. The vial contents then washed with EtOH (4-6 x 3.5 ml), centrifuged and freeze-dried for analysis.

1.4. Crystallinity index and periodic plane size determination

The crystallinity index (CI) was determined as described in the previous article supporting information (del Cerro et al. 2020). WAXS diffractograms were fitted with contributions representing background (glass support), amorphous component and main crystalline diffraction plane peaks. "*Fityk*" 1.3.1 peak-fitting software (Wojdyr 2010) was used to process the data through semi-automatic fitting; fitting of functions corresponding to the glass and amorphous backgrounds, as well as set of pseudoVoigt functions.

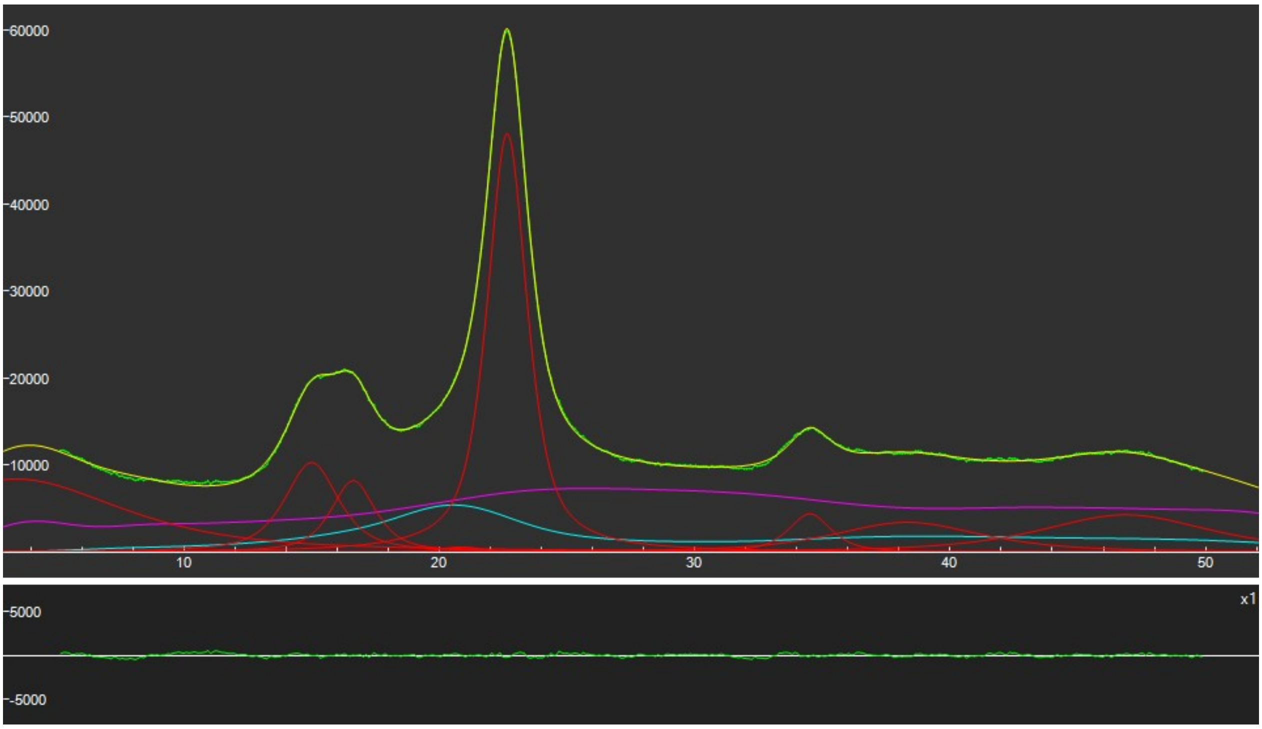

Fig. S3 Fitting of the WAXS diffractogram for the commercial FD-CNC (freeze-dried cellulose nanocrystals) sample in Fityk. Data & functions: raw data (green), fitted data (yellow), amorphous function (cyan), background (magenta), crystalline functions (red) and residual baseline error (green at the bottom of the figure).

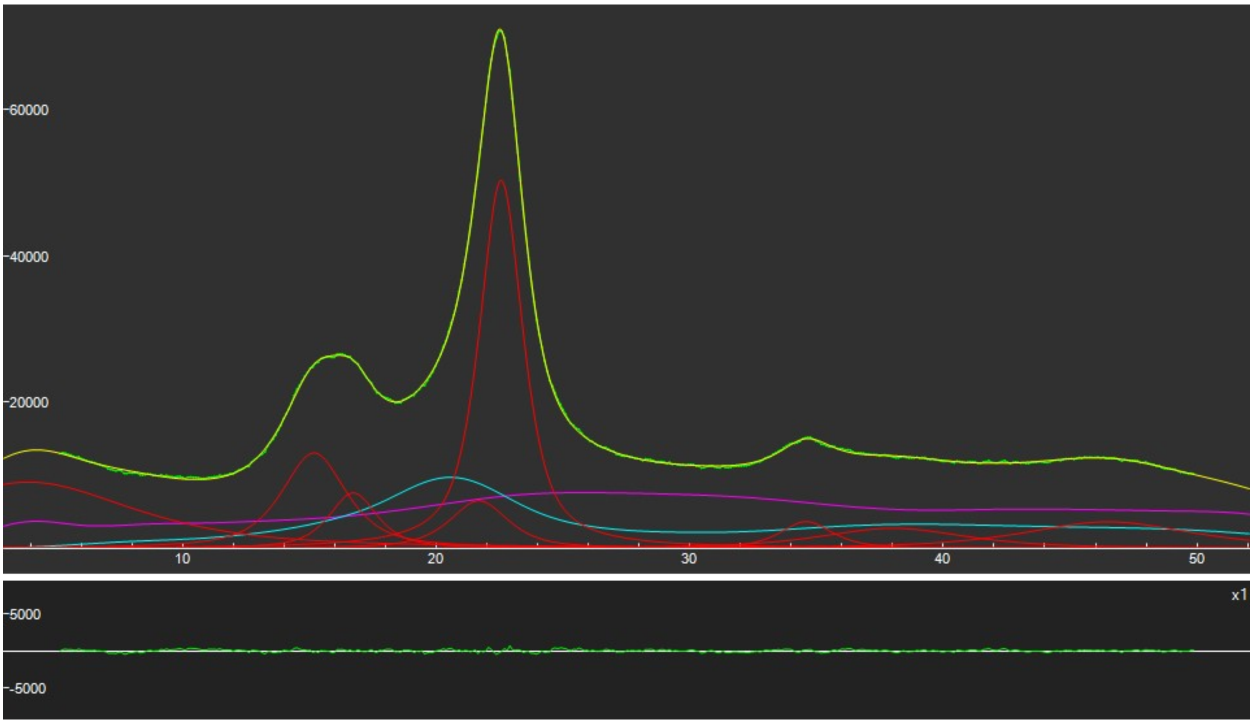

Fig. S4 Fitting of the WAXS diffractogram for the beech BH-S-P (bleached hardwood sulphite pulp) sample in Fityk. Data & functions: raw data (green), fitted data (yellow), amorphous function (cyan), background (magenta), crystalline functions (red) and residual baseline error (green at the bottom of the figure).

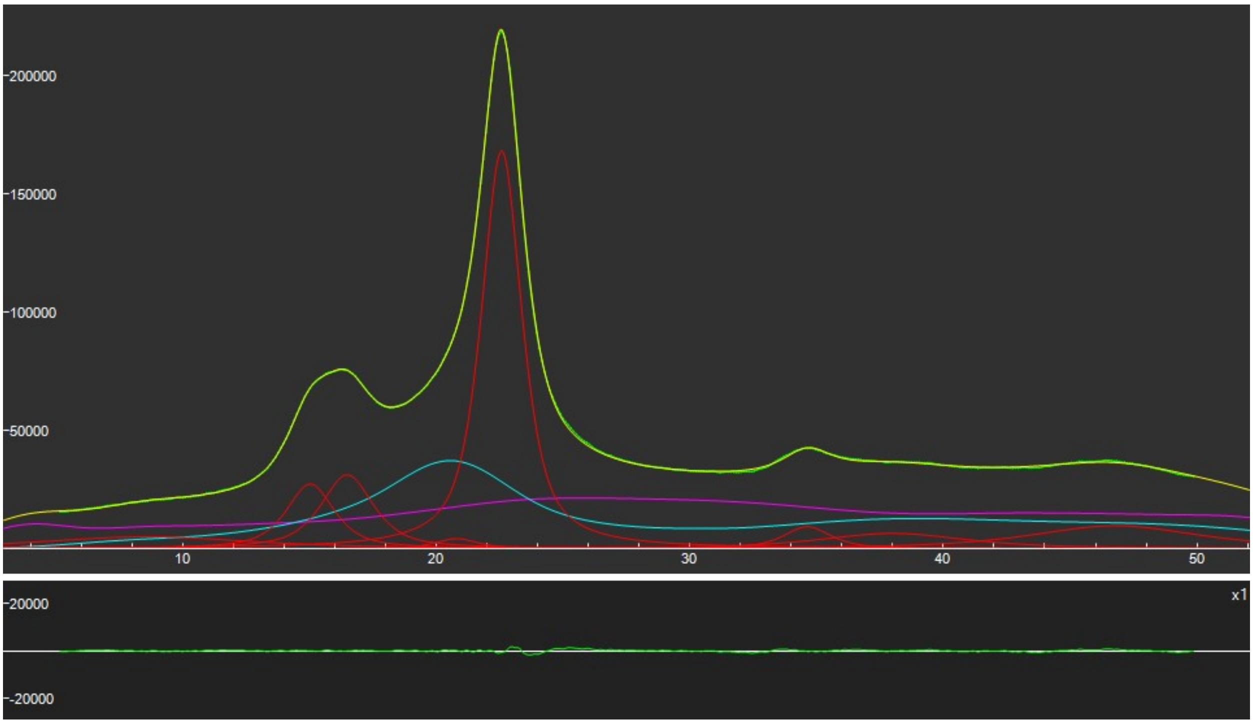

Fig. S5 Fitting of the WAXS diffractogram for the Enocell P-H Kraft (bleached hardwood pre-hydrolysis kraft pulp) sample in Fityk. Data & functions: raw data (green), fitted data (yellow), amorphous function (cyan), background (magenta), crystalline functions (red) and residual baseline error (green at the bottom of the figure).

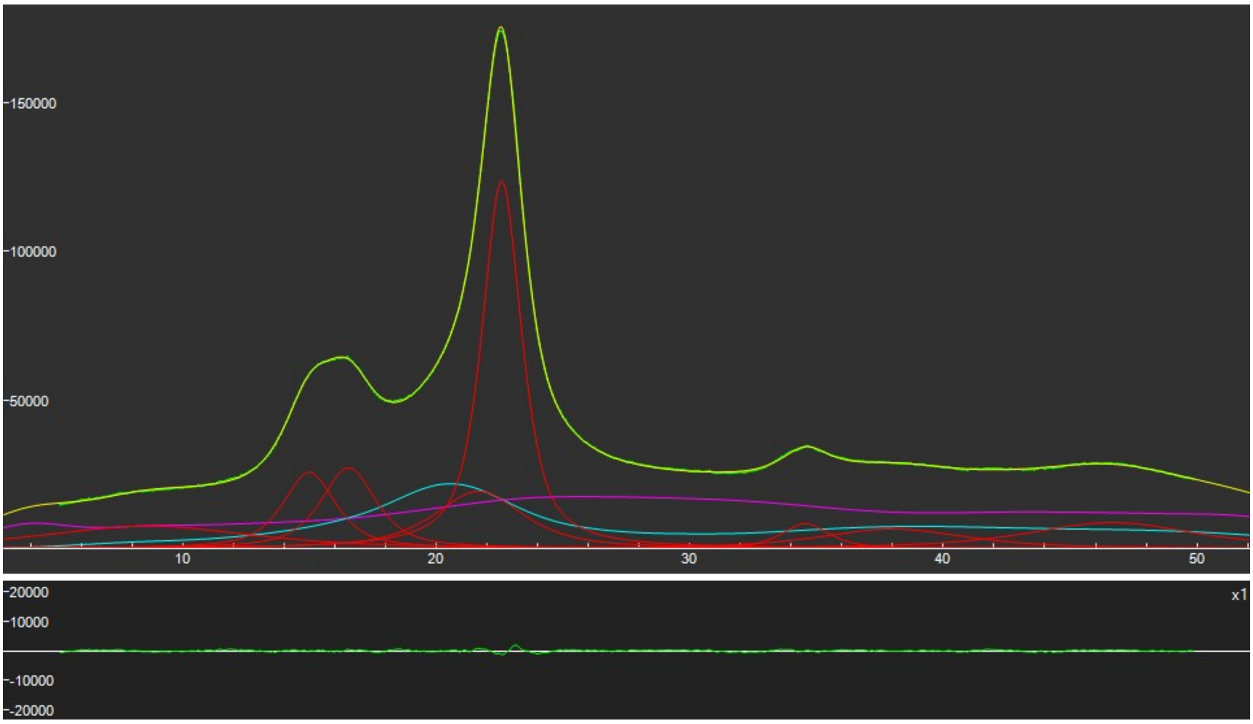

Fig. S6 Fitting of the WAXS diffractogram for the freeze-dried Enocell Kraft pulp (bleached hardwood pre-hydrolysis kraft pulp) sample in Fityk. Data & functions: raw data (green), fitted data (yellow), amorphous function (cyan), background (magenta), crystalline functions (red) and residual baseline error (green at the bottom of the figure).

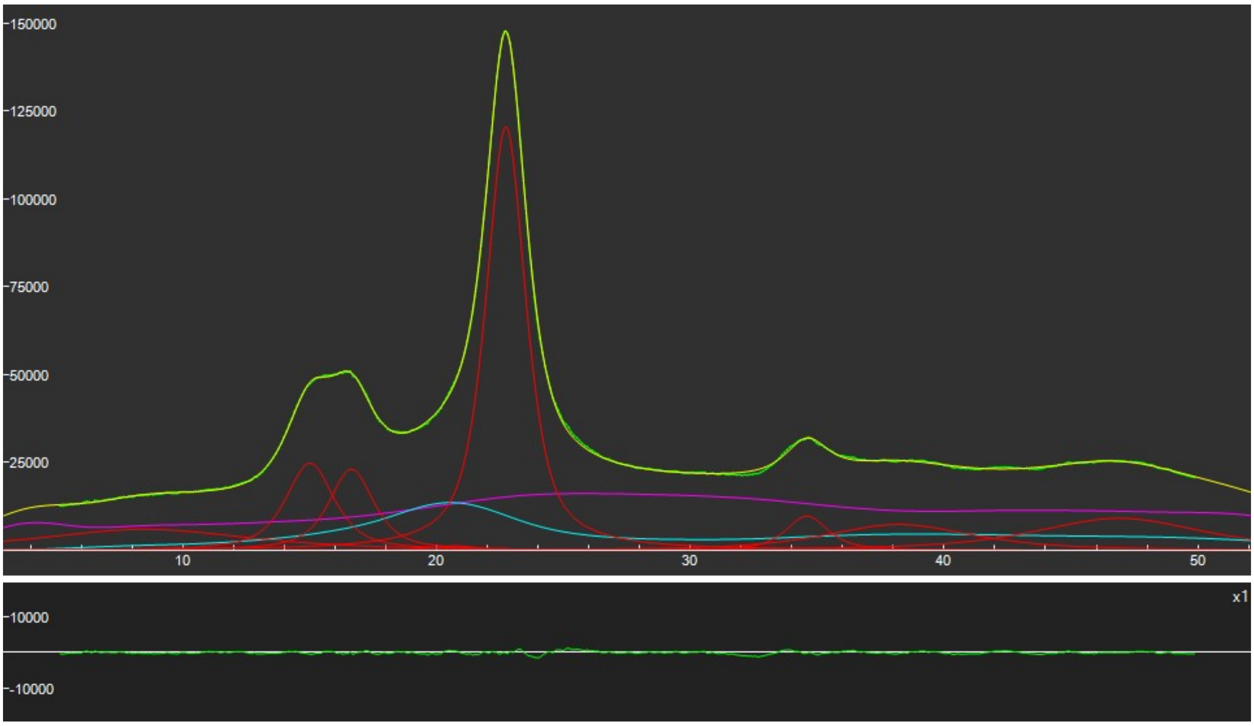

Fig. S7 Fitting of the WAXS diffractogram for the commercial FD-CNC (freeze-dried cellulose nanocrystals) sample, acetylated in system "gas-solid" for 6 days at ambient temperature, in Fityk. Data & functions: raw data (green), fitted data (yellow), amorphous function (cyan), background (magenta), crystalline functions (red) and residual baseline error (green at the bottom of the figure).

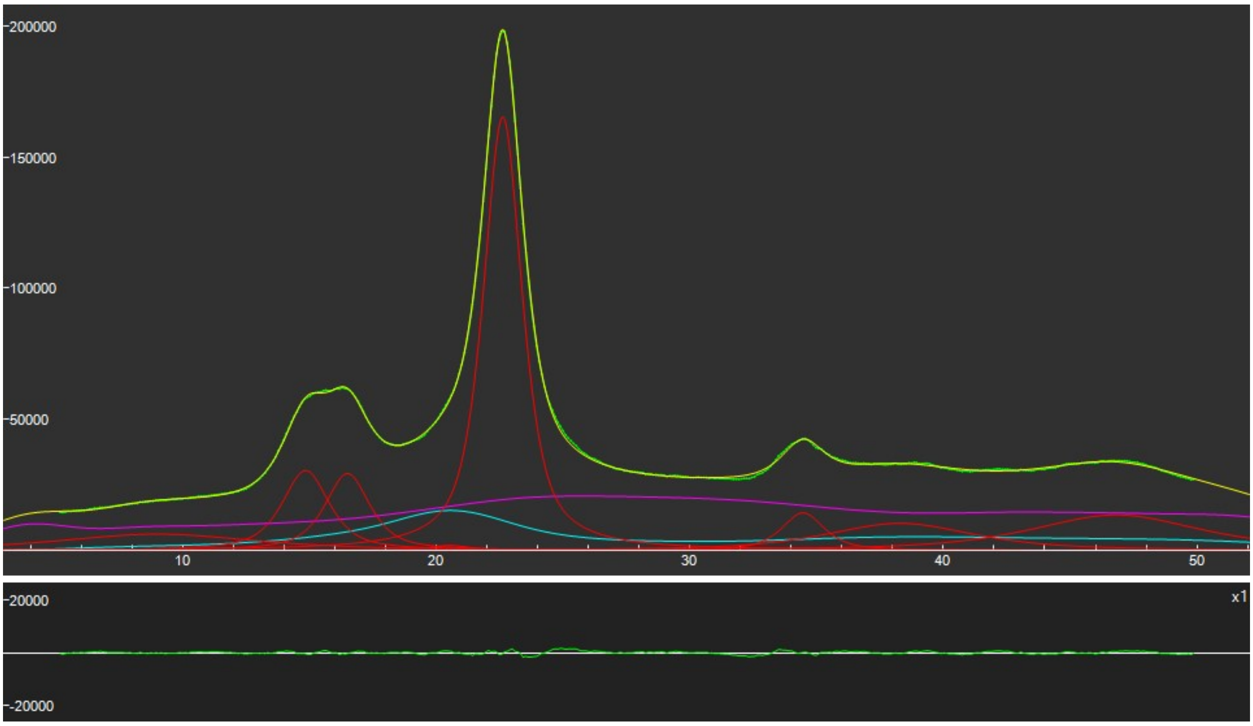

Fig. S8 Fitting of the WAXS diffractogram for the commercial FD-CNC (freeze-dried cellulose nanocrystals) sample, acetylated in system "gas-solid" for 15 days at ambient temperature, in Fityk. Data & functions: raw data (green), fitted data (yellow), amorphous function (cyan), background (magenta), crystalline functions (red) and residual baseline error (green at the bottom of the figure).

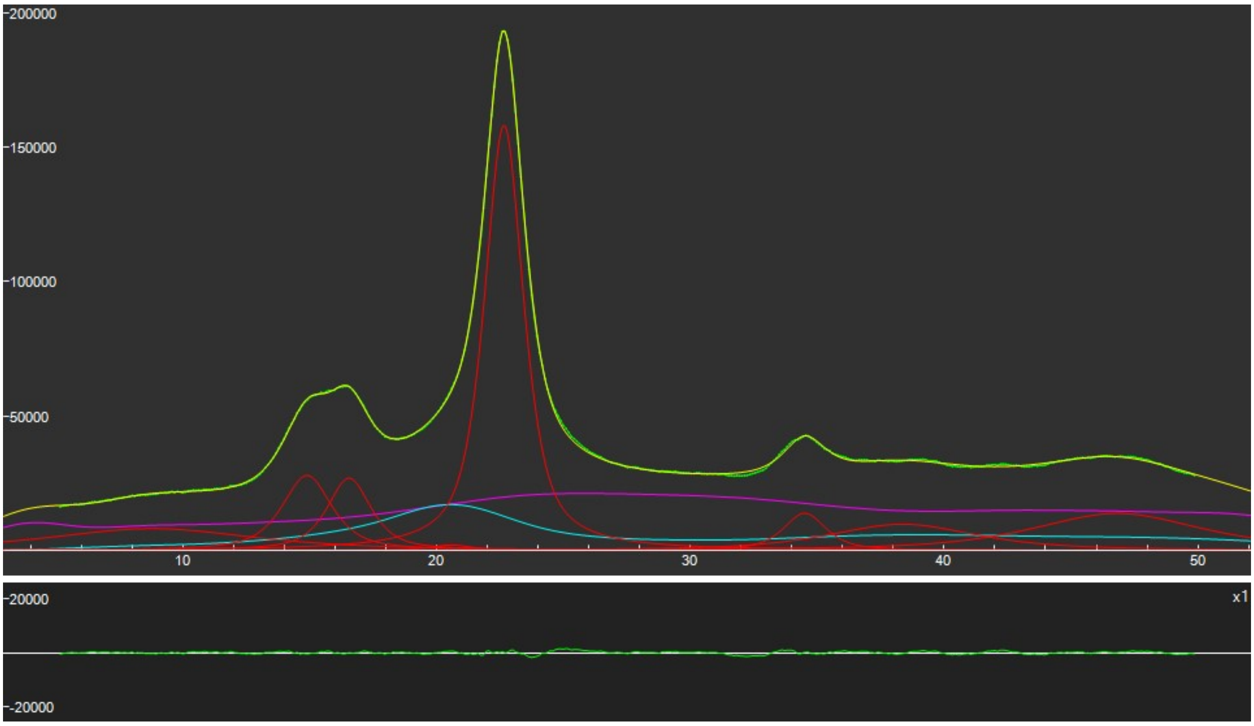

Fig. S9 Fitting of the WAXS diffractogram for the commercial FD-CNC (freeze-dried cellulose nanocrystals) sample, acetylated in system "gas-solid" for 32 days at ambient temperature, in Fityk. Data & functions: raw data (green), fitted data (yellow), amorphous function (cyan), background (magenta), crystalline functions (red) and residual baseline error (green at the bottom of the figure).

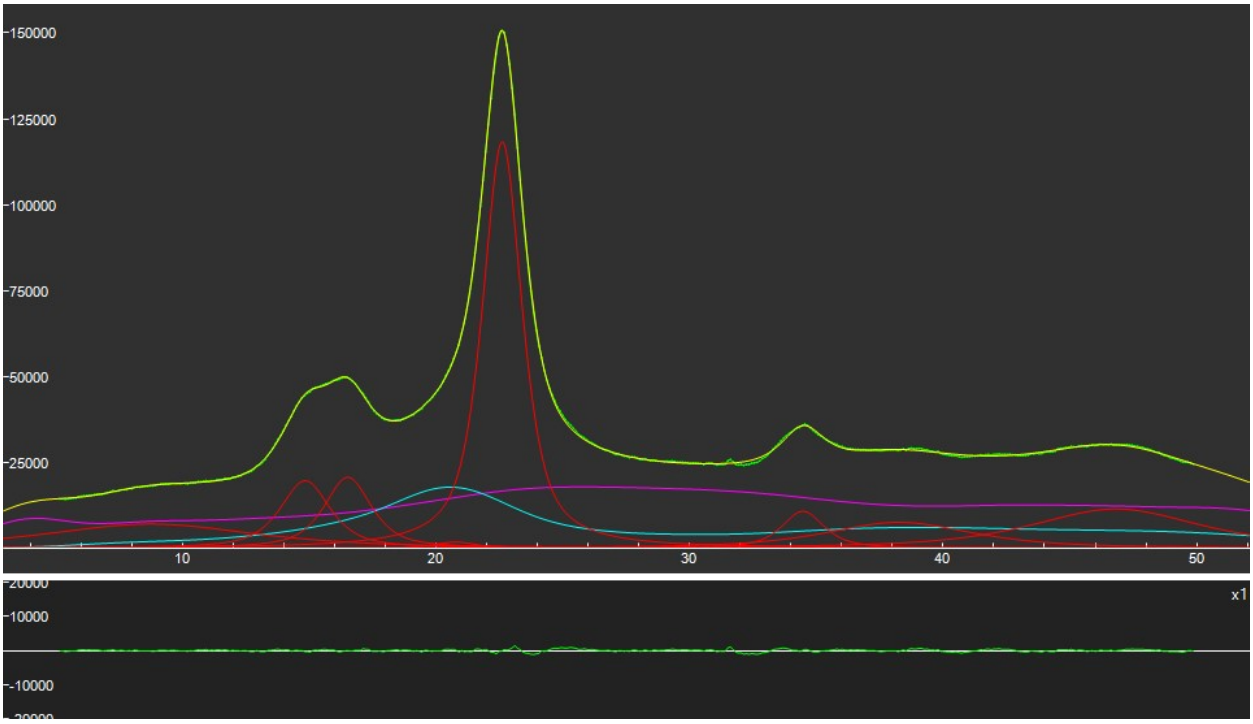

Fig. S10 Fitting of the WAXS diffractogram for the commercial FD-CNC (freeze-dried cellulose nanocrystals) sample, acetylated in system “gas-solid” for 6 days at 80 °C, in Fityk. Data & functions: raw data (green), fitted data (yellow), amorphous function (cyan), background (magenta), crystalline functions (red) and residual baseline error (green at the bottom of the figure).

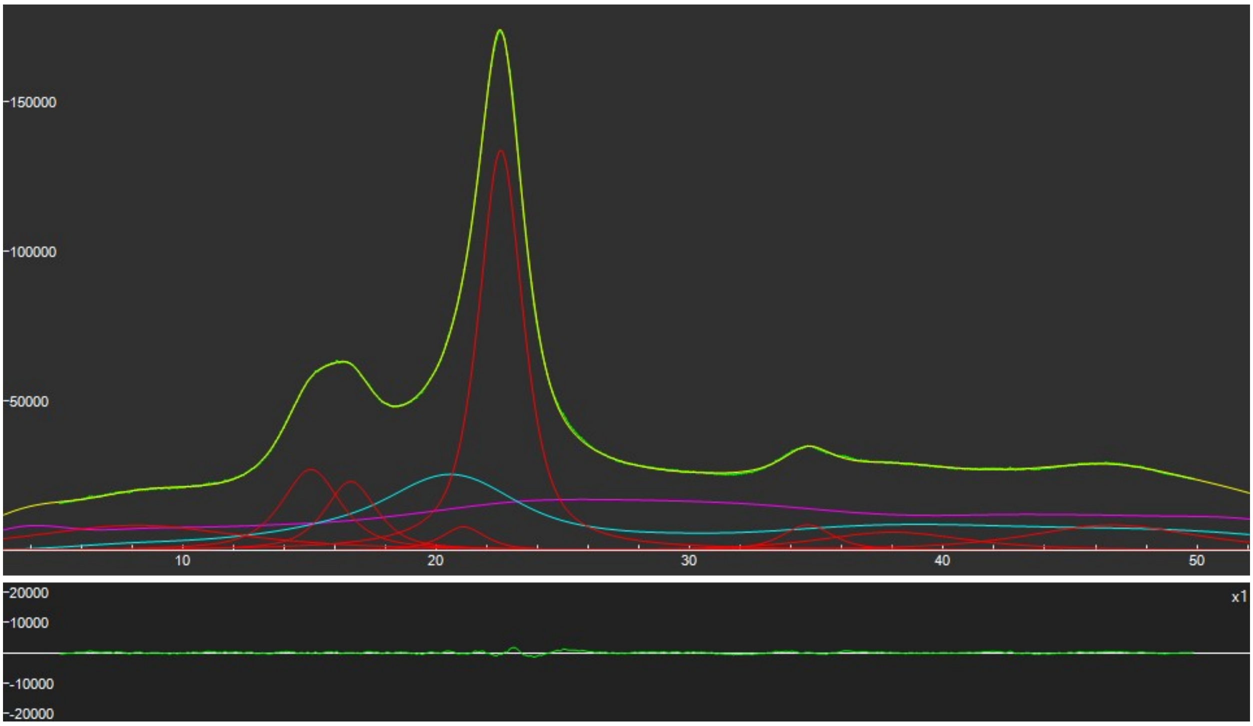

Fig. S11 Fitting of the WAXS diffractogram for the beech sulphite pulp (bleached hardwood sulphite pulp) sample, acetylated in system “gas-solid” for 6 days at ambient temperature, in Fityk. Data & functions: raw data (green), fitted data (yellow), amorphous function (cyan), background (magenta), crystalline functions (red) and residual baseline error (green at the bottom of the figure).

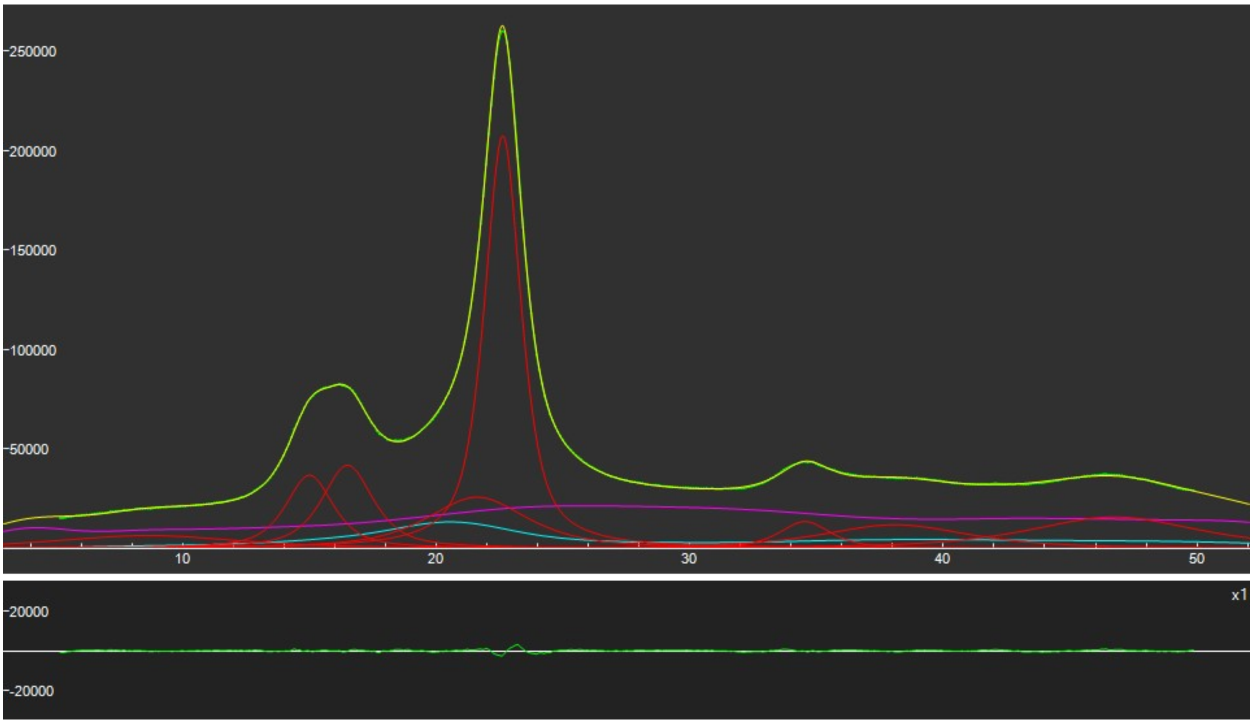

Fig. S12 Fitting of the WAXS diffractogram for the Enocell P-H kraft pulp (bleached hardwood pre-hydrolysis kraft pulp) sample, acetylated in system "gas-solid" for 6 days at ambient temperature, in Fityk. Data & functions: raw data (green), fitted data (yellow), amorphous function (cyan), background (magenta), crystalline functions (red) and residual baseline error (green at the bottom of the figure).

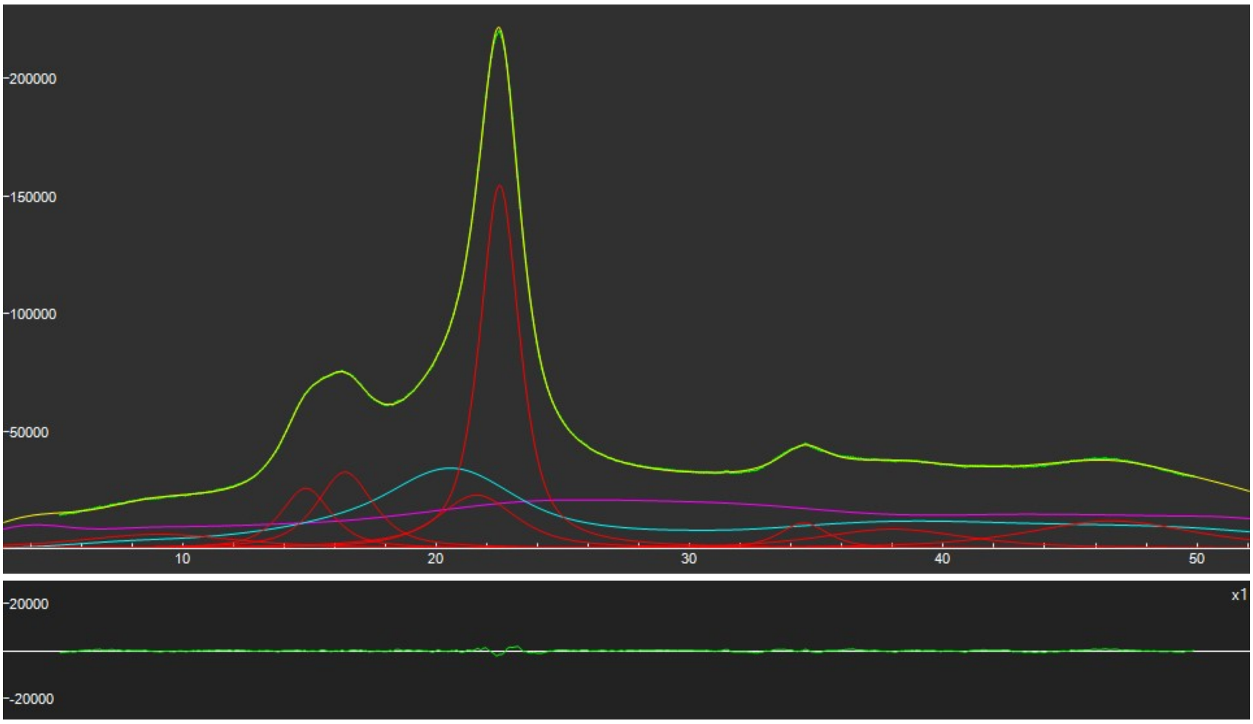

Fig. S13 Fitting of the WAXS diffractogram for the Enocell P-H kraft (bleached hardwood pre-hydrolysis kraft pulp) sample, acetylated in system "gas-solid" for 6 days at ambient temperature, in Fityk. Data & functions: raw data (green), fitted data (yellow), amorphous function (cyan), background (magenta), crystalline functions (red) and residual baseline error (green at the bottom of the figure).

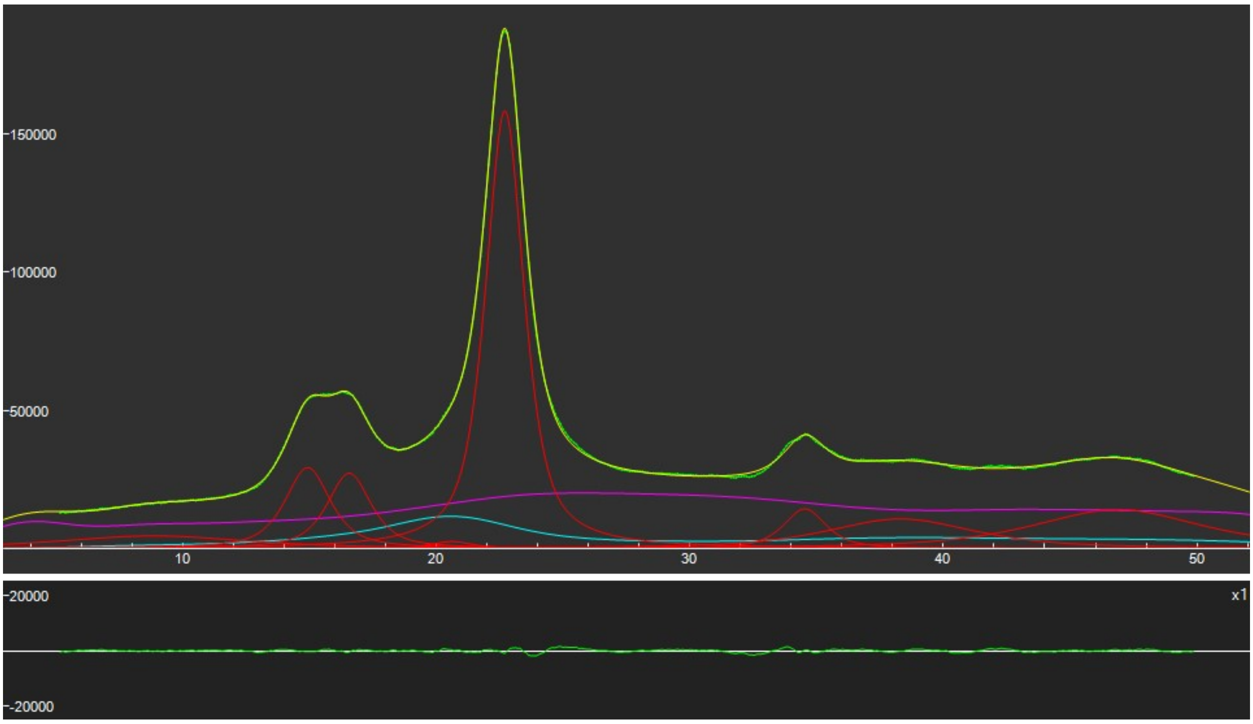

Fig. S14 Fitting of the WAXS diffractogram for the commercial FD-CNC (freeze-dried cellulose nanocrystals) sample, acetylated in system “liquid-solid” for 6 days at ambient temperature, in Fityk. Data & functions: raw data (green), fitted data (yellow), amorphous function (cyan), background (magenta), crystalline functions (red) and residual baseline error (green at the bottom of the figure).

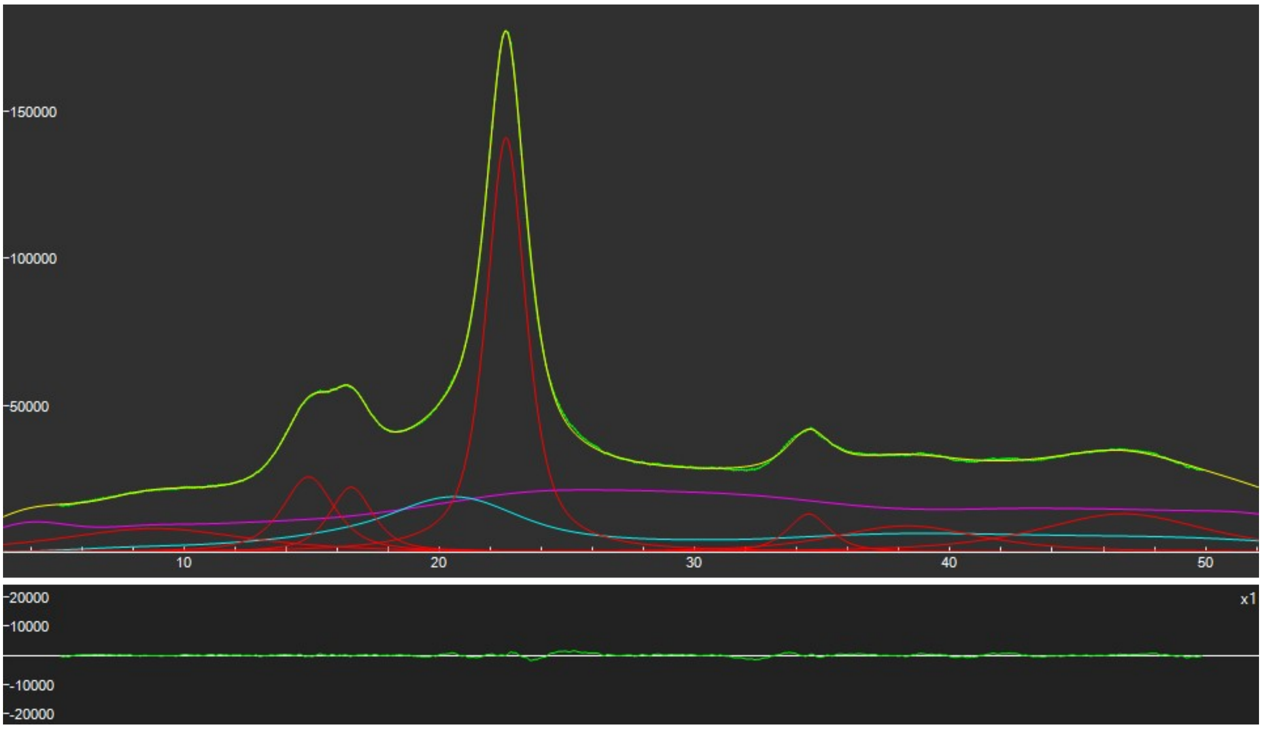

Fig. S15 Fitting of the WAXS diffractogram for the commercial FD-CNC (freeze-dried cellulose nanocrystals) sample, acetylated in system “liquid-solid” for 6 days at 80 °C, in Fityk. Data & functions: raw data (green), fitted data (yellow), amorphous function (cyan), background (magenta), crystalline functions (red) and residual baseline error (green at the bottom of the figure).

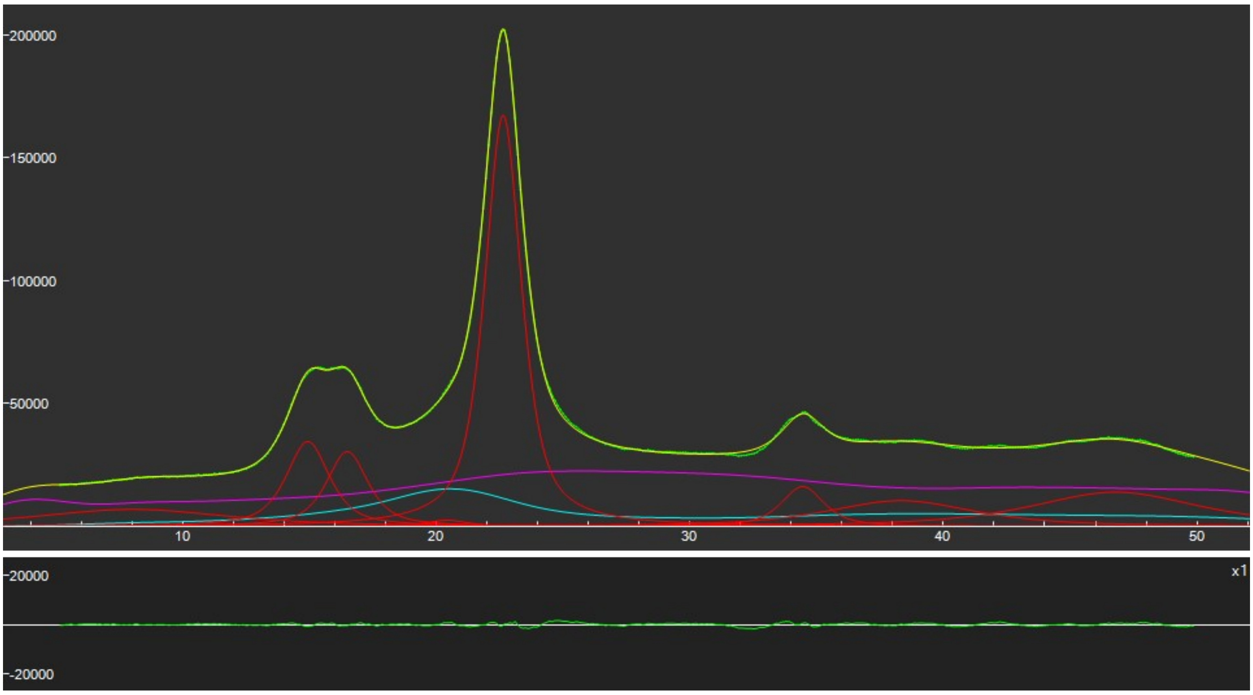

Fig. S16 Fitting of the WAXS diffractogram for the commercial FD-CNC (freeze-dried cellulose nanocrystals) sample, acetylated in system “liquid-solid” with DABCO for 6 days at ambient temperature, in Fityk. Data & functions: raw data (green), fitted data (yellow), amorphous function (cyan), background (magenta), crystalline functions (red) and residual baseline error (green at the bottom of the figure).

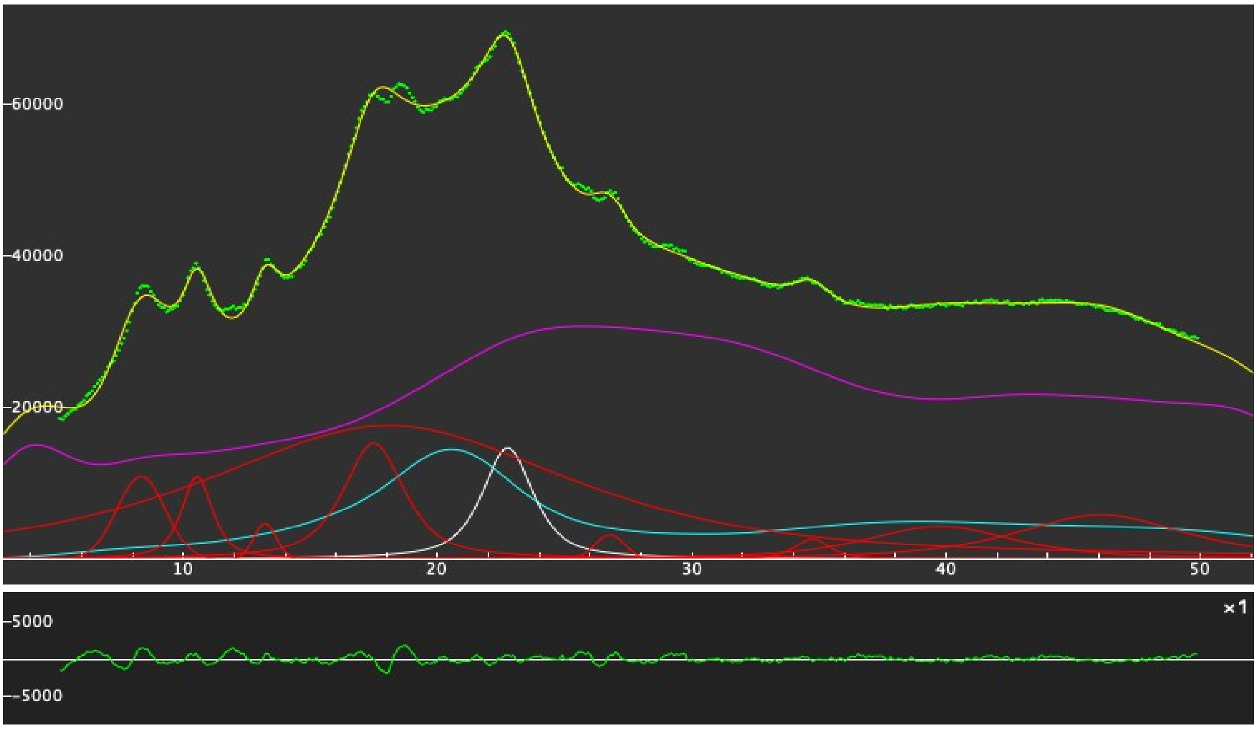

Fig. S17 Fitting of the WAXS diffractogram for the commercial FD-CNC (freeze-dried cellulose nanocrystals) sample, acetylated in system “liquid-solid” with DABCO for 6 days at 80 °C, in *Fityk*. Data & functions: raw data (green), fitted data (yellow), amorphous function (cyan), background (magenta), CTA functions (red), cellulose I_{β} 200 function (white) and residual baseline error (green at the bottom of the figure).

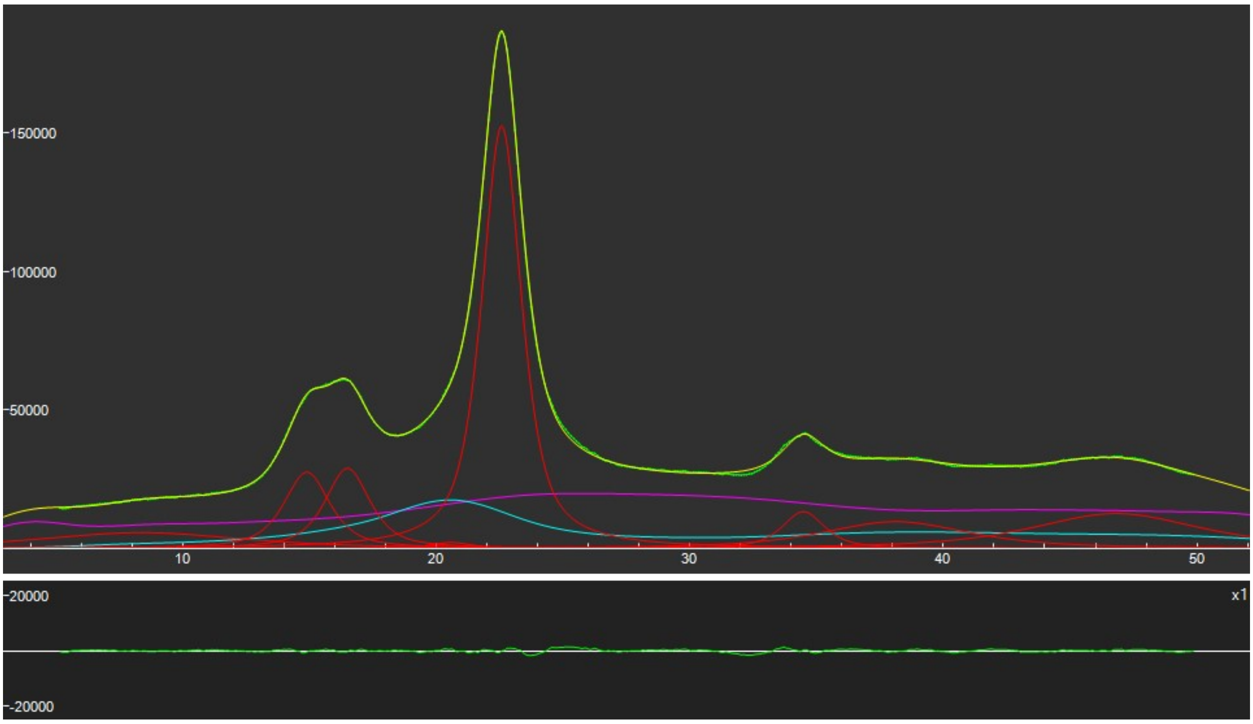

Fig. S18 Fitting of the WAXS diffractogram for the commercial FD-CNC (freeze-dried cellulose nanocrystals) sample, acetylated in system “liquid-solid” with pyridine for 6 days at ambient temperature, in Fityk. Data & functions: raw data (green), fitted data (yellow), amorphous function (cyan), background (magenta), crystalline functions (red) and residual baseline error (green at the bottom of the figure).

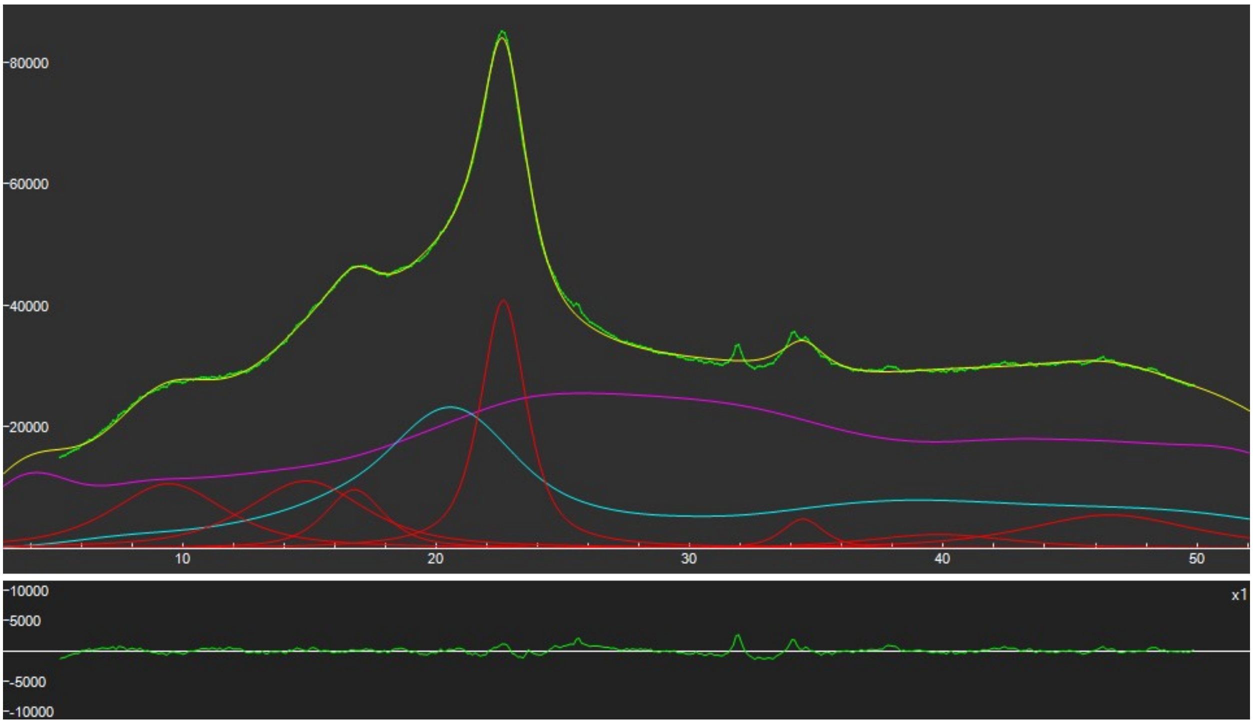

Fig. S19 Fitting of the WAXS diffractogram for the commercial FD-CNC (freeze-dried cellulose nanocrystals) sample, acetylated in system “liquid-solid” with pyridine for 6 days at 80 °C, in Fityk. Data & functions: raw data (green), fitted data (yellow), amorphous function (cyan), background (magenta), crystalline functions (red) and residual baseline error (green at the bottom of the figure).

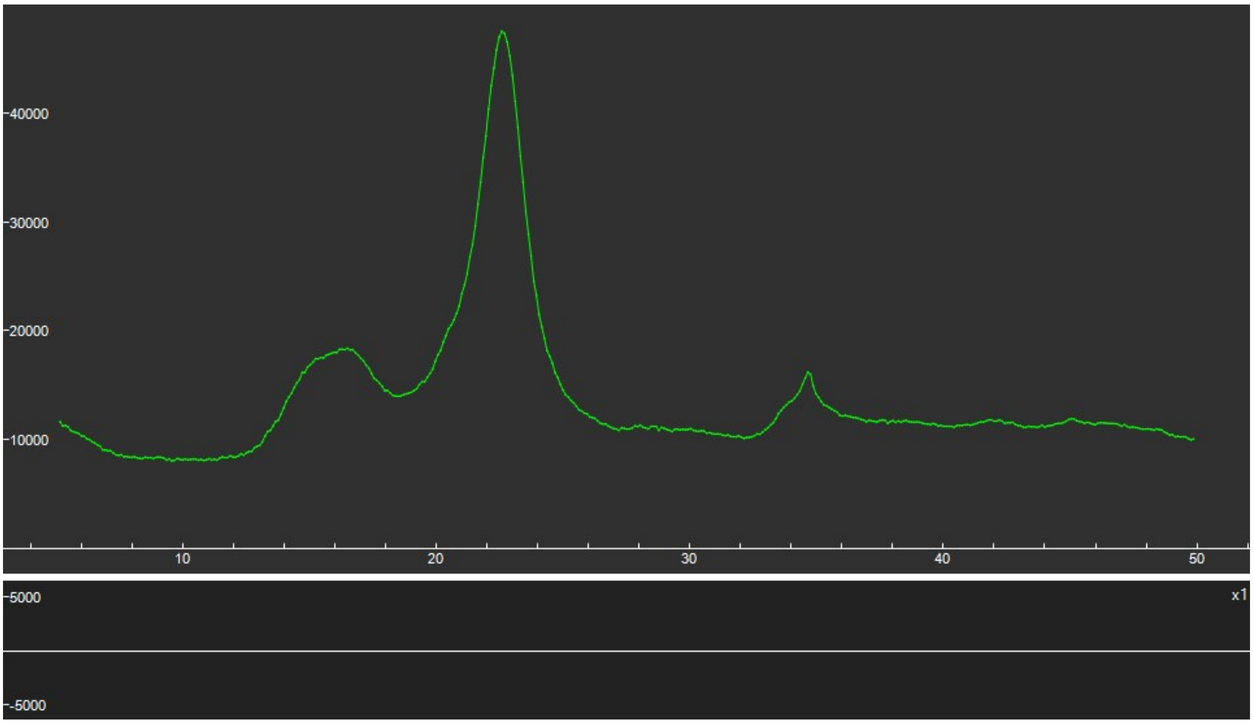

Fig. S20 WAXS diffractogram for the commercial spray-dried CNCs (SD-CNC) sample.

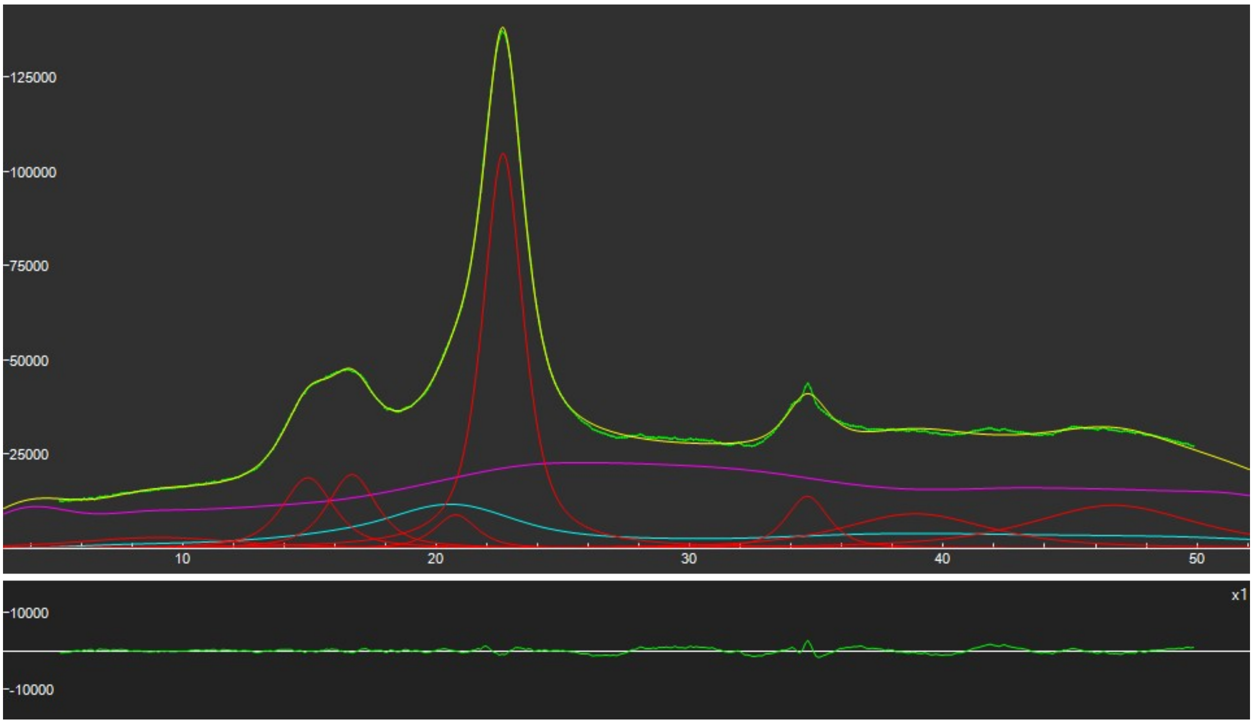

Fig. S21 Fitting of the WAXS diffractogram for the commercial SD-CNC (spray-dried cellulose nanocrystals) sample, acetylated in system "gas-solid" for 6 days at ambient temperature, in Fityk. Data & functions: raw data (green), fitted data (yellow), amorphous function (cyan), background (magenta), crystalline functions (red) and residual baseline error (green at the bottom of the figure).

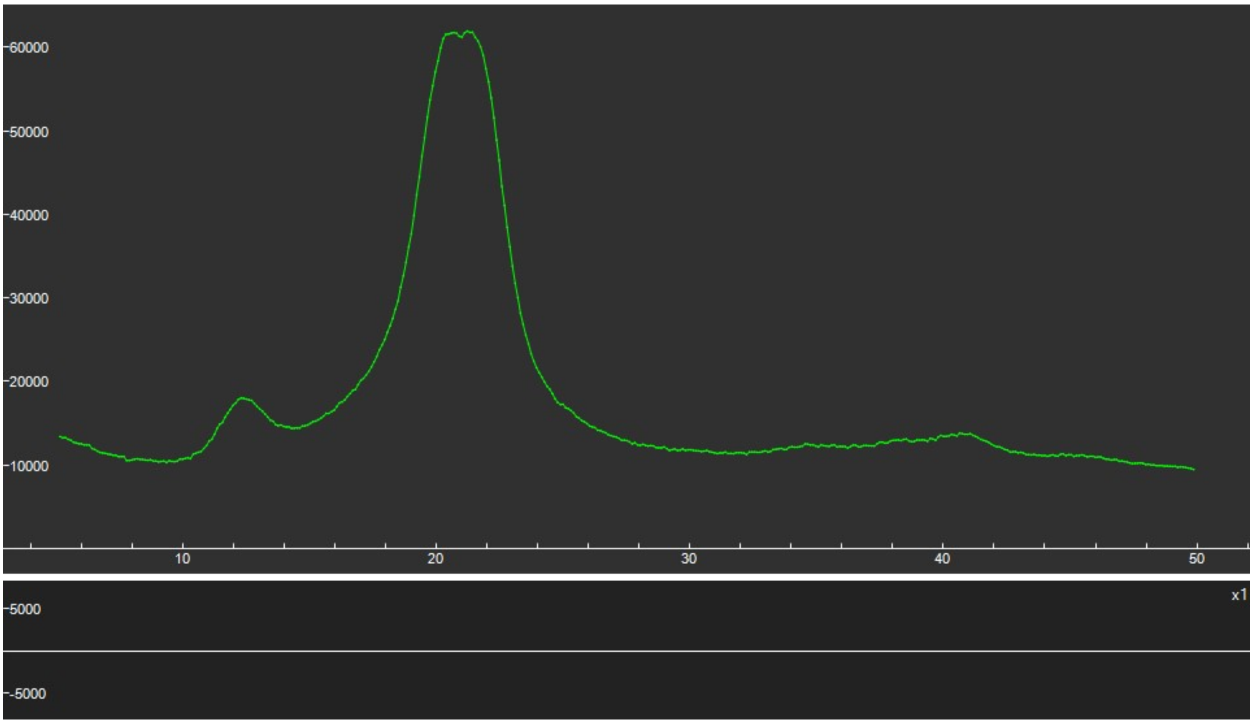

Fig. S22 WAXS diffractogram for the IONCELL fibre sample.

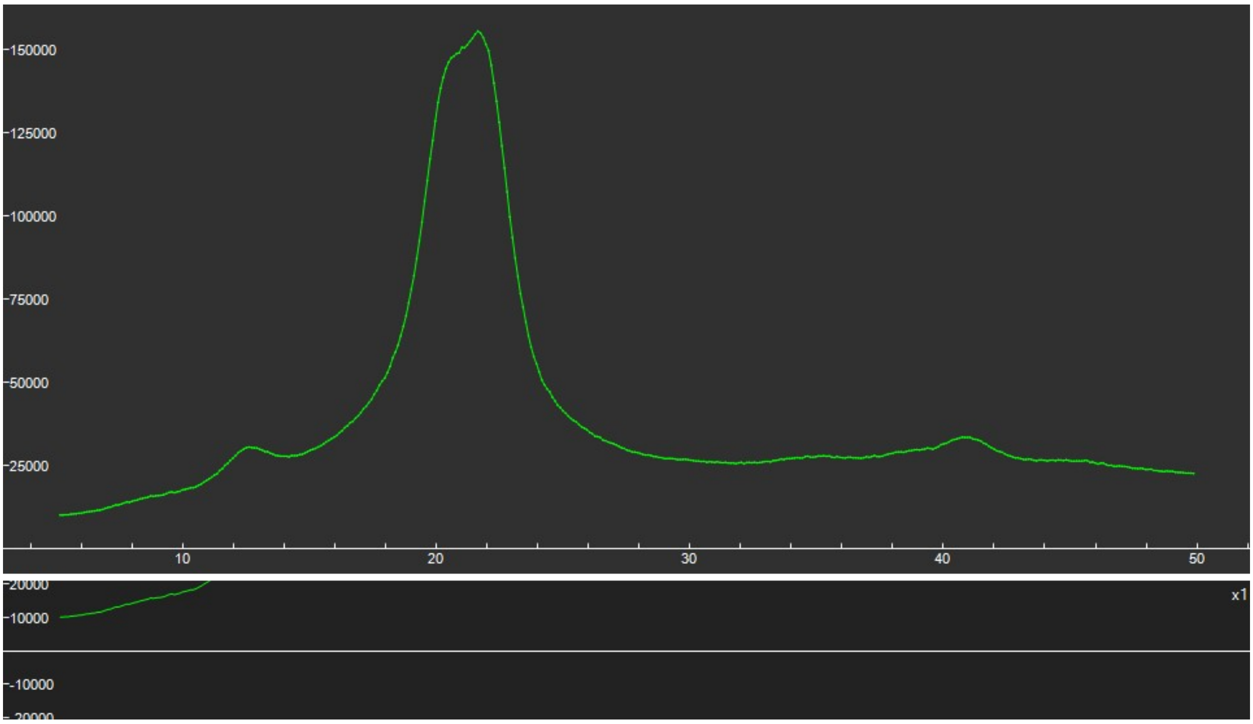

Fig. S23 WAXS diffractogram for the IONCELL fibre sample, acetylated in system "gas-solid" for 6 days at 80 °C.

Rough periodic plane sizes were calculated using the Scherrer equation [Eq.(1)]:

$$L = \frac{K\lambda}{(\beta cos\theta)} \tag{1}$$

which is commonly used for determining the dimension (L, nm) of cellulose crystallites, where β is the full width half maximum (FWHM) for particular periodic plane, K (the Scherrer constant) is 0.94 for spherical crystallites with cubic symmetry. λ is the X-ray wavelength (1.54178 Å in our case for Cu Kα) and θ is 2θ / 2 (in radians). The FWHM values were determined from the Gaussian functions, in Fityk. The results are given in the main text. The crystallite sizes for the (200) plane for each sample were calculated (Table S1).

Table S1. Crystallite sizes calculated for the samples, using the FWHM values for the (200) planes.

Sample	(200) FWHM (°)	(200) Crystallite size (nm)	CI (%)
FD-CNC	1.88	4.50	57
FD-CNC-GS-6D RT	1.88	4.50	68
FD-CNC-GS-15D RT	1.84	4.60	73
FD-CNC-GS-32D RT	1.84	4.60	68
FD-CNC-GS-6D 80 °C	1.87	4.53	63
FD-CNC-LS-6D RT	1.82	4.65	77
FD-CNC-LS-6D 80 °C	1.86	4.55	65
FD-CNC-LS-6D DABCO, RT	1.73	4.89	72
FD-CNC-LS-6D DABCO, 80 °C	-	-	-
FD-CNC-LS-6D Py, RT	1.87	4.53	69
FD-CNC-LS-6D Py, 80 °C	2.16	3.93	42

2. Crystallite Models

For estimation of the bulk DS values for full surface coverage (6-OH acetylation only), there are 4 important elementary fibrillar cross-section models to consider, based on diffraction, molecular dynamics and NMR experiments (Figure S24) (Wang *et al.*, 2015; Oehme *et al.*, 2015; Paajanen *et al.*, 2019; Fernandes *et al.*, 2011). These are the 18-chain hexagonal (DS = 0.33), 24-chain rhomboid (DS = 0.33), 24-chain hexagonal (DS = 0.25) and 36-chain hexagonal (DS = 0.22). It is assumed that the woody microfibril consists of most probably 18 or 24 individual cellulose chains, with the softwood model the 24-chain rhomboid model (Fernandes *et al.*, 2011). Based on these models and different chain assemblies (Figures S24 A-D) the maximum degree of substitution of the C6-OH is estimated to ranging from 0.22 to 0.33. An approximate maximum of 0.33 can is assumed.

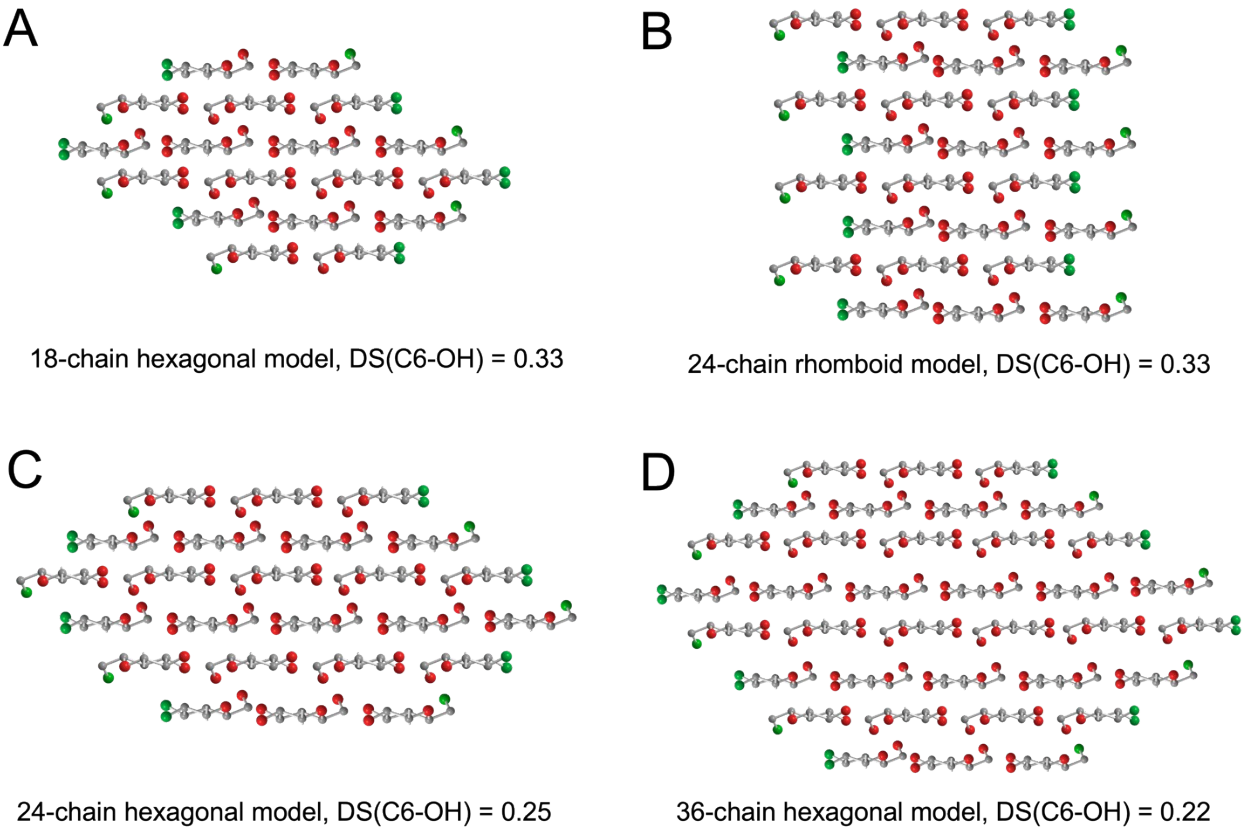

Figure S24: Superstructure of the cellulose microfibril based on different chain models.

3. NMR supplementary data

3.1. NMR sample preparation

To prepare the samples for NMR analysis, typically 50 mg of dried sample is added to a sealable sample vial and made up to 1 g, by addition of stock [P_{4444}][OAc]:DMSO-d_6 (20:80 wt%) electrolyte solution (King et al. 2018; Koso et al. 2020). The samples were magnetically stirred at RT until they were visibly clear. This typically

takes less than 1 hr period and even a few minutes for some samples. If the samples did not go clear during that time, the temperature was increased to 65 °C. All further NMR experiments were recorded on a Bruker AVANCE NEO 600 MHz spectrometer equipped with a 5 mm SmartProbe™.

3.2. Diffusion-edited ^{1}H experiments

The diffusion-edited ^{1}H experiment used a 1D bipolar-pulse pair with stimulated echo (BPPSTE) (Wu et al. 1995) diffusion-ordered spectroscopy (DOSY) pulse sequence (Bruker pulse program 'ledbpgp2s1d'), with 3 s relaxation delay (d1), 0.5 s acquisition time (aq), 16 dummy scans (ds) and 512 transient scans (ns), a sweep-width (sw) of 20 ppm with the transmitter offset on 6.1 ppm (o1p), a diffusion time (d20) of 200 ms, a gradient recovery delay (d16) of 0.2 ms, an eddy current delay (d21) of 5 ms, , a diffusion gradient pulse duration (p30) of 2.5 ms and a z-gradient strength (gpz6) of 90% at 50 G/cm (probe z-gradient strength). These conditions are specific to the Bruker AVANCE NEO 600 MHz - SmartProbe™ system.

3.3. Multiplicity-Edited HSQC Experiments

The HSQC experiments on cellulose samples used either a multiplicity-edited phase sensitive HSQC sequence with echo/antiecho-TPPI gradient selection (Bruker pulse program 'hsqcedetgp') (Willker et al. 1993) or a sensitivity improved multiplicity-edited phase sensitive HSQC sequence with echo/antiecho-TPPI gradient selection and adiabatic pulses (Bruker pulse program 'hsqcedetgpsisp2.2'), for increased sensitivity (Willker et al. 1993; Palmer III et al. 1991; Kay et al. 1992; Schleucher et al. 1994). Typical parameters are as follows: spectral widths (sw) were 13.03 and 165 ppm, with transmitter offsets (o1p) of 6.18 and 75 ppm, for ^{1}H and ^{13}C dimensions, respectively. The time-domain size (td1) in the indirectly detected ^{13}C-dimension (f1) was 512 or 1024, corresponding to 256 increments, or 512 t_1-increments for the real spectrum. There were 16 dummy scans (ds), typically 8 (or multiples of 8) scans (ns), an acquisition time (aq) of 0.065 s for f2 and a relaxation delay of 1.5 s. Window functions were typically sine squared (90 °) in f1 and f2

3.4. DS determination from ^{1}H NMR spectral data

As all the samples were completely solubilised in $[P_{4444}][OAc]$:DMSO-d_6 electrolyte, it was possible to determine the bulk degree of acetylation on the cellulosic backbone directly from the ^{1}H NMR spectra by peak fitting and calculation according to the equation [Eq. (2)]

$$DS = \frac{I_A/3}{I_C/7} \qquad (2)$$

Here, I_A is the acetate signal peak area (~ 1.8 – 2.2 ppm) and I_C is the cellulose backbone combined signal peak area (~ 2.8 – 5.5 ppm). "3" and "7" are the total number of protons for abovementioned fragments of acetate and cellulose, respectively. As we do not use the diffusion-edited ^{1}H NMR for DS estimations, but rather quantitative ^{1}H NMR, correction coefficient (del Cerro et al. 2020) is not applicable.

All the spectra were recorded with a 10 s relaxation delay (30 ° pulse) and 16 or 32 transients were collected. The spectra were calibrated and phased in Bruker TopSpin (4.0.5). MNova (10.0.2) was used to convert the spectral data into .xy format, for Fityk processing. An aggressive spline basline correction was performed before peak fitting the corresponding H1-H6 and acetate peak regions. Examples of spline baseline correction and peak fitting are shown in **Figures S25-27**.

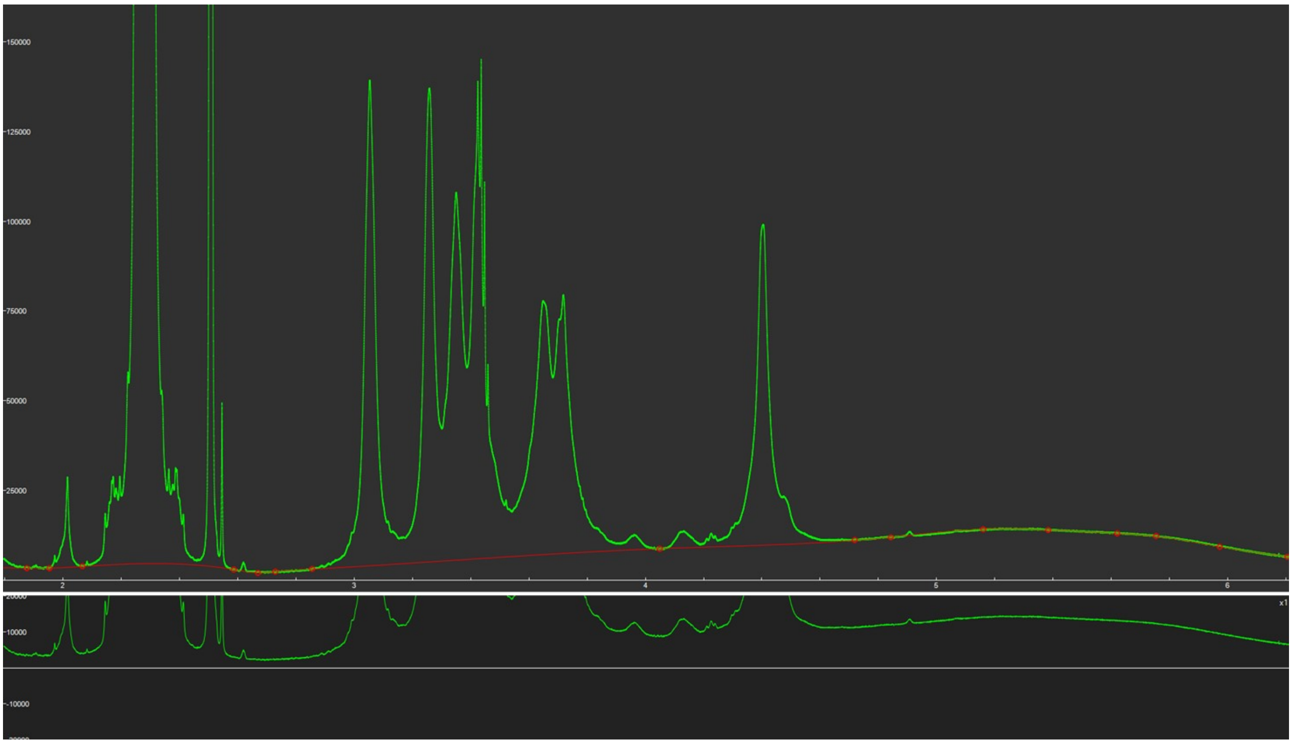

Fig. S25 Example of the spline baseline correction for the FD-CNCs sample, acetylated in system "gas-solid" for 6 days at ambient temperature, in *Fityk*.

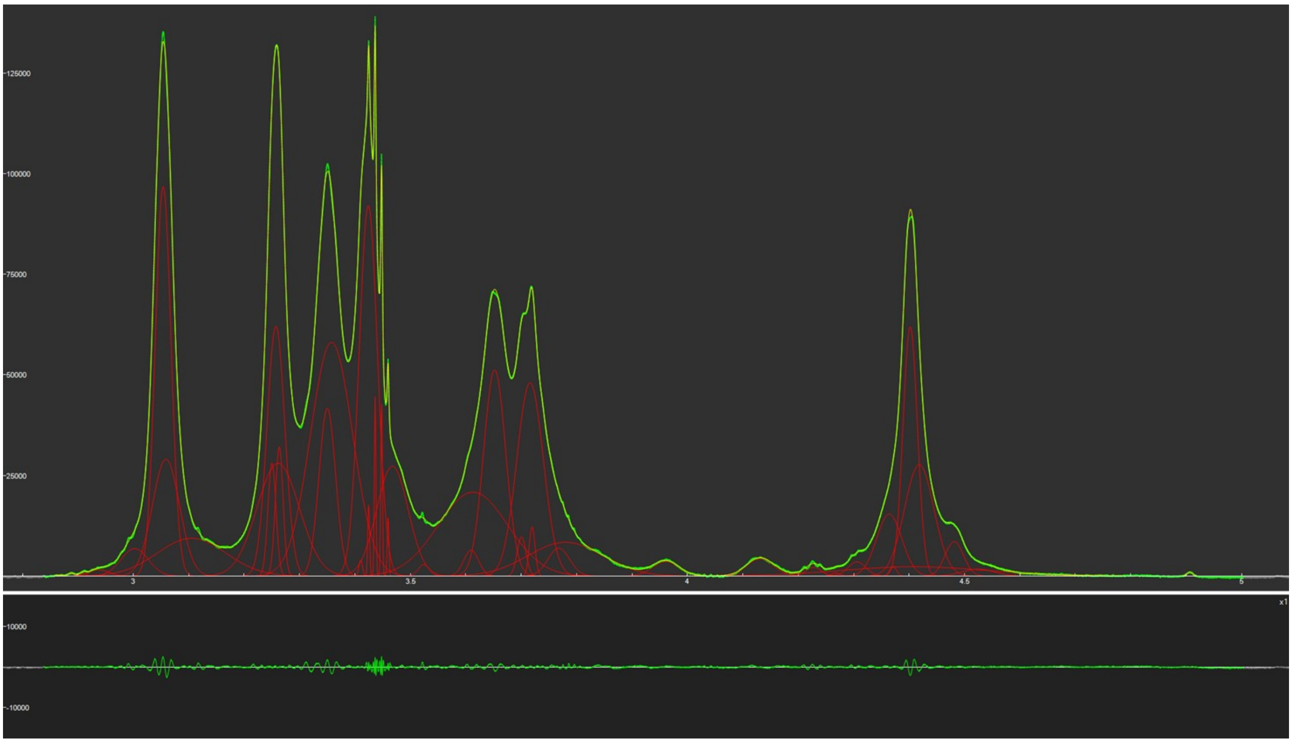

Fig. S26 Example of the Gaussian deconvolution of the AGU (cellulose) peaks for the FD-CNCs sample, acetylated in system "gas-solid" for 6 days at ambient temperature, in *Fityk*.

3.5. Regioselectivity determination from ^{1}H NMR spectral data

6 vs 2 vs 3-OH acetylation regioselectivity could be determined through peak-fitting of the acetate region (~2 ppm) from the quantitative ^{1}H spectra. Application of spline baseline correction (see above) and then application of Gaussian guesses and automatic fitting usually gives nice defined peak volumes corresponding to the different acetate signals. Some manual fitting of the parameters may be required, e.g. to prevent the automatic fitting of too large Gaussians which may encompass the whole acetate region.

The signals for the high DS cellulose acetate (DS 2.4) are very characteristic and the 3 main peaks of cellulose triacetate (CTA) clearly visible, with a little variation in peak positioning corresponding to AGUs with mono and diacetate (**Figure S27a**). The regioselectivity is defined as the percentage of 6-OAc (mono acetate) vs the sums of the 2-OAc, 3-OAc and 6-OAc (CTA). This can be calculated from the sums of the Gaussian peak volumes fitted for each region; the peaks for each region are assigned by having peak centers (ppm) laying within defined regions, as illustrated for the solid-liquid acetylated samples at 80 °C (**Figure S27b-c**).

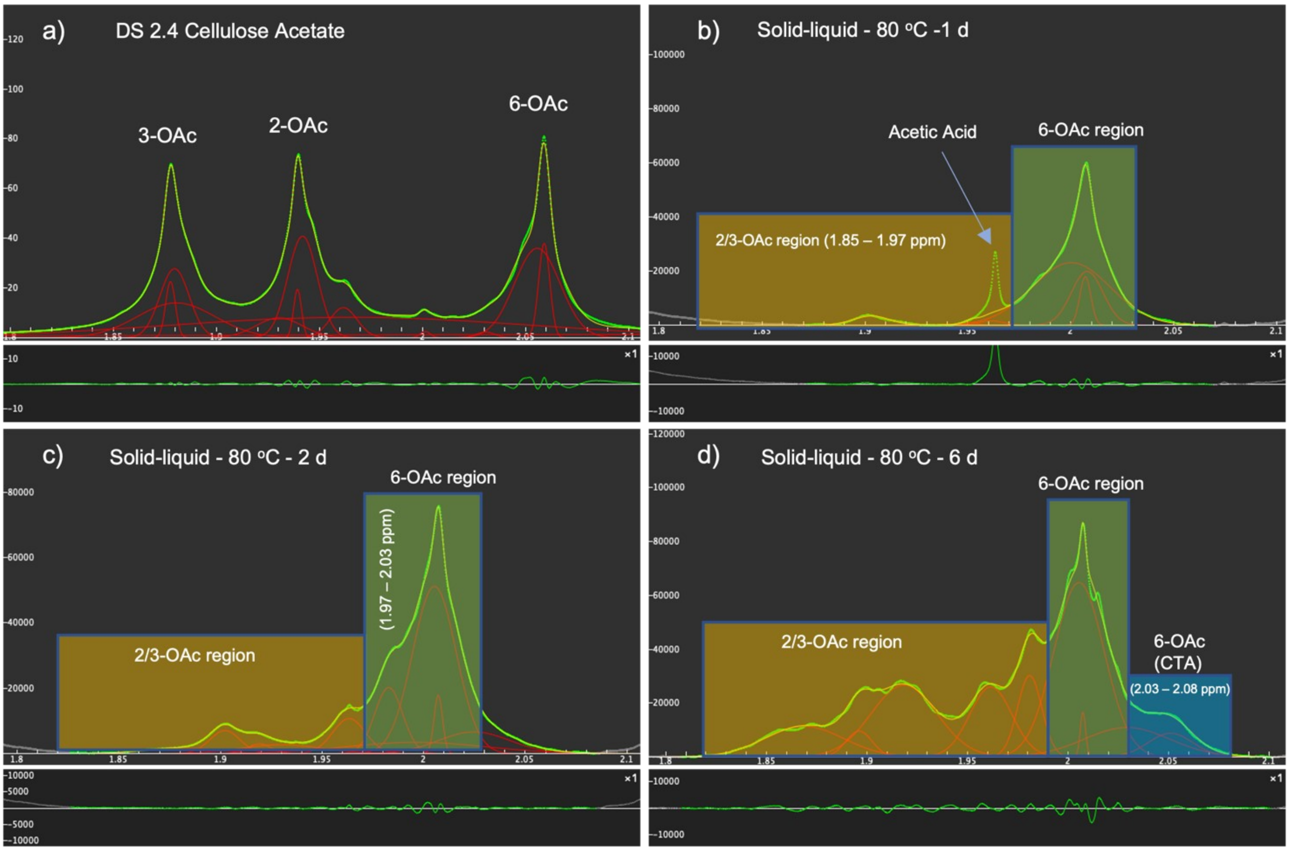

Fig. S27 Example of peak-fitting using *Fityk*, and the appropriate peak regions, for determination of 6-OH acetylation regioselectivity and DS determination.

Table S2. Bulk degree of acetylation for gas-phase reactions on the different substrates.

Starting material	Conditions	Catalyst	Degree of substitution (DS)[1)]	Product allomorph CI, %
FD-CNCs[2)]	gas-solid, rt, 1 day	–	0.007	–
FD-CNCs	gas-solid, rt, 2 days	–	0.010	–
FD-CNCs	gas-solid, rt, 6 days	–	0.029	Cellulose I 68
FD-CNCs	gas-solid, rt, 15 days	–	0.116	Cellulose I 73
FD-CNCs	gas-solid, rt, 32 days	–	0.290	Cellulose I 68
FD-CNCs	gas-solid, 80 °C, 1 day	–	0.120	–
FD-CNCs	gas-solid, 80 °C, 2 days	–	0.226	–
FD-CNCs	gas-solid, 80 °C, 6 days	–	0.405	Cellulose I 63
SD-CNCs[3)]	gas-solid, rt, 6 days	–	–	Cellulose I 75
BH-S-P[4)]	gas-solid, rt, 6 days	–	0.020	Cellulose I 59
BH-PHK-P[5)]	gas-solid, rt, 6 days	–	0.007	Cellulose I 80
ND-BH-PHK-P[6)]	gas-solid, rt, 6 days	–	0.005	Cellulose I 60
CNC-AG[7)]	gas-solid, rt, 6 days	–	0.136	Cellulose I –
BC-AG[8)]	gas-solid, rt, 6 days	–	–	Cellulose I –
IONCELL-F[9)]	gas-solid, 80 °C, 6 days	–	0.141	Cellulose II –

Table S3. Bulk degree of acetylation for liquid-phase reactions of FD-CNCs without and with the presence of catalyst.

Starting material	Conditions	Catalyst	DS (NMR)	Product allomorph CI, %
FD-CNCs	liq-solid, rt, 1 day	–	0.012	–
FD-CNCs	liq-solid, rt, 2 day	–	0.019	–
FD-CNCs	liq-solid, rt, 6 day	–	0.021	Cellulose I 77
FD-CNCs	liq-solid, 80 °C, 1 day	–	0.076	–
FD-CNCs	liq-solid, 80 °C, 2 days	–	0.154	–
FD-CNCs	liq-solid, 80 °C, 6 days	–	0.426	Cellulose I 65
FD-CNCs	liq-solid, rt, 1 day	DABCO	0.013	–
FD-CNCs	liq-solid, rt, 2 day	DABCO	0.021	–
FD-CNCs	liq-solid, rt, 6 day	DABCO	0.042	Cellulose I 72
FD-CNCs	liq-solid, 80 °C, 1 day	DABCO	0.470	–
FD-CNCs	liq-solid, 80 °C, 2 days	DABCO	–	–
FD-CNCs	liq-solid, 80 °C, 6 days	DABCO	–	Cellulose I 5 + CTA
FD-CNCs	liq-solid, rt, 1 day	Pyridine	0.037	–
FD-CNCs	liq-solid, rt, 2 day	Pyridine	0.043	–
FD-CNCs	liq-solid, rt, 6 day	Pyridine	0.063	Cellulose I 69
FD-CNCs	liq-solid, 80 °C, 1 day	Pyridine	0.314	–
FD-CNCs	liq-solid, 80 °C, 2 days	Pyridine	0.594	–
FD-CNCs	liq-solid, 80 °C, 6 days	Pyridine	2.025	Cellulose I 42

3.6. The NMR data

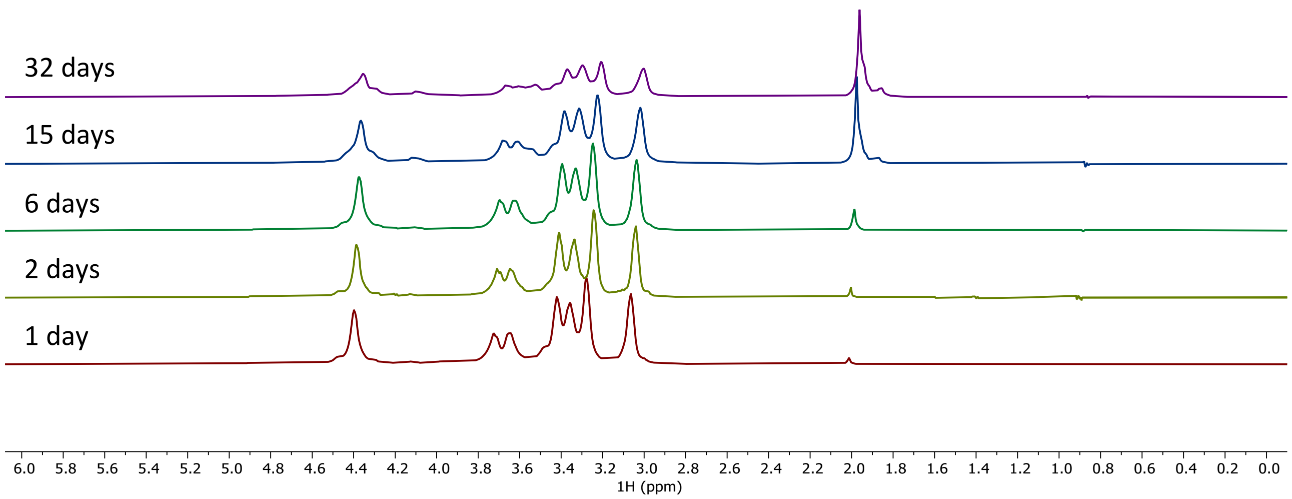

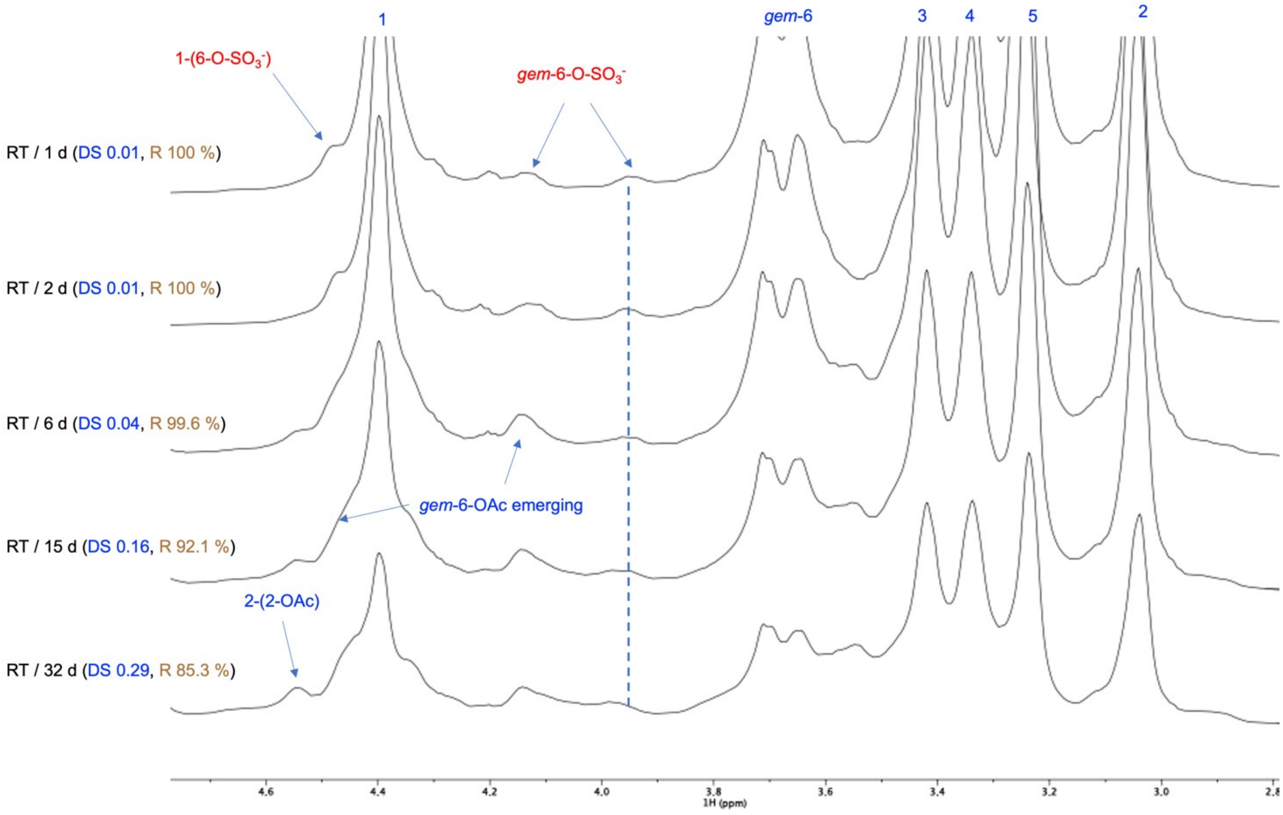

Fig. S28 Expanded diffusion-edited ^{1}H NMR spectra for FD-CNC samples, acetylated in system "gas-solid" at ambient temperature for 1, 2, 6, 15 and 32 days.

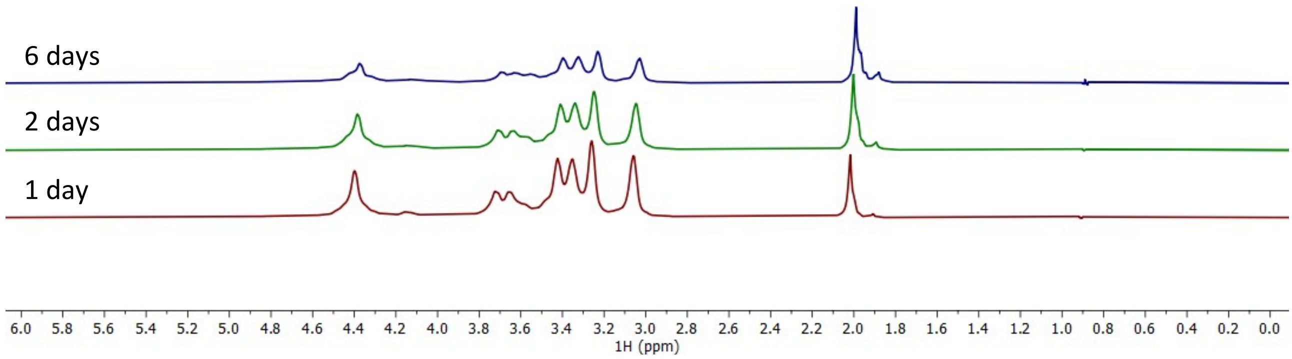

Fig. S29 Expanded diffusion-edited ^{1}H NMR spectra for FD-CNC samples, acetylated in system "gas-solid" at 80 °C for 1, 2 and 6 days.

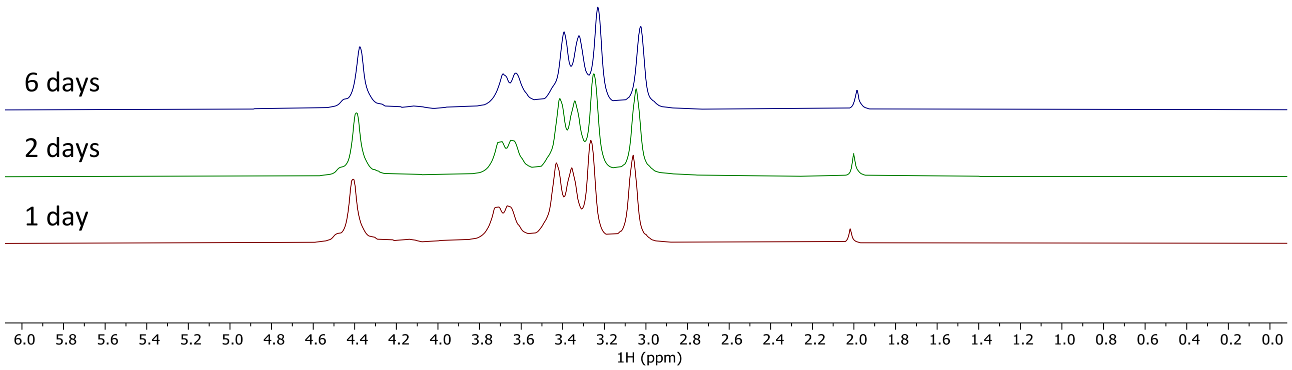

Fig. S30 Expanded diffusion-edited ^{1}H NMR spectra for FD-CNC samples, acetylated in system "liquid-solid" at ambient temperature for 1, 2 and 6 days.

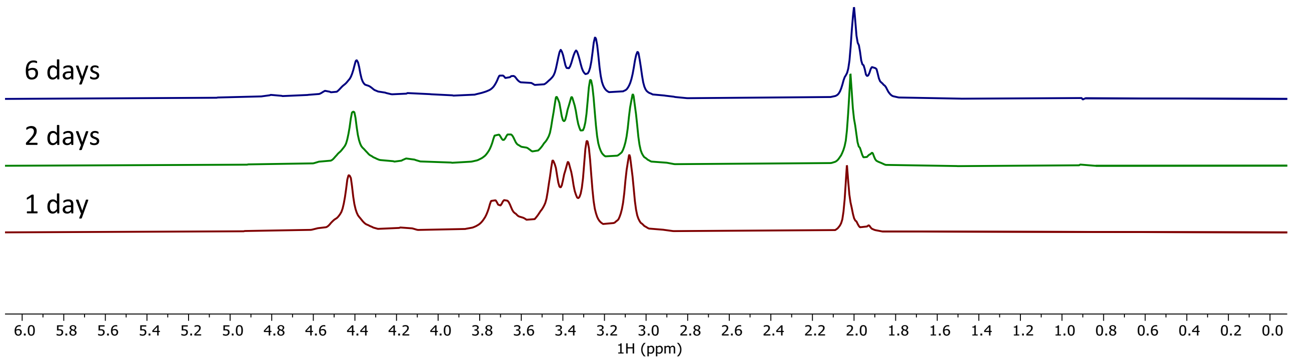

Fig. S31 Expanded diffusion-edited ¹H NMR spectra for FD-CNC samples, acetylated in system "liquid-solid" at 80 °C for 1, 2 and 6 days.

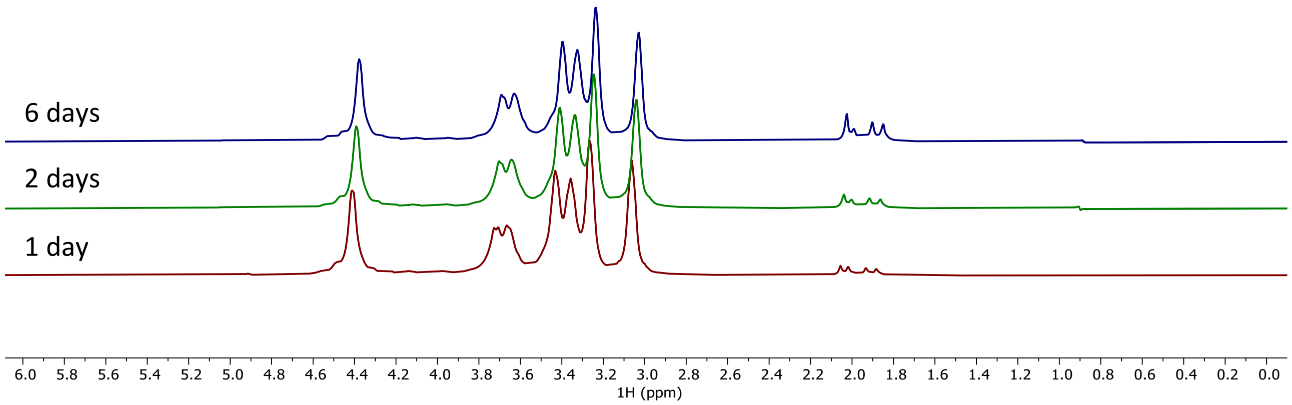

Fig. S32 Expanded diffusion-edited ¹H NMR spectra for FD-CNC samples, acetylated in system "liquid-solid" in the presence of DABCO at ambient temperature for 1, 2 and 6 days.

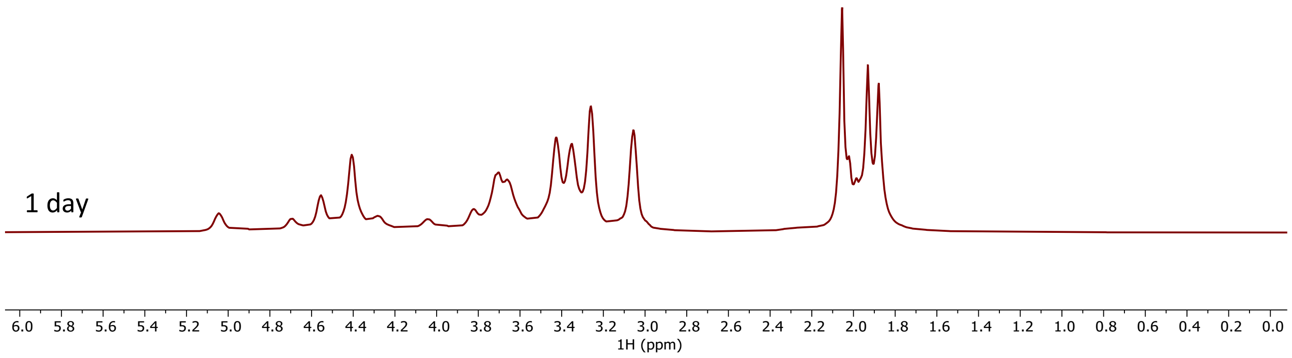

Fig. S33 Expanded diffusion-edited ^{1}H NMR spectra for FD-CNC samples, acetylated in system "liquid-solid" in the presence of DABCO at 80 °C for 1 day.

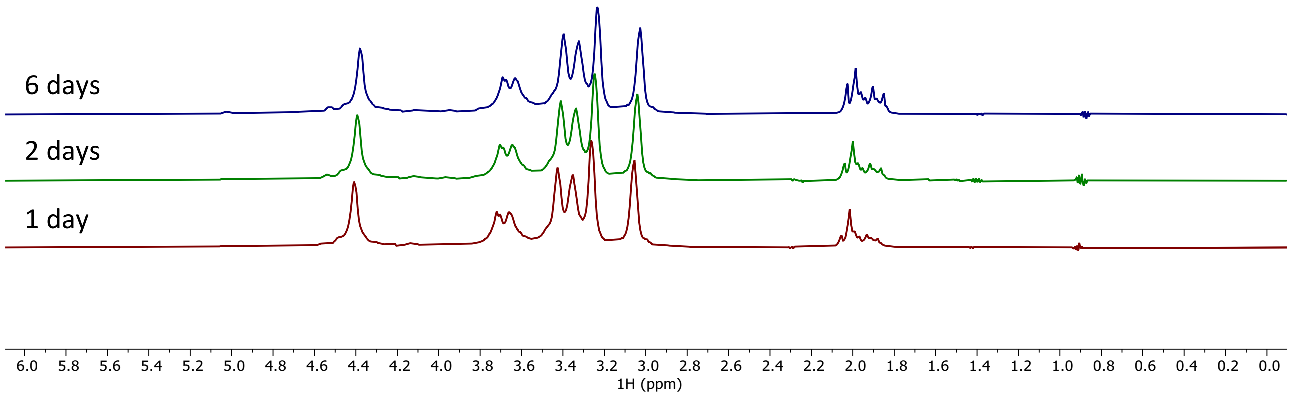

Fig. S34 Expanded diffusion-edited ^{1}H NMR spectra for FD-CNC samples, acetylated in system "liquid-solid" in the presence of pyridine at ambient temperature for 1, 2 and 6 days.

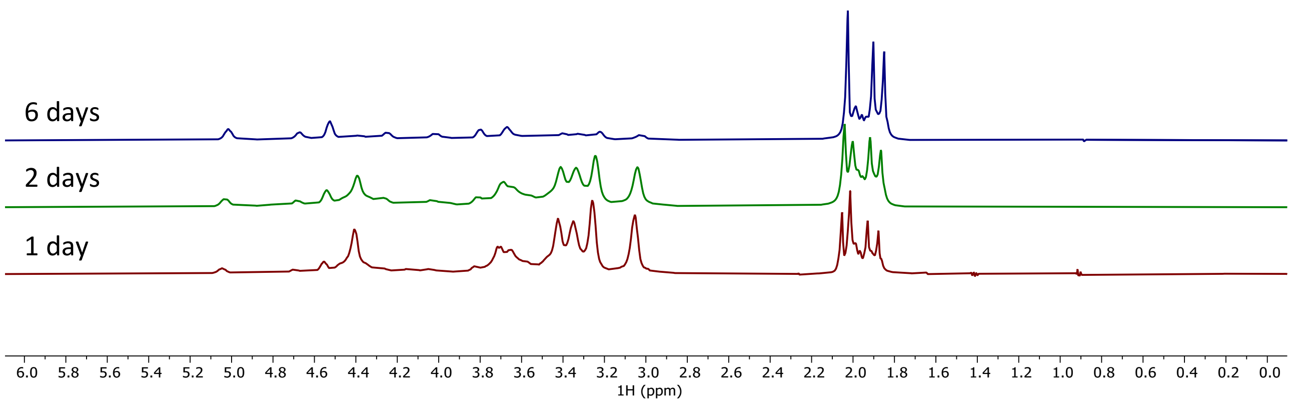

Fig. S35 Expanded diffusion-edited ^{1}H NMR spectra for FD-CNC samples, acetylated in system "liquid-solid" in the presence of pyridine at 80 °C for 1, 2 and 6 days.

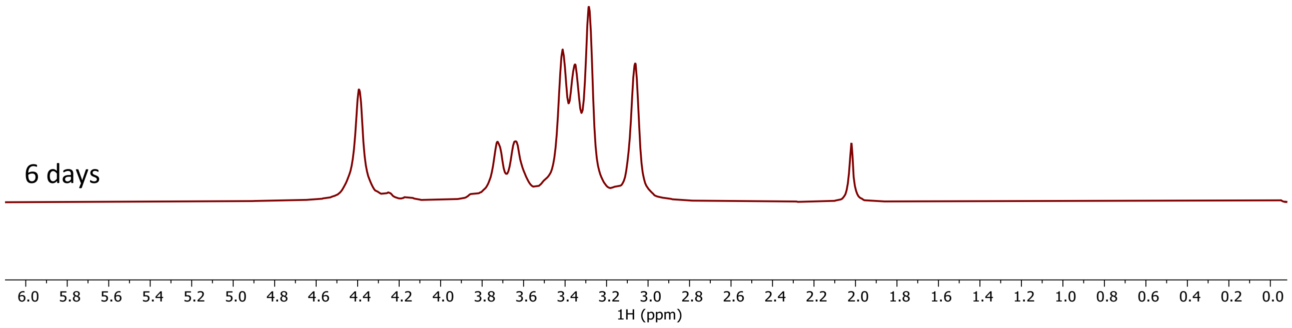

Fig. S36 Expanded diffusion-edited ^{1}H NMR spectra for beech BH-S-P (bleached hardwood sulphite pulp), acetylated in system “gas-solid” at ambient temperature for 6 days.

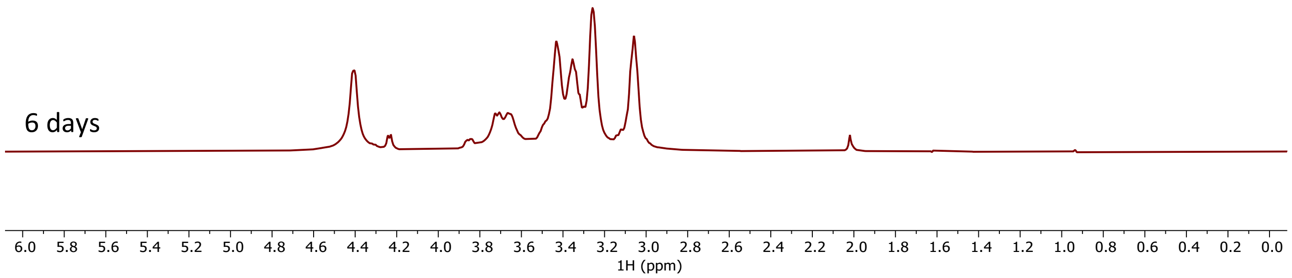

Fig. S37 Expanded diffusion-edited ^{1}H NMR spectra for Enocell BH-PHK-P (bleached hardwood pre-hydrolysis kraft pulp), acetylated in system “gas-solid” at ambient temperature for 6 days.

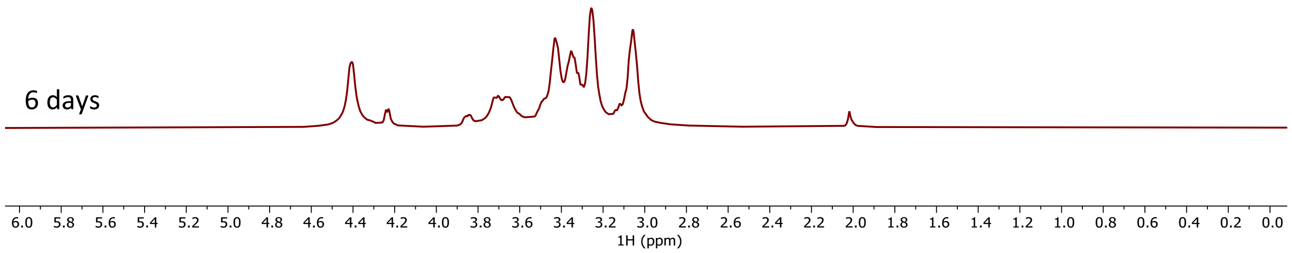

Fig. S38 Expanded diffusion-edited ^{1}H NMR spectra for never-dried Enocell ND-BH-PHK-P (bleached hardwood pre-hydrolysis kraft pulp), acetylated in system "gas-solid" at ambient temperature for 6 days.

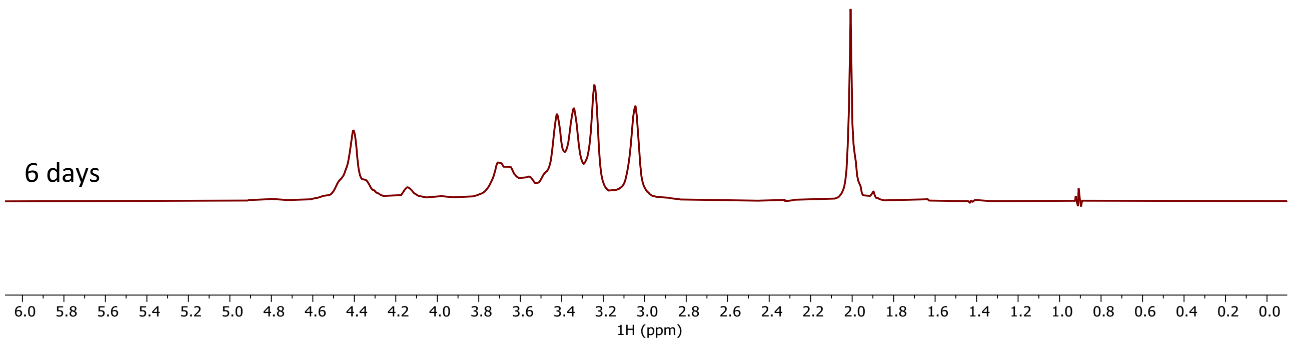

Fig. S39 Expanded diffusion-edited ^{1}H NMR spectra for cellulose nanocrystal aerogel, acetylated in system "gas-solid" at ambient temperature for 6 days.

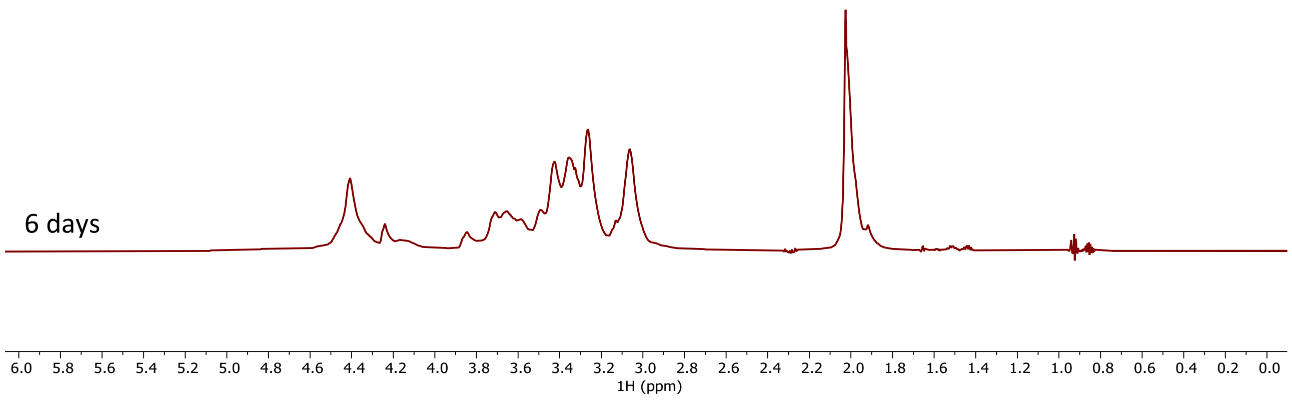

Fig. S40 Expanded diffusion-edited ^{1}H NMR spectra for Ioncell-F, acetylated in system "gas-solid" at 80 °C for 6 days.

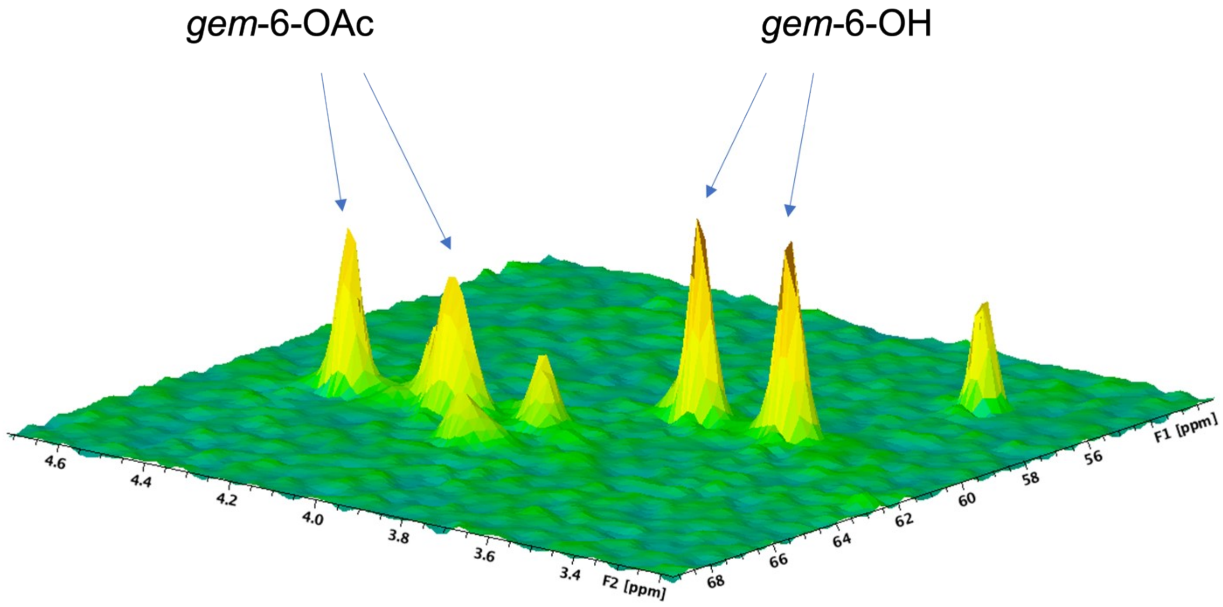

Fig. S41 HSQC spectrum of the *gem*-6 AGU region for 32 d acetylated FD CNCs, showing approximate 1:1 ratio of *gem*-6-OH and *gem*-6-OAc peaks.

4. AFM

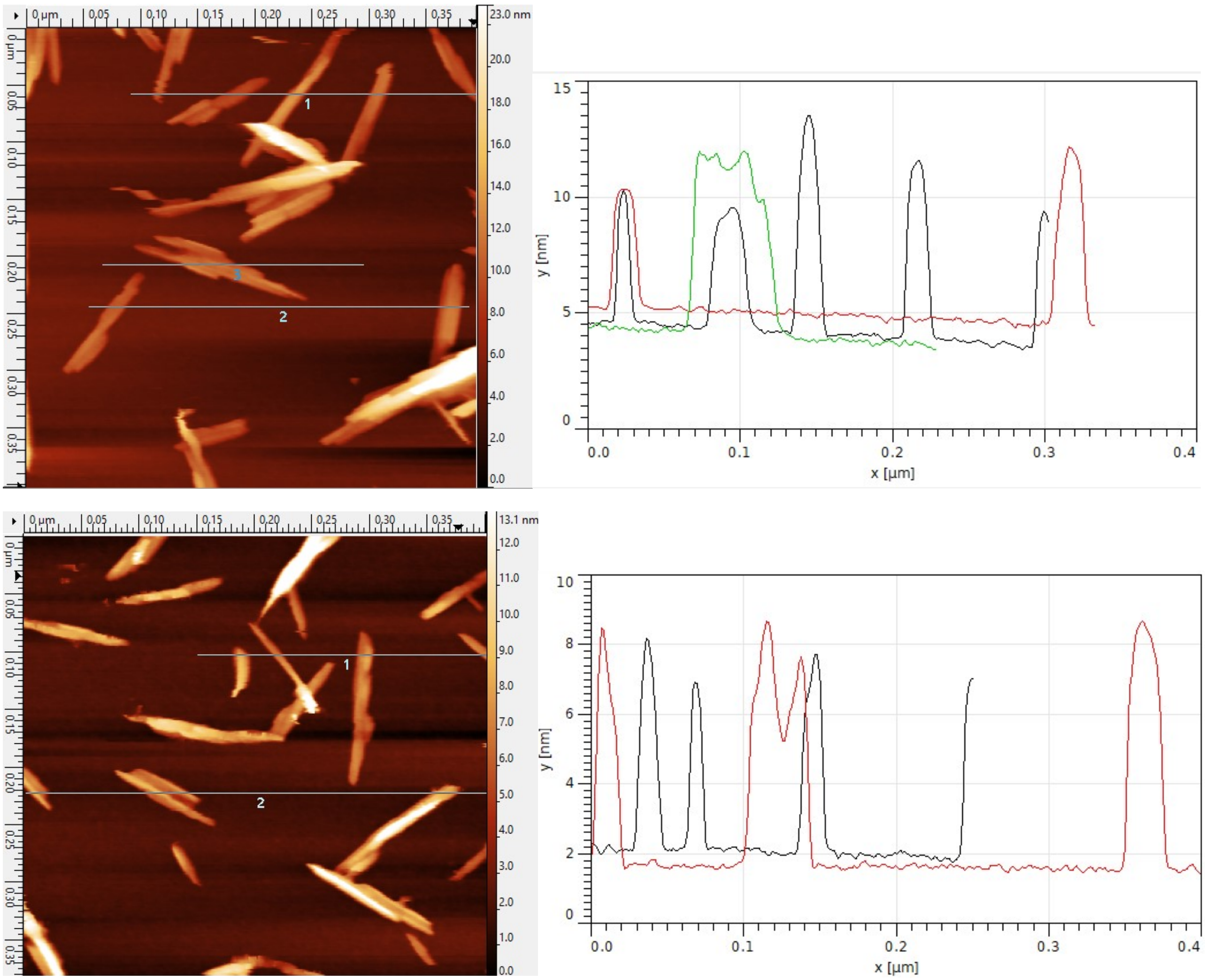

Fig. S42 AFM images of unsubstituted commercial FD-CNCs.

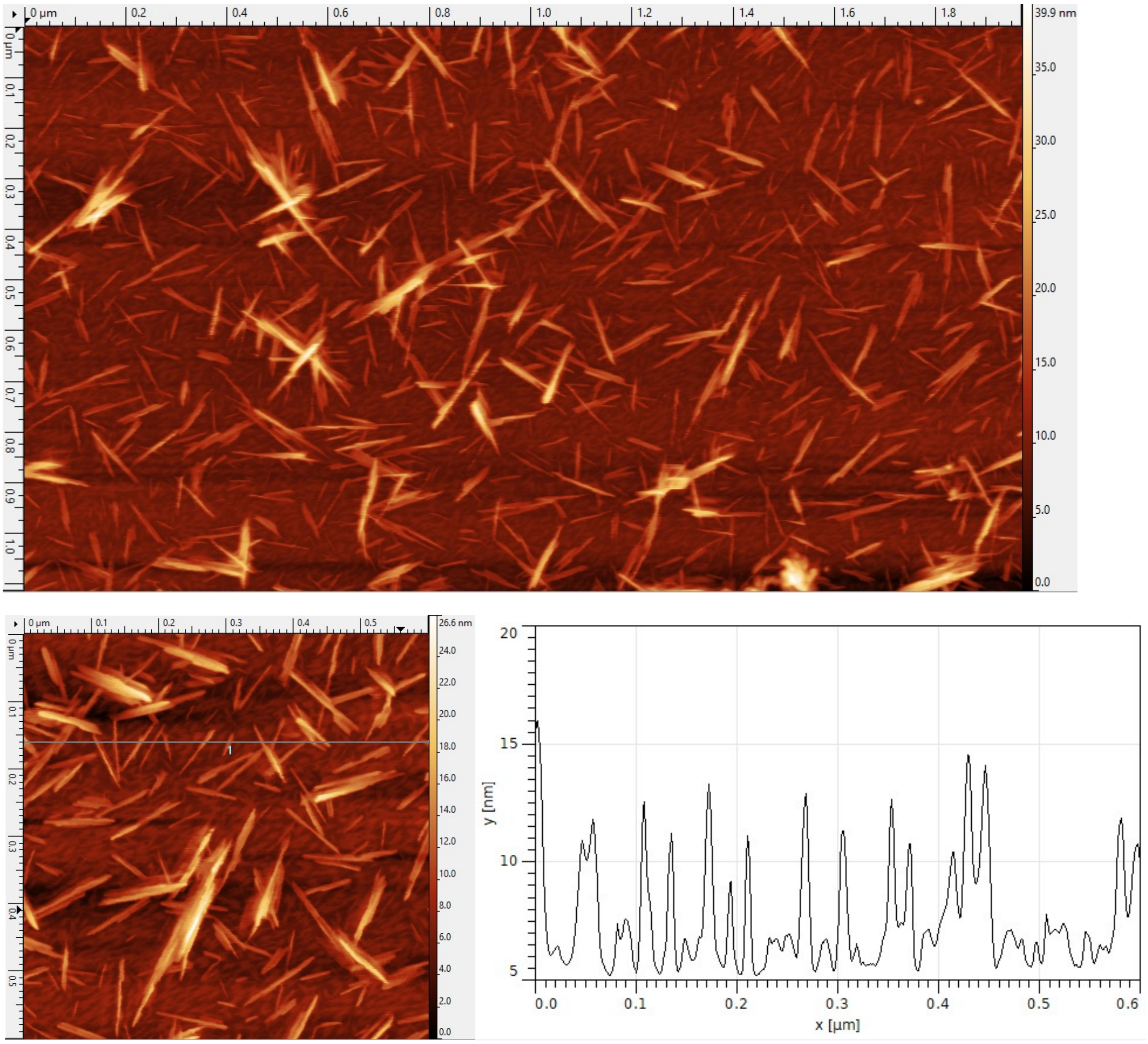

Fig. S43 Further AFM images of commercial FD-CNCs, acetylated in the system “gas-solid” for 32 days at ambient temperature, with determined sizes.

5. ATR-IR

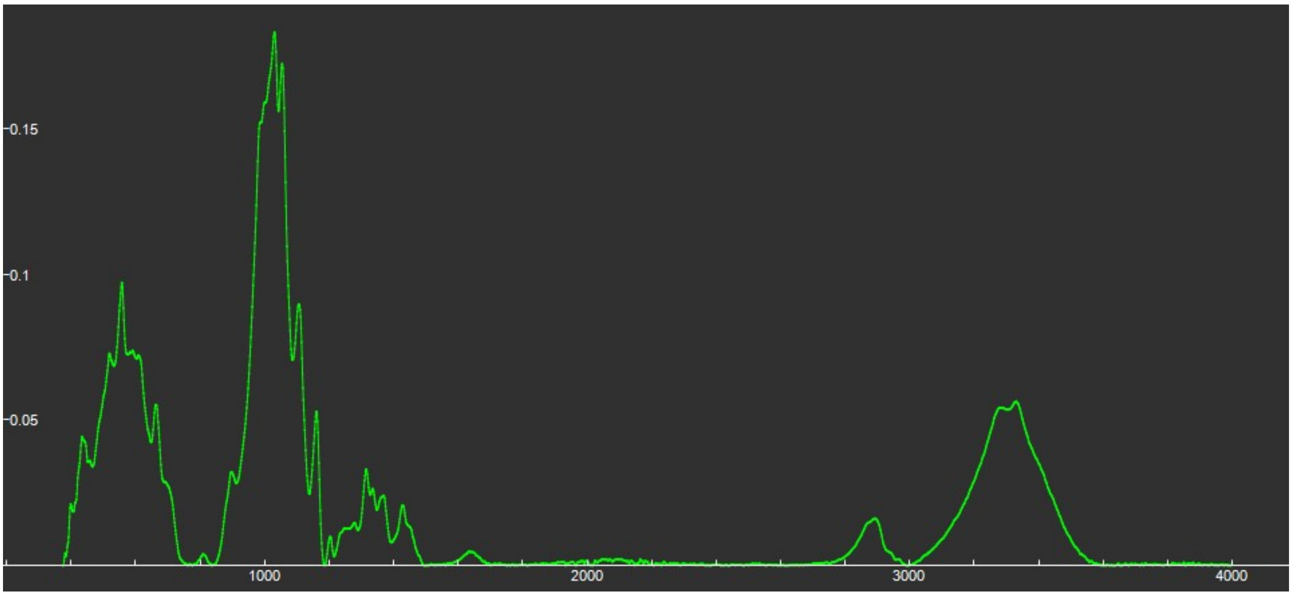

Figure S44 ATR-IR spectrum of commercial SD-CNC (spray-dried cellulose nanocrystals) sample, acetylated in system “gas-solid” for 6 days at ambient temperature.

6. XPS

Spectra were recorded on a Kratos Axis Supra X-ray Photoelectron Spectrometer employing a monochromated Al K_α (hv = 1486.7 eV, 8 mA) X-ray source, hybrid (magnetic/electrostatic) optics with a slot aperture, hemispherical analyser, multichannel plate and delay line detector (DLD) with a take-off angle of 90°. The analyser was operated in fixed analyser transmission (FAT) mode with survey scans taken with a pass energy of 160 eV and high-resolution scans with a pass energy of 20 eV. The resulting spectra were processed using CasaXPS software. Binding energy was referenced to aliphatic carbon at 285.0 eV. High resolution spectra were fitted using the "LA(α,m)" lineshape for symmetric peaks corresponding to a numerical convolution of Lorentzian functions (with exponent α) with a Gaussian (width m). Details of this line shape function is available in the CasaXPS documentation online.

Empirical relative sensitivity factors supplied by Kratos Analytical (Manchester, UK) were used for quantification. Use of these relative sensitivity factors does not account for any attenuation due to overlayers or other surface contamination and assumes a uniform depth distribution of elements within the information depth of the sample. Matrix effects are also discounted. Quoted standard deviations result from averages of three measurements per sample.

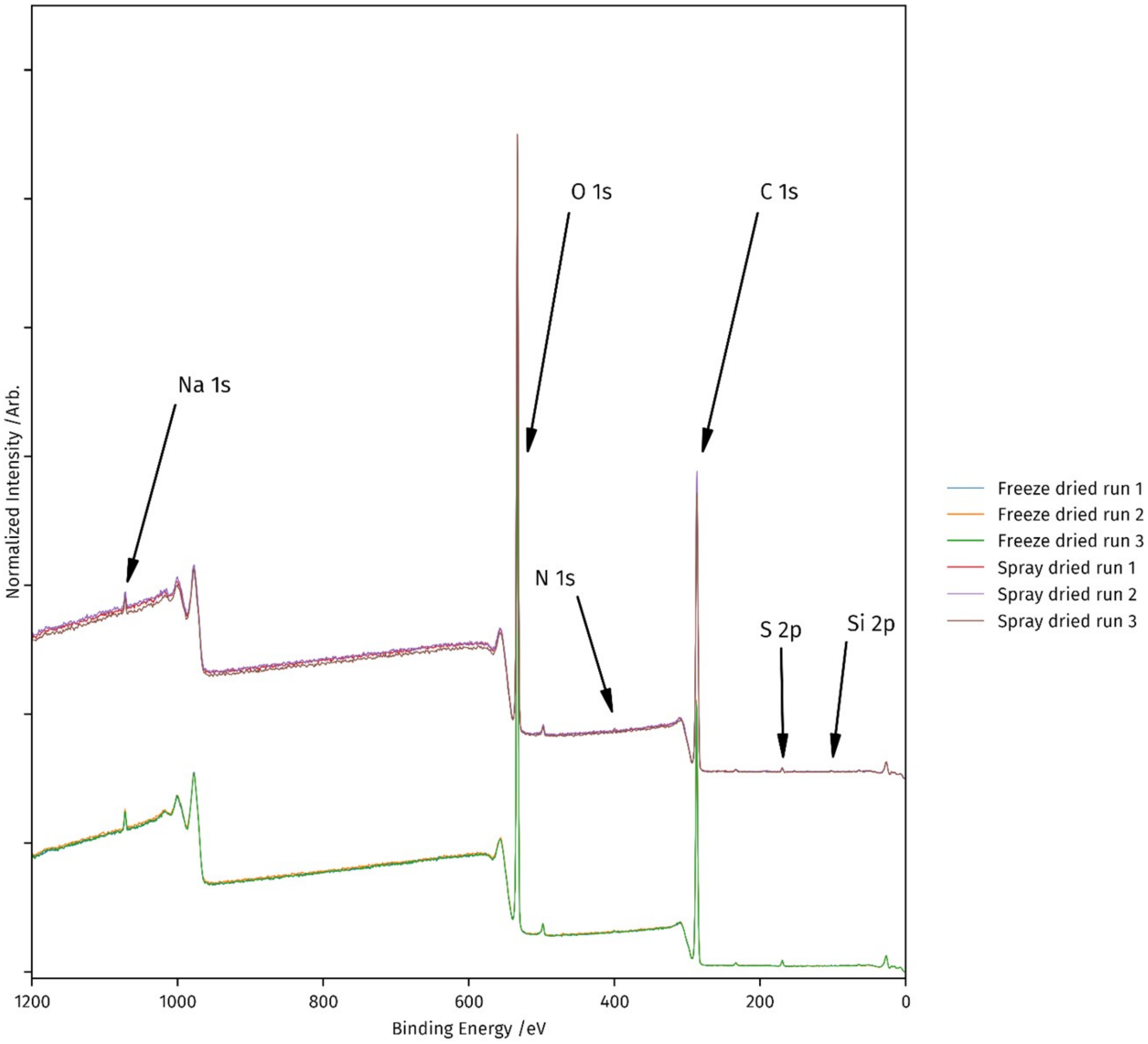

Figure S45 XPS wide scan spectra of FD-CNCs and SD-CNCs.

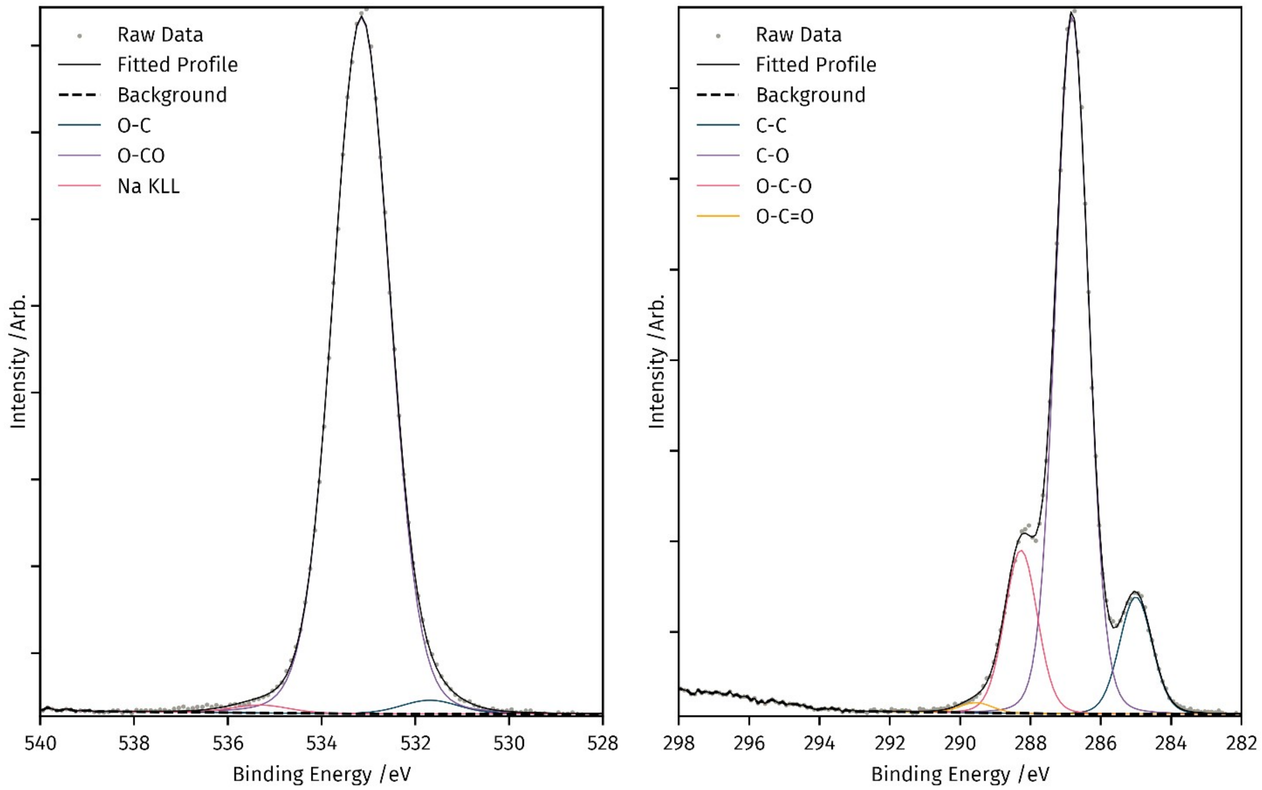

Figure S46 Peak fitting XPS of commercial FD-CNCs.

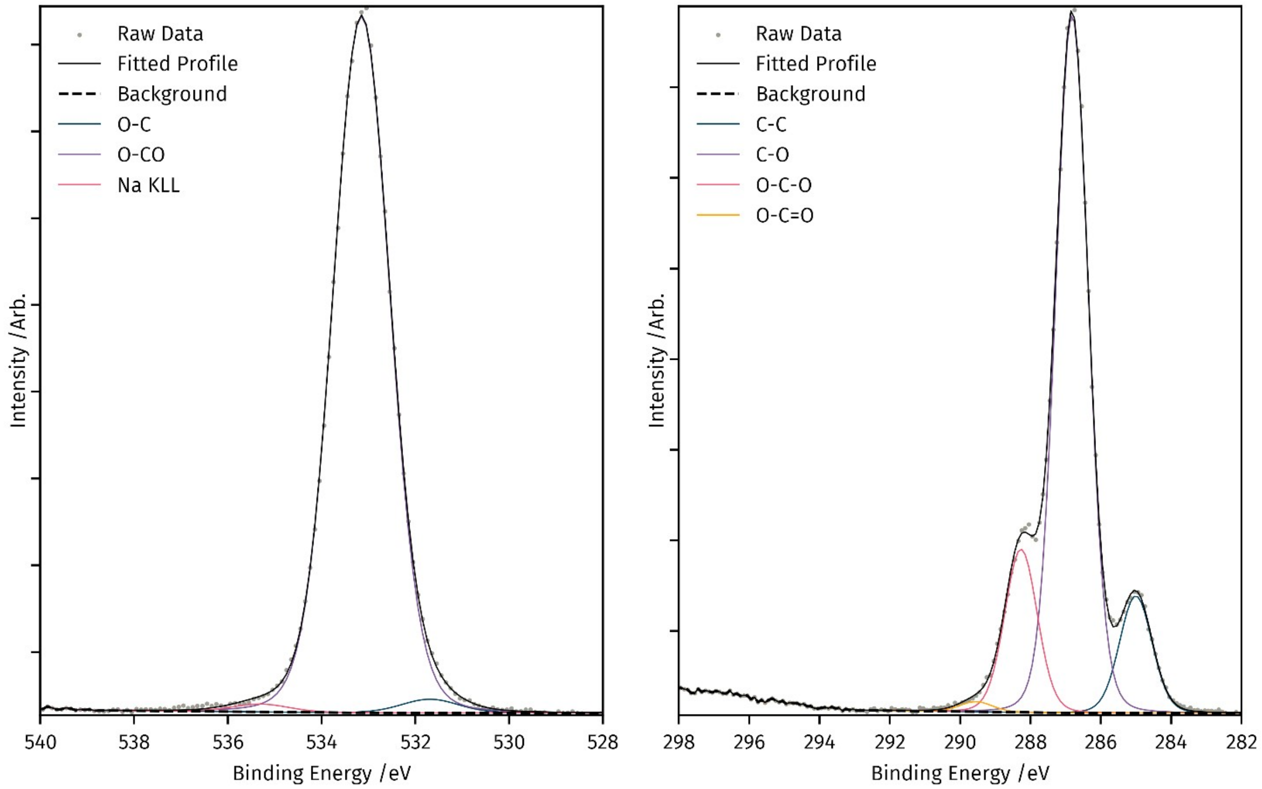

Figure S47 Peak fitting XPS of SD-CNCs.

7. Computational Experimental

The transition states (TS) for acetylation of 2,3 and 6-OH acetylation were located through relaxed potential energy surface (rPES) scans for low energy acetate orientations (corresponding dihedral angles between AGU and acetate) in positions 6, 3 and 2 on a cellulose I_β surface fragment. This was followed by rPES bond-length scans for the low energy acetate conformers, for the acetylation-deacetylation reaction coordinates. Transition states searches (OptTS) with final analytical frequency calculations (Freq) were then performed from the rough rPES transition states. The Gibbs energies of the transition states only were compared, against the lowest transition state energy. Full reaction profiling was not performed as there is a significant contribution from basis set superposition error (BSSE) using the current basis set (def2-SVP). Rather, the BSSE for the transition states was assumed to be approximately the same, allowing for direct comparison of the transition state energies. This is not possible with starting, intermediate and ending reaction geometries which have fully separated species, in some cases, leading to much less basis-set overlap.

The ORCA 4 package (Neese 2018) was used with the B86 GGA functional (Becke 1988; Perdew 1986), def2-SVP basis set, Grimme's D3 dispersion correction (Grimme 2010) with Becke-Johnson dampening (Grimme 2011) and the resolution-of-identity (RI) approximation (Eichkorn et al. 1995; Eichkorn et al. 1997).

The initial cellulose Ib fibril (hexagonal 36 chain) with a polymer length of 4 glucose units was generated using the 'Cellulose-Builder' (Gomes et al. 2012) web interface (http://cces-sw.iqm.unicamp.br/cces/admin/cellulose/view). This was then edited in Avogadro 1.2 (Hanwell et al. 2012) to remove all polymer chains except for a (110) surface section of 3 stacked polymer chains with a length of 4 AGUs each (Figure S48).

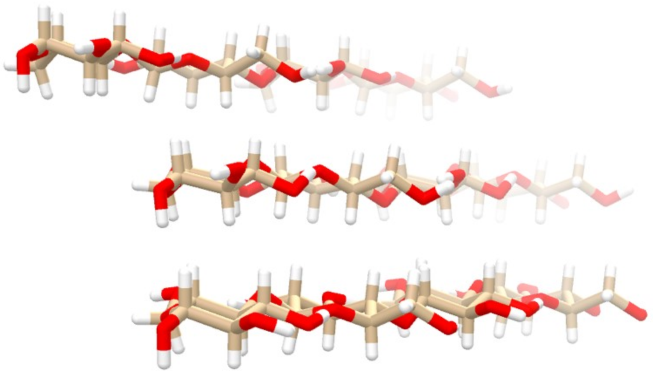

Figure S48. Initial cellulose Ib surface fragment used for the calculations.

Hydroxyl groups were added to the reducing ends, as these are missing in the Cellulose-Builder outputs. For the rPES scans, an acetate was added to the relevant OH (2,3 or 6) of a central AGU. A rPES scans for dihedral angles corresponding to acetate group rotation were completed at the RI-BP86/def2-SVP-D3(BJ) level throughout the full 360 ^{o}; except in the case of 3-OAc where the calculations failed at certain dihedral ranges, due to steric interactions giving highly distorted geometries (Figure 3, main text); constraints were used on all atoms except the 6-OAc, all oxygens, all 1,2,3,4-hydrogens attached to OHs and the 6-CH_2 positions attached to 6-OH and 6-OAc (Figure S49).

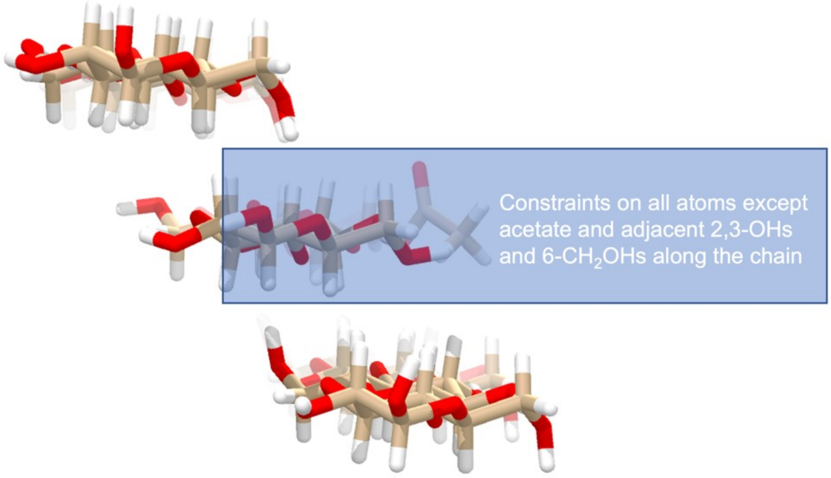

Figure S49. Surface constraints applied to all but a few key atoms.

This prevented movement of the AGUs away from the geometry found in the typical cellulose Iβ crystalline structure but allowed for enough freedom for formation and breakage of H-bonds, necessary for the stabilization of the conformers. The dihedral rPES scans and final TS geometries are shown in Figure 3 (main text). The final TS geometries (and expanded images) are given below:

6-OAc Transition State

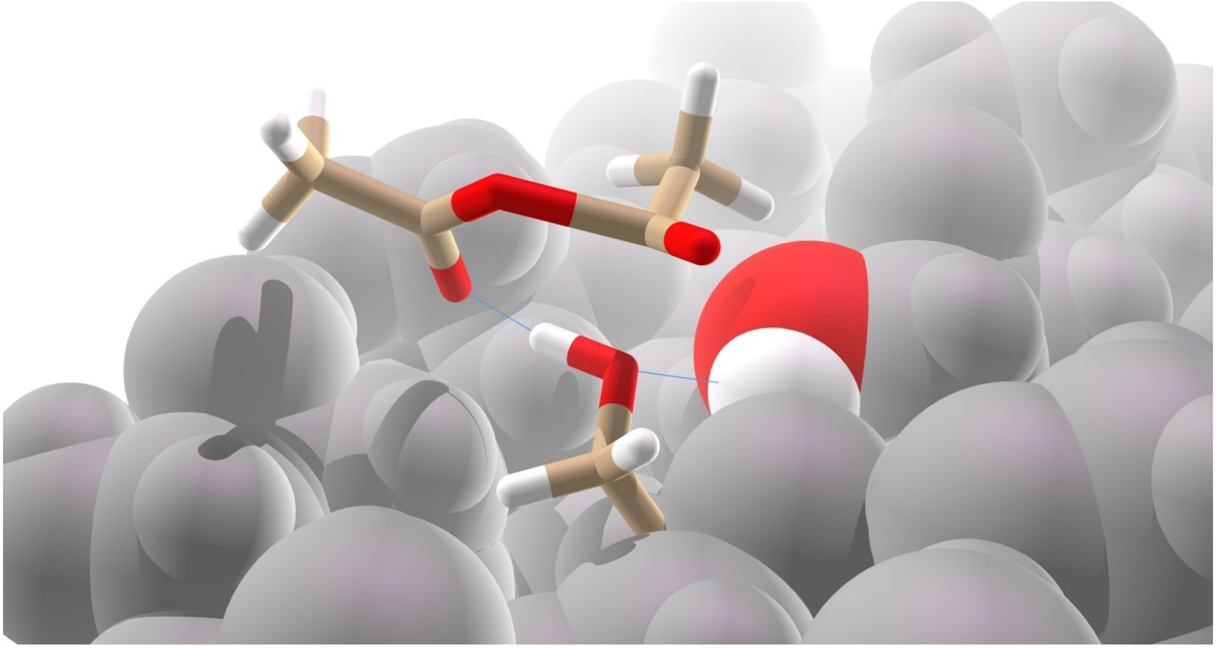

274

Coordinates from ORCA-job orca

C 7.90039044001538 -10.66124842721790 11.07556328989531

H	8.97445986480863	-10.52002752057295	11.21974120013024
C	7.62913078188771	-11.81386210043214	10.10055856482294
H	6.56944984030665	-12.08256984441088	10.13819089822714
C	7.95046949085741	-11.42069948563143	8.66497026072441
H	8.99563992654721	-11.32160004913668	8.48757021994408
C	7.29623996152676	-10.09602973983413	8.29952974771731
H	6.22152003204647	-10.27531995663303	8.21039002988259
C	7.58431034249035	-9.02272187878265	9.36462185133117
H	8.63722907623095	-8.72595820139459	9.31872788676627
C	6.70186010788031	-7.79153005909256	9.12775999349707
H	5.68848006837855	-8.10951998109675	8.85909000023768
H	6.62105002614974	-7.21328997888911	10.05390000016280
O	8.44542221798666	-12.93017891206733	10.41269299113763
O	7.42730998509143	-12.41800999682095	7.79056010034490
O	7.84915003906013	-9.66308008069588	7.06119005347281
O	7.25683979860757	-9.46927030867960	10.67067151865503
O	7.18522006297732	-7.01402009550220	8.03319010349695
H	8.29847782873705	-13.20366096040777	11.34720879140807
H	7.81008997869157	-13.27389001123865	8.06742999971568
H	7.96054008948343	-6.49014008840540	8.31658997706581
C	10.73083990959179	-6.28131996888300	8.20352989106023
H	11.78829007138994	-6.01324019261633	8.32819013300684
C	10.63418065576774	-7.43820084892041	7.20100118292068
H	9.65028935684239	-7.91178918262007	7.23955899413511
C	11.01473020240034	-7.04003020649336	5.79800992056711
H	12.10807992751784	-6.96156995066652	5.72811997520181
C	10.33723996281577	-5.74208998033905	5.40265998629450
H	9.27552996145017	-5.94648995763190	5.22223999439172
C	10.48266001367467	-4.68880999385830	6.51467000188875
H	11.53821000387078	-4.41131999497670	6.62183999271284
C	9.67509999891796	-3.43549999323389	6.20570000083466

H	8.64152999906649	-3.70902999481315	5.96219000179491
H	9.64252000143153	-2.80331999696460	7.09776999701155
O	11.57886260165005	-8.46198095377861	7.57786027911520
O	10.59202942749688	-8.02050474220767	4.84682889638295
O	10.91682999403732	-5.18643998993909	4.22676999706872
O	9.97397000965783	-5.17952005684137	7.75660004659364
O	10.27499000000402	-2.66654999545005	5.16963999704941
H	11.12720742859208	-9.04212824612867	8.21736128329204
H	10.97612535356127	-8.87003842371496	5.14030540570000
H	10.51030999987433	-3.27371999962929	4.43795000152149
C	14.16594999993112	-2.03078000002908	10.76272999404105
H	15.24739999776310	-1.88313999681746	10.88309999907172
C	13.90064998981247	-3.17872999472062	9.78659999556626
H	12.83809999751667	-3.43284002358080	9.79156001955104
C	14.30409999650696	-2.76752000100174	8.37425999858951
H	15.39094999779209	-2.63572999502779	8.30743999938598
C	13.57823999944069	-1.46880000098514	7.99651999918876
H	12.50256999999061	-1.67634999598803	7.91509000252218
C	13.83835000243910	-0.38901999806333	9.06013000155027
H	14.89160999874252	-0.08078999679562	9.01449000172811
C	12.94083999853824	0.82979000142608	8.80774000052594
H	11.92592999897301	0.50807000165464	8.54674000134894
H	12.85178999967227	1.42023000202534	9.72525999931934
O	14.67815999127872	-4.31636001256475	10.13332998643706
O	13.90542999812998	-3.77165999264412	7.43968999544229
O	14.05236999892640	-0.92857999768575	6.76771999956696
O	13.51611000214933	-0.84260999927191	10.36611999827494
O	13.48602999780165	1.66791999866355	7.79477999756717
H	14.48854002889381	-4.57883996502351	11.06431001344733
H	14.34537000752135	-4.60305999036567	7.70359999580296
C	10.74181986507712	-5.66798005506578	13.42884016341585

H	9.70931000586714	-5.96223994048378	13.65236001363791
C	10.73572001522521	-4.49823998501424	12.44055001461739
H	11.74290004024887	-4.08752999589118	12.34133997967834
C	10.21460992344093	-4.91854009993120	11.06631005408021
H	9.12596999321718	-5.03246001661437	11.10266001623651
C	10.87959012444643	-6.21941963279512	10.60507044871092
H	11.93101001821342	-6.02749013291589	10.36267009177377
C	10.81745440755735	-7.24059375144457	11.75835453164203
H	9.77882328749852	-7.53764102854507	11.94706219117468
C	11.55958216204639	-8.54220860368734	11.42159626013382
H	11.08494524934300	-9.04423051908436	10.55213503852124
H	12.62294839446719	-8.32680265344922	11.16937529593595
O	9.85455000294852	-3.50735998952421	12.94642998598665
O	10.54179000264639	-3.85600001967431	10.17418000159076
O	10.23358997819311	-6.74926004914500	9.44571003275781
O	11.46015022309569	-6.76634996151464	12.91064999978188
O	11.53815621176500	-9.37465942831486	12.57233891390042
H	10.21401998555678	-3.20407003240529	13.80819997869548
H	10.11997998686765	-4.02870001285157	9.30723999784382
C	13.23544431945541	-10.11537367511121	12.96330479083579
C	7.61209984229365	-9.98883012489934	16.29094998302704
H	6.54899999388169	-10.13931001727294	16.52098996074678
C	7.77547970970231	-8.83691882382857	15.29657001601507
H	8.83345051044555	-8.58558134059514	15.19175958648969
C	7.23179020156929	-9.24201957454501	13.93300081668969
H	6.14418986263945	-9.37941988670889	13.97476973280823
C	7.92203925928538	-10.53770123211214	13.47993825381773
H	8.98932979867072	-10.33279164686770	13.31524428039592
C	7.76509006382960	-11.62194020061092	14.55934986832265
H	6.71222996226047	-11.93144986324134	14.61973960732817
C	8.60173967893832	-12.90737547401858	14.21589369416846

H	9.62259154364136	-12.61519315798053	13.95773576932118
H	8.65203986573759	-13.53043005026579	15.11855004669869
O	7.02923987307437	-7.72249991867614	15.75850003003219
O	7.55198049125844	-8.17407193245558	13.04734973812018
O	7.33254995767708	-11.05691034132446	12.30074166959304
O	8.22629010889263	-11.17898993793920	15.81894972183473
O	8.07155001759195	-13.63131035717965	13.11177960317588
H	7.33380002650653	-7.47497002557993	16.66168999176971
H	7.23341143085799	-8.41087355455221	12.15129215896945
H	7.18012006311941	-13.96132004583526	13.33041004358940
C	13.85195000447716	-1.35451000222504	15.97987999826862
H	12.79221000114060	-1.49929000228499	16.21688000180953
C	14.02341999958929	-0.21980999903554	14.96999000160069
H	15.08933999926094	0.01930999902430	14.87169000110128
C	13.46320000244288	-0.63077999895677	13.61747000161159
H	12.37592000270768	-0.76811999993118	13.66515000053711
C	14.16461000368867	-1.91435999347286	13.16379001119744
H	15.23000000036546	-1.70354000072407	13.00564000157920
C	14.00735999560423	-2.99404000542535	14.24710980413681
H	12.95770002033242	-3.29497996814914	14.30774020798021
C	14.82849999532829	-4.28855999966964	13.89237000152220
H	15.83230000653730	-4.00375001169953	13.56397000114898
H	14.93556999589241	-4.88343000290118	14.80797999982891
O	13.31689999848544	0.89725999965297	15.49407000022329
O	13.74135000011188	0.47305000164395	12.76228999912065
O	13.60428999673514	-2.44531000334766	11.98228999524364
O	14.47156999568700	-2.53939001128239	15.50435001124066
O	14.22773992252307	-5.03986986316974	12.84536002400907
H	13.50487999932728	0.23419999967310	11.83960999884836
H	13.45737008945930	-5.53840016673474	13.18286002759095
C	8.46032999518896	-10.67053998902417	21.43805998414020

H	9.53873997677899	-10.52504000030139	21.56899000503710
C	8.20077998251938	-11.82003001442338	20.46482997151800
H	7.13354996738559	-12.07778998663908	20.47344999767095
C	8.60495004988675	-11.40791003364017	19.05466993892260
H	9.69103002389142	-11.26863002944452	18.98624007462662
C	7.86335000032003	-10.11610996404179	18.67979999536280
H	6.78663001739414	-10.32400002947797	18.63136997818432
C	8.13014999195363	-9.03503995491921	19.73850999953626
H	9.18043000909983	-8.73337005425397	19.69224999262288
C	7.23909000036556	-7.80882999921960	19.49319001184784
H	6.22495000208369	-8.13162999881809	19.23497000057534
H	7.16302999917037	-7.21958999861084	20.41329000104045
O	9.01260997614376	-12.91971999803672	20.83562999284228
O	8.20787037426305	-12.48098996035162	18.21102963851546
O	8.29851998295715	-9.58359995155728	17.44526993593621
O	7.80041999561665	-9.48611000783672	21.03708000420225
O	7.71627995740204	-6.99788995296340	18.42143000919124
H	8.78562000569233	-13.20970999224260	21.74784000076451
H	8.41467957383932	-12.24200005086210	17.28413048693296
H	8.56192998051233	-6.58096997644965	18.68055003868315
C	11.28071991760698	-6.32069998315102	18.57163999580129
H	12.33450002629571	-6.04202000301353	18.69685996934184
C	11.19260010253408	-7.50669014427604	17.60410991755325
H	10.18507996844965	-7.92877002466351	17.61190004582075
C	11.57188996315714	-7.08620991648934	16.18976996406782
H	12.65947993361299	-6.98118979224412	16.11484003524121
C	10.86413000835283	-5.78111006807822	15.79697000318059
H	9.79490992193274	-5.98382995097812	15.66605999398747
C	11.02639844726038	-4.69825429599384	16.87548942585076
H	12.10022679146881	-4.41374248199549	16.99206543407445
C	10.20489999646851	-3.45874999936606	16.57668999824293

H	9.16972999967783	-3.73213000118409	16.34686999981615
H	10.18701999970891	-2.81164999720984	17.45898000021272
O	12.11255936519912	-8.49356870416471	18.04413180655593
O	11.16796558654108	-8.14973952021681	15.32605576907839
O	11.38843000617410	-5.24483999753500	14.59601000160917
O	10.52279001273209	-5.20531999481455	18.12722003290268
O	10.78659000108910	-2.72706999486052	15.49029996608176
H	11.80272846789645	-8.79285913912511	18.93388144631498
H	11.39886654638560	-7.91858738548223	14.39353963578955
H	10.40300000434471	-1.82817999683042	15.48571001610137
C	14.70292000057464	-2.04541997929593	21.12470998493595
H	15.78404999784411	-1.89528000145512	21.24571999566329
C	14.43772014362038	-3.19374056452247	20.15075020488909
H	13.37450982229359	-3.44499928454640	20.15691976036443
C	14.83804998251486	-2.78413002522374	18.74045999820694
H	15.92382999795181	-2.64450998466401	18.67227000070160
C	14.10051000585433	-1.48814999119131	18.36773999528046
H	13.02266999864588	-1.69428999887755	18.31532000273512
C	14.37131999845569	-0.40777000216308	19.42940999810060
H	15.42551000212514	-0.10007999914183	19.38068000048789
C	13.47999000107569	0.81568999997241	19.17486999812422
H	12.46440999954311	0.50161999825117	18.90531999782059
H	13.38725000193204	1.40205999828858	20.09443000145246
O	15.21741999201828	-4.31428997736710	20.53942999644307
O	14.42800999065207	-3.85275996653942	17.89118998924251
O	14.53942999815227	-0.94926000006590	17.13419999791908
O	14.04497999668093	-0.85963000166850	20.73071000442756
O	14.02563000225861	1.69219000186076	18.19961999922898
H	14.96891999965442	-4.57944000131882	21.45539000739288
H	14.66145001215286	-3.62025002017320	16.96878000205974
C	11.19505000767520	-5.72577997674838	23.73650999256287

C	11.24306003326320	-4.52466003695332	22.78906002710212
H	12.26332999468214	-4.13958001035620	22.70491000275055
C	10.73629796752470	-4.92784814736522	21.41228951851467
H	9.63800396938855	-5.12658746913219	21.49882039617717
C	11.39760729695335	-6.23517814003183	20.96124807268476
H	12.45985000035626	-6.04616006263524	20.71715995836633
C	11.36229001163058	-7.31870996954335	22.05081999373126
H	10.32553998303803	-7.61734000922430	22.24424999481669
C	12.17946141280772	-8.56742952888711	21.69953737161980
H	13.19012016004696	-8.26512935778423	21.34224680370122
H	12.30600893896055	-9.13759235721353	22.64577064622738
O	10.41452000188248	-3.52421000043734	23.37397999521389
O	10.99182345896581	-3.83147948195231	20.54596393296027
O	10.75218999120219	-6.75067002389460	19.79660998325316
O	11.95072999450107	-6.79731003260213	23.22423000527448
O	11.49957066226566	-9.35669657518005	20.71375302033062
H	10.38811000042367	-2.75976000227670	22.76691000248152
H	10.71348003608511	-4.08263012847623	19.63572003105895
H	11.87808477014012	-10.25606938755456	20.73280833094959
C	8.07108999880809	-10.00520000255962	26.61057999779691
C	8.28618999826997	-8.84525000204980	25.63963999920487
H	9.35751000249573	-8.61740000171075	25.55502999823722
C	7.75680999944887	-9.25045000232952	24.27420000092926
H	6.66947000153237	-9.38742999965389	24.31299999828111
C	8.45356999920916	-10.55101000194294	23.83272000307036
H	9.52226000354796	-10.34434000278071	23.67762000007332
C	8.29491000276031	-11.63687000238384	24.91183999970410
H	7.24349999970539	-11.95236999944796	24.96817999899336
C	9.14283000075680	-12.91639000064812	24.58086000226622
H	10.14946999790546	-12.61995999938038	24.27429999917769
H	9.23394000148190	-13.50288000079451	25.50422000039181

O	7.56584999982056	-7.71060000090585	26.09849000067766
O	8.08251999959100	-8.18646999890201	23.38721999861744
O	7.88632999475654	-11.07628999512252	22.65309000250201
O	8.74341999797957	-11.17160999757058	26.16872999950245
O	8.58574000164021	-13.68411999776952	23.52078000203332
H	7.79594000179337	-7.57251000181618	27.03925999800639
H	7.78294999938097	-8.43046000114007	22.48607000237897
H	7.70656999906813	-14.01793999895065	23.77920999945588
C	14.30712999874638	-1.37641000105031	26.30897000236700
C	14.52565999766043	-0.22527999815866	25.32616999940957
H	15.59877000211284	-0.00658000105769	25.24429000250761
C	13.99108000121151	-0.63518999837604	23.96333999788173
H	12.90381999927916	-0.77400000122786	24.00331000127653
C	14.69823999968728	-1.92274000165586	23.51863000267840
H	15.76486000023639	-1.71307000093789	23.36574000191139
C	14.53814999800487	-3.00192000735117	24.59842999773159
H	13.48581999937546	-3.29841999206409	24.65521999934203
C	15.34043000181083	-4.30661000221920	24.24815999921428
H	16.32394000181586	-4.03994999746776	23.85218999769119
H	15.49383999854771	-4.85794000261365	25.18421000040710
O	13.82977000258400	0.91618000033445	25.80486999923980
O	14.27908999915352	0.46787000198790	23.10999000123985
O	14.14015999481785	-2.45972999270845	22.33900000764501
O	14.99351000205204	-2.53444999991101	25.84819999748986
O	14.67982000240154	-5.09791000210884	23.26733999754266
H	14.05466000245172	0.22899999752673	22.18543999971751
H	13.90180000083464	-5.54086000156768	23.66392999895318
H	13.24032999856288	-1.56201000082051	26.49541999829477
O	14.82745999911937	-1.07992999797981	27.58849000084254
H	7.00544999736776	-10.19115000002798	26.80322000157675
O	8.60619000184341	-9.66466000120637	27.88128999995996

H	9.57407999772235	-9.79384000029849	27.85544000014093
H	15.77832999966683	-0.87643999850444	27.50703000129943
H	10.16269999940069	-6.03049999943917	23.94661000800429
O	11.86121999785555	-5.39605999854121	24.94860999930295
H	11.40628999797057	-4.62450000174380	25.34156999849899
H	7.42001004327828	-8.82184008703825	6.80452013021436
H	10.85076930797601	-5.87426390425053	3.53540664327763
H	13.82374609266498	-1.57472211776881	6.07130667473890
H	13.65554778915125	1.06663194943027	7.03696955949250
H	13.87248478467456	1.30417073974413	17.30761072354009
H	13.78832656345432	1.51111536150297	25.02743656926699
H	13.32346506104373	1.57004194253939	14.78202912512313
C	12.95249507851196	-10.65925913300467	14.32273468426927
O	14.08845891709015	-9.43398191753144	12.50668881007315
H	12.72137940385980	-9.81409026287713	15.00331560864595
H	13.85253228967729	-11.20308616674428	14.66927234493131
H	12.07944082077780	-11.33352072655847	14.31967055765638
H	11.67403828463259	-14.26999497585592	11.77435251828867
O	10.75321761309695	-11.62661809993189	12.15432552600566
H	11.05268038364970	-10.34033271115861	12.40480198522904
C	11.75087319073501	-13.47018193398041	11.00842487200555
C	11.83627452429667	-12.14190820223752	11.71624911410498
O	12.98779998814378	-11.59542840803211	11.85221140093042
H	12.64913872044643	-13.65537018897817	10.39267432131320
H	10.81697528077324	-13.49191956862691	10.41328796914033

2-OAc Transition State

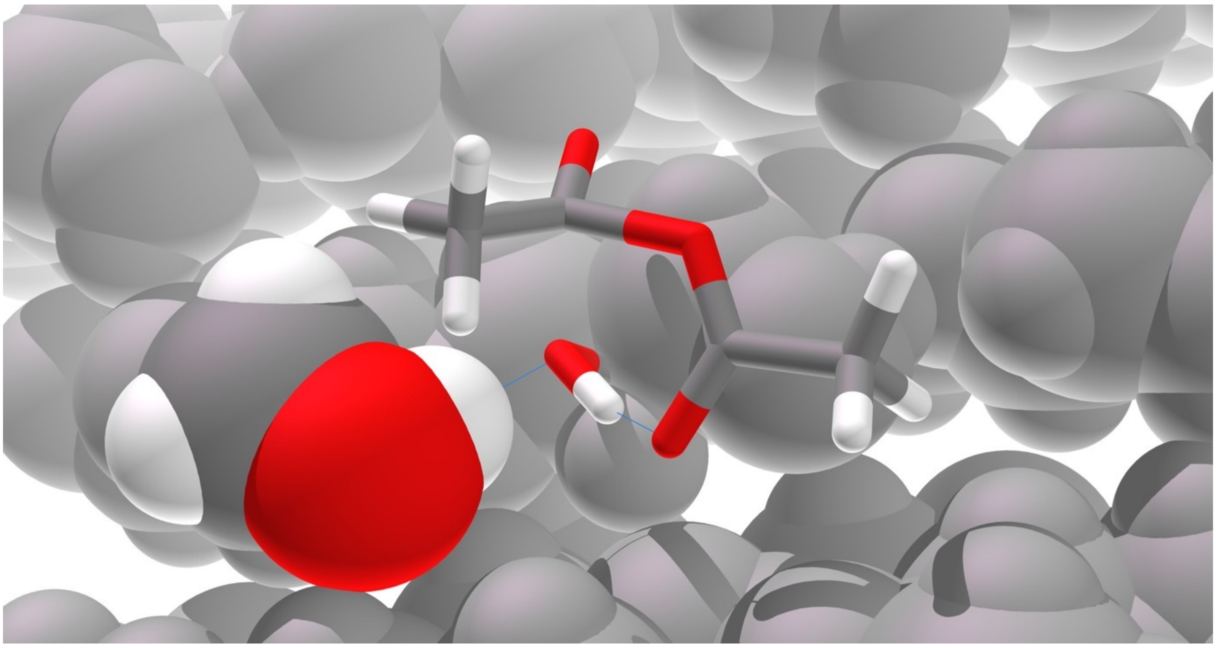

274

Coordinates from ORCA-job orca

C	19.23342984349124	3.71551887696544	3.16691953550641
H	20.30610009374967	3.77884049350436	3.36622963501721
C	18.92928006293846	2.58102977198473	2.18043999847353
H	17.85228999563313	2.39040002313364	2.16355999437867
C	19.35188996824006	2.94280002849643	0.76293996585087
H	20.40923999677309	2.96464998711251	0.63966000531622
C	18.81574999370978	4.30981002249374	0.36278000943873
H	17.73681999997953	4.20884999619304	0.21842999757976
C	19.12621006409107	5.36443007149565	1.44018007575617
H	20.19895000410320	5.58348997555136	1.44841998779180
C	18.34933000693998	6.65546001257905	1.15672999365973
H	17.33060999468781	6.41082999807061	0.83641000240575
H	18.26339999703843	7.24252999844866	2.07683999721884
O	19.64478998494674	1.40974001156235	2.53550997799135
O	18.80300999855083	1.98201999955287	-0.13625999293487
O	19.46175000097610	4.69534999408667	-0.84581000155273
O	18.70027994038799	4.94923977213053	2.72804980613859

O	18.94381999876412	7.39039999716440	0.08776000208571
H	19.43039000586430	1.15222000746648	3.46149000980825
H	19.10754999999883	1.10187999751434	0.16093000190374
H	19.73968999987525	7.85779000105550	0.41028000077818
C	22.51997999838382	7.86374000331498	0.44025000080153
H	23.58636000243450	8.05468999612424	0.61907999668623
C	22.39085000187519	6.71213000215590	-0.56470000117991
H	21.37433999858610	6.31164999831149	-0.57650999808489
C	22.87111000069087	7.07474000076196	-1.94658000115461
H	23.96940999911381	7.07302000012651	-1.95998000111889
C	22.31139000153360	8.41665000261495	-2.37778999789283
H	21.24828999779110	8.28925000233122	-2.61260000131588
C	22.47589999825700	9.46190999797866	-1.26088000132732
H	23.54196999844072	9.66229999800889	-1.09963000061090
C	21.77893000009654	10.76918999780846	-1.61249999951132
H	20.74205000206172	10.57048999928073	-1.90878000003964
H	21.74673000088923	11.40638000030197	-0.72398000188832
O	23.24886533040123	5.64574326376338	-0.13398380502198
O	22.42852518607295	6.11872269415555	-2.91503920281864
O	22.98971000089859	8.92291999740980	-3.52276999852367
O	21.86953999743290	9.01555999743092	-0.04635999819427
O	22.48583999937344	11.48738999812757	-2.61701000014527
H	22.94220767873999	5.34704715975316	0.75589822111555
H	22.70246503073229	5.24385467146226	-2.57524677976950
H	22.71355000046616	10.86116000174444	-3.33490999932236
C	26.11994999953347	11.86512999857221	3.16893000053972
H	27.20166000099090	11.93419000004382	3.34483999896639
C	25.82213999929020	10.73483999649853	2.18163000480290
H	24.74503000159212	10.55882000602270	2.13196000257708
C	26.32664000193912	11.10873999845584	0.79157000155388
H	27.42220000022148	11.16069999851179	0.78084999914145

C	25.71790000028423	12.45503000015040	0.37545000162167
H	24.63556999838859	12.32599000027972	0.23878999810900
C	26.00105999914572	13.51812999855910	1.44992000214247
H	27.07496000166476	13.74860999889306	1.45842000092639
C	25.20906999955331	14.79783999809887	1.15020000040943
H	24.18815999816354	14.54963000006068	0.83747000244895
H	25.11627000232030	15.39762999923364	2.06125999956978
O	26.49566999711346	9.54530000262099	2.56926999731553
O	25.90435000149848	10.13179000197842	-0.16126000190407
O	26.29276999884296	12.95334000023016	-0.82779999805491
O	25.57982000236393	13.09554000219236	2.73799999760766
O	25.86536999865289	15.58912000061838	0.16586000181064
H	26.23977000322046	9.30182999913092	3.48949999808012
H	26.26828000037172	9.27184999779810	0.12591000153663
C	22.30688000185202	8.49990999367133	5.65849000349413
H	21.24551999687288	8.28272000136995	5.82868999841536
C	22.43702000682669	9.66220000185776	4.66995000227970
H	23.47525999692314	9.99797000422397	4.62246000410283
C	21.95800998988305	9.27431999290466	3.27107999726353
H	20.86353000744807	9.24017000200489	3.25126000300998
C	22.54910998524593	7.92624998366295	2.84618996950619
H	23.62280999742987	8.03991998254044	2.65822998718861
C	22.35337000496170	6.91792016904096	3.99581918322213
H	21.28750999467399	6.69818995767307	4.13094995512166
C	23.07050945596389	5.57145011415218	3.69887264844889
O	21.60565999676393	10.71705999970397	5.12858000104333
O	22.40733000219594	10.30589000891148	2.39591000222000
O	21.92667000465865	7.43926000510385	1.65557999856007
O	22.96883998585805	7.34964998435720	5.17927996240087
H	21.94150999807869	10.99753000016015	6.00744000220787
H	22.01919000022322	10.16016999375277	1.50852000014996

C	18.72707999694824	4.43236999751861	8.35973000031514
H	17.64540999980255	4.36083000195087	8.53470000137554
C	19.02503000740728	5.56449999635816	7.37387999506191
H	20.10250999707601	5.73760999735576	7.32359000198034
C	18.52409000435665	5.19349002531805	5.98447999399477
H	17.42866000332275	5.13586999460389	5.97016000160700
C	19.14031994284214	3.84880010390598	5.56906999605307
H	20.22678000144774	3.97462996850737	5.45950001027509
C	18.84933999320663	2.78410000833628	6.64008999019006
H	17.77499000054670	2.55238999939172	6.64634000127766
C	19.60649999658118	1.43949999596668	6.34167999830383
H	20.65787000150615	1.65531000200965	6.13630000381765
H	19.56469000370823	0.81881000223237	7.24640000225898
O	18.33934000114392	6.73253000084783	7.79544999896849
O	18.96650000198886	6.23097999957003	5.11540999828668
O	18.57596010196386	3.36819978181448	4.36154994155563
O	19.27623000091118	3.19836999645962	7.92136000266177
O	19.08238999979153	0.75078000286888	5.21244000062556
H	18.61429999757627	6.96159000182124	8.71289999888726
H	18.67801999969126	6.01368000248915	4.20433999796131
H	18.15918999802505	0.48762000098382	5.38506999918689
C	25.58818000231038	12.58769000065524	8.36217000251989
H	24.50991000151104	12.52162999985992	8.54426000157725
C	25.89373000227245	13.70199000015561	7.36127000114707
H	26.97790000088958	13.86237000154719	7.31790999857166
C	25.37533000116727	13.32638000180804	5.98205000039395
H	24.27991000195995	13.26883000130182	5.97365000007550
C	26.00359000094805	11.99295000049817	5.56658999998059
H	27.08825000146300	12.12484999748554	5.46345999813067
C	25.71243999803584	10.93284000225080	6.64148999875826
H	24.64188999862366	10.70944000244450	6.64813999914224

C	26.45407999678581	9.58024999728507	6.33103000225404
H	27.49154000060270	9.78959999793947	6.05461999826757
H	26.47020000001119	8.98360000169475	7.25158000116216
O	25.24459999979344	14.87008000026835	7.84695000181365
O	25.77692999827242	14.40288000014607	5.14120000014941
O	25.46753000118271	11.49851000105575	4.35828999737571
O	26.14330000177752	11.35855000076064	7.92054999983896
O	25.85472998610259	8.86964003194416	5.25518000120277
H	25.57142000239413	14.17742000233958	4.20780000194680
H	25.03370002147988	8.43017996728127	5.55296999848412
C	19.25738999758513	3.71559000197169	13.54451000111131
H	20.33536999904882	3.78280000039601	13.73081000158451
C	18.96504000062412	2.58336999866608	12.56039999870863
C	19.47022000179796	2.95813000079054	11.17255999716943
H	20.56565000192883	3.01761000049984	11.16018000262318
C	18.84514999861320	4.29868999733565	10.75849999897168
H	17.76004999750594	4.16953999766053	10.65474000103906
C	19.13531999748787	5.36256000183708	11.82841000027351
H	20.20580000170484	5.58671000015128	11.83615000002963
C	18.34993000148927	6.64921000167701	11.53609000178887
H	17.32957000088092	6.39987999973014	11.22616999832162
H	18.26985000119621	7.24687000100108	12.45040000117517
O	19.67430000173378	1.42928000065597	12.97380999813049
O	19.03980999901474	1.91275999994611	10.31068999913673
O	19.38095000251916	4.79210999879705	9.54753000241619
O	18.70719000262538	4.94298999861083	13.10871000053278
O	18.93953000195953	7.41805000180710	10.48953999992650
H	19.38011999869281	1.16101000068799	13.87340000194244
H	19.31091999780897	2.13156000111162	9.39544999736345
H	19.79890000004483	7.77352000040327	10.79156000034788
C	22.53143901534444	7.83456099850154	10.82295000970384

H	23.59490999354396	8.03634000231910	11.00214999415801
C	22.40688098696615	6.65346999597191	9.85343099115381
H	21.37217999962276	6.30593000267383	9.80961900339832
C	22.88810001106771	7.03835999783093	8.46013000860258
H	23.98287000163066	7.06353000261604	8.44137000113357
C	22.29862999601428	8.38962999508050	8.02988999821663
H	21.22561999819643	8.26468999948153	7.84414000310218
C	22.47559000438762	9.44503000250629	9.12939000152250
H	23.54195000104522	9.65439000049216	9.28017999850835
C	21.77142000179246	10.75757999795485	8.77203000101788
H	20.73224999992733	10.55920999973002	8.48934000117012
H	21.75544999986855	11.40851999821869	9.65152999818659
O	23.11391915638261	5.54848907907732	10.43281508160589
O	22.44591930270079	6.01273009644251	7.55919152424276
O	22.92174999579399	8.88047000508287	6.85705000182361
O	21.88079999357927	9.00000999803141	10.33877999692232
O	22.46007000086124	11.43968000015897	7.71635999968290
H	22.78522796174987	6.26078266381788	6.66636055953315
H	22.14384999958729	12.36409000249969	7.69099000056011
C	26.12066000146653	11.86153000036603	13.54481999827038
H	27.20221000137036	11.93311000213047	13.72135999738992
C	25.82281000090394	10.73088000207069	12.55969000156258
H	24.74520000048885	10.55774999985515	12.51120000166476
C	26.32398999890428	11.10342000000000	11.17153000165884
H	27.41912999980071	11.16325999909901	11.15937000153125
C	25.70315000048467	12.44785999822850	10.75981999984608
H	24.61726999746461	12.32050999987177	10.65201000222425
C	25.99711999963519	13.51075999939600	11.83289999995985
H	27.07205999942564	13.74061999985273	11.83836999892442
C	25.21174000019652	14.79465000147114	11.53134999767651
H	24.19115999901010	14.55407999937528	11.21002999838999

H	25.11485999906136	15.39065999984283	12.44426000036466
O	26.49761999961566	9.55841000209767	12.98932999829883
O	25.88128000232620	10.06340000193741	10.30334999891503
O	26.24310999873772	12.94737999904873	9.55001999816260
O	25.57216999995854	13.09017000186288	13.11609000113869
O	25.86934000249026	15.62436000116702	10.58464999819582
H	26.18364000045398	9.31650000178974	13.89151000185930
H	26.17821000182545	10.27382000008377	9.39398000118496
C	22.22383999832148	8.45909999891753	15.97585999856257
C	22.40805999907183	9.64893999872061	15.03086999792756
H	23.45669000096597	9.95827999722108	14.99913000172274
C	21.94669999688558	9.26594999925405	13.63160999803047
H	20.85294000173787	9.23964999837074	13.60641000225366
C	22.53997999996628	7.91097999845365	13.20422999879776
H	23.61551999950553	8.03286000050488	13.02623000191094
C	22.36087000334326	6.85008999618321	14.30277100288620
H	21.29651000042423	6.62869999861739	14.44269999769931
C	23.06799110645471	5.51746289696871	13.99899305162671
H	24.14206103540700	5.72802465394905	13.76787242020528
H	23.05342179455956	4.92893032957550	14.94054968442079
O	21.62576000109951	10.70989999910149	15.57113999834191
O	22.37839000108967	10.31406000072871	12.76355999796066
O	21.91062999542140	7.45014000186248	12.01943999758021
O	22.92465000097638	7.33292000139919	15.50444999993467
O	22.44124076039191	4.74965806987059	12.99698413041027
H	21.68646999920298	11.47128999853567	14.96269999789299
H	22.07507000154828	10.11310999873104	11.85391999815940
H	22.60916497176216	5.18451401553666	12.12395440901250
C	18.65217999968170	4.43251999834477	18.68929999751586
C	19.00102000256037	5.56901999770921	17.72952999833777
H	20.08904999965594	5.71781999838207	17.70012999745795

C	18.51443000229648	5.19685000049180	16.33899999886826
H	17.41944000197142	5.13961999871724	16.32171999759615
C	19.13619000176511	3.84687999995291	15.93549000008599
H	20.22367999996443	3.97440999792679	15.83557999904928
C	18.84339999897679	2.78068999823363	17.00614000258270
H	17.77027000220299	2.54287999924667	17.00843000107501
C	19.61157999815429	1.44123000212192	16.72077000090681
H	20.65160000143518	1.66207000243070	16.46630000053552
H	19.61201999841990	0.85412999853639	17.64823000081861
O	18.34277000131977	6.75532000158708	18.14932999779806
O	18.96212000078157	6.22998000011571	15.46888000080201
O	18.59356999903284	3.35859999825483	14.72868999918992
O	19.25943000241982	3.21812000206687	18.28401000006450
O	19.05520999769413	0.71098999790616	15.63415000177536
H	18.53363000136143	6.88083999929573	19.10059000195006
H	18.69229999836101	6.00409999964146	14.55373000156957
H	18.14190000053659	0.44334999922005	15.84717000122091
C	25.50858999868857	12.58265000054037	18.70099999975039
C	25.86082000072240	13.71004999874743	17.72955999739442
H	26.94982000080736	13.84960999914413	17.70298999918230
C	25.36857000042682	13.33357000134361	16.34137999982458
H	24.27345999857566	13.27450999894247	16.32528000022124
C	26.00181000196853	11.99580000143312	15.93517000134394
H	27.08733000044955	12.12649000225240	15.83735999770524
C	25.70805000145083	10.93638000181662	17.00640999957996
H	24.63535999968209	10.71759000227352	17.00907999987015
C	26.42991999931780	9.57505000086416	16.69945000145157
H	27.44933000014816	9.76745000178430	16.35453000046922
H	26.49432999905926	9.01855000258082	17.64276999800790
O	25.22639999748568	14.90145000088868	18.17044999994215
O	25.77983000050419	14.40858000069101	15.50287000011446

O	25.46745000032562	11.49519000143538	14.72885999742787
O	26.13141999976094	11.37547999898665	18.27753999903861
O	25.76465000268252	8.82922999683880	15.68666000234933
H	25.58642999748826	14.18221999945978	14.56820999923109
H	24.93700999966027	8.44604000336517	16.04300999768364
H	24.42291999964441	12.47615000226340	18.83228999757491
O	25.98265999958108	12.84662999797827	20.00537000118468
H	17.56731000016053	4.32562000031079	18.82683000005687
O	19.14463000251194	4.73933000091161	19.98558999744147
H	20.10054999936985	4.53987999954160	20.00990999766508
H	26.94877000209911	12.97993000134072	19.97291999750250
H	21.16255999846947	8.23140000072499	16.13268000188442
O	22.84906000169848	8.74528999899442	17.22040000263085
H	22.43204000237107	9.54983000250568	17.58851999903632
H	19.10892999903987	5.56436000037492	-1.12520999714597
H	22.96751000101436	8.24112000170187	-4.22192000035488
H	26.14579000263599	12.29888999934849	-1.53767999915130
H	26.06117000023157	15.00893999975911	-0.59863000235471
H	25.76286999798054	15.25813999839723	9.68067999993170
H	25.29410999932339	15.54710000175303	17.43850999807008
H	25.32345000209251	15.56400000087319	7.16220000192211
O	22.61861439588525	4.85513683923379	2.56145727071201
H	22.99979666642816	4.94283765002378	4.61912553420706
H	24.14721996626536	5.77985372107676	3.53096925369117
H	21.70947925699405	4.53968809251959	2.72883052617318
C	24.94525997836652	5.22419252603352	10.06221703993650
C	25.21525902621833	4.23061295530420	11.14452110151117
O	25.52238344240524	6.20118531869289	9.71581808045339
H	17.91012000228255	2.40622000107678	12.53464999760448
H	26.11229957243450	3.65060116649050	10.85250823228472
H	24.36439239356780	3.55164299410694	11.32291939832732

H	25.43787700516456	4.79197975641421	12.07257399171758
H	22.98905357413201	1.67495310337982	7.48752355668988
O	22.54778330971197	3.51470869744993	9.30040974287576
H	22.71481671415603	4.50505641255528	9.95214628110802
H	22.61076667838650	3.17722593672979	6.59524696342574
C	23.37493413784022	2.67868062871176	7.22615626278836
C	23.51924303742410	3.51007486156897	8.47325368624127
H	24.32705902718792	2.60354482331575	6.67329522238200
O	24.60100413871801	4.18034916047978	8.61672584086974

3-OAc Transition State

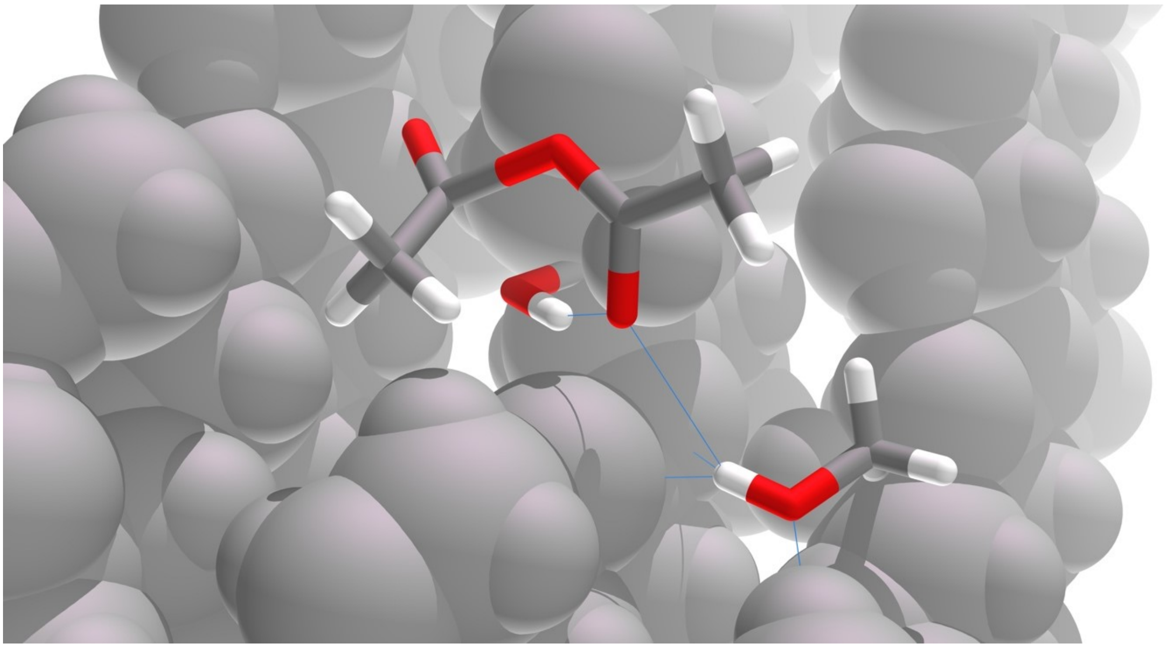

274

Coordinates from ORCA-job orca

C	19.233430	3.715518	3.166919
H	20.306100	3.778842	3.366231
C	18.929280	2.581030	2.180440
H	17.852290	2.390400	2.163560
C	19.351890	2.942800	0.762940
H	20.409240	2.964650	0.639660
C	18.815750	4.309810	0.362780

H	17.736820	4.208850	0.218430
C	19.126210	5.364430	1.440180
H	20.198950	5.583490	1.448420
C	18.349330	6.655460	1.156730
H	17.330610	6.410830	0.836410
H	18.263400	7.242530	2.076840
O	19.644790	1.409740	2.535510
O	18.803010	1.982020	-0.136260
O	19.461750	4.695350	-0.845810
O	18.700280	4.949240	2.728050
O	18.943820	7.390400	0.087760
H	19.430390	1.152220	3.461490
H	19.107550	1.101880	0.160930
H	19.739690	7.857790	0.410280
C	22.519980	7.863740	0.440250
H	23.586360	8.054690	0.619080
C	22.390850	6.712130	-0.564700
H	21.374340	6.311650	-0.576510
C	22.871110	7.074740	-1.946580
H	23.969410	7.073020	-1.959980
C	22.311390	8.416650	-2.377790
H	21.248290	8.289250	-2.612600
C	22.475900	9.461910	-1.260880
H	23.541970	9.662300	-1.099630
C	21.778930	10.769190	-1.612500
H	20.742050	10.570490	-1.908780
H	21.746730	11.406380	-0.723980
O	23.246225	5.643865	-0.135558
O	22.430391	6.116981	-2.914955
O	22.987140	8.926050	-3.524430
O	21.869540	9.015560	-0.046360

O	22.484640	11.486930	-2.618750
H	22.932421	5.341312	0.752083
H	22.695736	5.243014	-2.565779
H	22.716940	10.857070	-3.332130
C	26.119950	11.865130	3.168930
H	27.201660	11.934190	3.344840
C	25.822140	10.734840	2.181630
H	24.745030	10.558820	2.131960
C	26.326640	11.108740	0.791570
H	27.422200	11.160700	0.780850
C	25.717900	12.455030	0.375450
H	24.635570	12.325990	0.238790
C	26.001060	13.518130	1.449920
H	27.074960	13.748610	1.458420
C	25.209070	14.797840	1.150200
H	24.188160	14.549630	0.837470
H	25.116270	15.397630	2.061260
O	26.495670	9.545300	2.569270
O	25.904350	10.131790	-0.161260
O	26.292770	12.953340	-0.827800
O	25.579820	13.095540	2.738000
O	25.865370	15.589120	0.165860
H	26.239770	9.301830	3.489500
H	26.268280	9.271850	0.125910
C	22.306880	8.499910	5.658490
H	21.245520	8.282720	5.828691
C	22.437020	9.662200	4.669950
H	23.475260	9.997970	4.622460
C	21.958010	9.274320	3.271080
H	20.863530	9.240170	3.251260
C	22.549110	7.926250	2.846190

H	23.622810	8.039920	2.658230
C	22.353370	6.917920	3.995820
H	21.287510	6.698190	4.130950
C	23.101134	5.606065	3.645474
O	21.605660	10.717060	5.128580
O	22.407330	10.305890	2.395910
O	21.926670	7.439260	1.655580
O	22.968841	7.349650	5.179280
H	21.941510	10.997530	6.007440
H	22.019190	10.160170	1.508520
C	18.727080	4.432370	8.359730
H	17.645410	4.360830	8.534700
C	19.025030	5.564500	7.373880
H	20.102510	5.737610	7.323590
C	18.524090	5.193490	5.984480
H	17.428660	5.135870	5.970160
C	19.140320	3.848803	5.569071
H	20.226781	3.974629	5.459500
C	18.849340	2.784100	6.640090
H	17.774990	2.552390	6.646340
C	19.606500	1.439500	6.341680
H	20.657870	1.655310	6.136300
H	19.564690	0.818810	7.246400
O	18.339340	6.732530	7.795450
O	18.966500	6.230980	5.115410
O	18.575960	3.368200	4.361550
O	19.276230	3.198370	7.921360
O	19.082390	0.750780	5.212440
H	18.614300	6.961590	8.712900
H	18.678020	6.013680	4.204340
H	18.159190	0.487620	5.385070

C	25.588180	12.587689	8.362170
H	24.509910	12.521631	8.544260
C	25.893730	13.701990	7.361270
H	26.977900	13.862370	7.317910
C	25.375330	13.326380	5.982050
H	24.279910	13.268830	5.973650
C	26.003590	11.992950	5.566590
H	27.088250	12.124850	5.463460
C	25.712440	10.932840	6.641490
H	24.641890	10.709440	6.648140
C	26.454080	9.580250	6.331029
H	27.491540	9.789600	6.054620
H	26.470200	8.983600	7.251580
O	25.244600	14.870080	7.846950
O	25.776930	14.402880	5.141200
O	25.467530	11.498510	4.358290
O	26.143300	11.358550	7.920550
O	25.854732	8.869640	5.255186
H	25.571420	14.177420	4.207800
H	25.033697	8.430180	5.552964
C	19.257390	3.715590	13.544510
H	20.335370	3.782800	13.730810
C	18.965040	2.583370	12.560400
C	19.470220	2.958130	11.172560
H	20.565650	3.017610	11.160180
C	18.845150	4.298690	10.758500
H	17.760050	4.169540	10.654740
C	19.135320	5.362560	11.828420
H	20.205800	5.586710	11.836150
C	18.349930	6.649210	11.536090
H	17.329570	6.399880	11.226170

H	18.269850	7.246870	12.450400
O	19.674300	1.429280	12.973810
O	19.039810	1.912760	10.310690
O	19.380950	4.792110	9.547530
O	18.707190	4.942990	13.108710
O	18.939530	7.418050	10.489540
H	19.380120	1.161010	13.873400
H	19.310920	2.131560	9.395450
H	19.798900	7.773520	10.791560
C	22.531440	7.834560	10.822950
H	23.594910	8.036340	11.002150
C	22.424941	6.604940	9.872490
H	21.372180	6.305930	9.809620
C	22.894499	6.980801	8.441890
H	23.982870	7.063529	8.441370
C	22.298630	8.389630	8.029890
H	21.225620	8.264690	7.844140
C	22.475590	9.445030	9.129390
H	23.541950	9.654390	9.280180
C	21.771420	10.757580	8.772030
H	20.732250	10.559210	8.489340
H	21.755450	11.408520	9.651530
O	23.281250	5.648255	10.437648
O	22.606920	5.960052	7.480997
O	22.921750	8.880470	6.857050
O	21.880800	9.000010	10.338780
O	22.460070	11.439680	7.716360
H	22.203985	12.380726	7.760782
C	26.120660	11.861530	13.544820
H	27.202210	11.933110	13.721360
C	25.822810	10.730880	12.559690

H	24.745200	10.557750	12.511200
C	26.323990	11.103420	11.171530
H	27.419130	11.163260	11.159370
C	25.703150	12.447860	10.759820
H	24.617270	12.320510	10.652010
C	25.997120	13.510760	11.832900
H	27.072060	13.740620	11.838370
C	25.211740	14.794650	11.531350
H	24.191160	14.554080	11.210030
H	25.114860	15.390660	12.444260
O	26.497620	9.558410	12.989330
O	25.881280	10.063400	10.303350
O	26.243110	12.947380	9.550020
O	25.572170	13.090170	13.116090
O	25.869340	15.624360	10.584650
H	26.183640	9.316500	13.891510
H	26.178210	10.273820	9.393980
C	22.223840	8.459100	15.975860
C	22.408060	9.648940	15.030870
H	23.456690	9.958280	14.999130
C	21.946700	9.265950	13.631610
H	20.852940	9.239650	13.606410
C	22.539980	7.910980	13.204230
H	23.615520	8.032860	13.026230
C	22.360870	6.850090	14.302770
H	21.296510	6.628700	14.442700
C	23.092688	5.535839	13.997929
H	24.137682	5.754153	13.679722
H	23.135212	4.965562	14.951727
O	21.641114	10.721016	15.578327
O	22.378390	10.314060	12.763560

O	21.910630	7.450140	12.019440
O	22.924650	7.332920	15.504450
O	22.392193	4.794281	12.989623
H	21.826534	11.495470	15.013670
H	22.075070	10.113110	11.853920
H	22.705741	3.870303	13.016303
C	18.676013	4.437163	18.712900
C	19.001020	5.569020	17.729530
H	20.089050	5.717820	17.700130
C	18.514430	5.196850	16.339000
H	17.419440	5.139620	16.321720
C	19.136190	3.846880	15.935490
H	20.223680	3.974410	15.835580
C	18.843400	2.780690	17.006140
H	17.770270	2.542880	17.008430
C	19.611580	1.441230	16.720770
H	20.651600	1.662070	16.466300
H	19.612020	0.854130	17.648230
O	18.342770	6.755320	18.149330
O	18.962120	6.229980	15.468880
O	18.593570	3.358600	14.728690
O	19.259430	3.218120	18.284010
O	19.055210	0.710990	15.634150
H	18.533630	6.880840	19.100590
H	18.692300	6.004100	14.553730
H	18.141900	0.443350	15.847170
C	25.508590	12.582650	18.701000
C	25.860820	13.710050	17.729560
H	26.949820	13.849610	17.702990
C	25.368570	13.333570	16.341380
H	24.273460	13.274510	16.325280

C	26.001810	11.995800	15.935170
H	27.087330	12.126490	15.837360
C	25.708050	10.936380	17.006410
H	24.635360	10.717590	17.009080
C	26.429920	9.575050	16.699450
H	27.449330	9.767450	16.354530
H	26.494330	9.018550	17.642770
O	25.226400	14.901450	18.170450
O	25.779830	14.408580	15.502870
O	25.467450	11.495190	14.728860
O	26.131420	11.375480	18.277540
O	25.764650	8.829230	15.686660
H	25.586430	14.182220	14.568210
H	24.937010	8.446040	16.043010
H	24.422920	12.476150	18.832290
O	25.982660	12.846630	20.005370
H	17.567310	4.325620	18.826830
O	19.144630	4.739330	19.985590
H	20.100550	4.539880	20.009910
H	26.948770	12.979930	19.972920
H	21.162560	8.231400	16.132680
O	22.849060	8.745290	17.220400
H	22.432040	9.549830	17.588520
H	19.108930	5.564360	-1.125210
H	22.980580	8.237230	-4.216660
H	26.145790	12.298890	-1.537680
H	26.061170	15.008940	-0.598630
H	25.761690	15.259160	9.680350
H	25.294110	15.547100	17.438510
H	25.323450	15.564000	7.162200
O	22.613644	4.874209	2.525769

H	23.111131	4.974890	4.551202
H	24.155711	5.861773	3.418652
H	21.755638	4.471505	2.758554
H	17.859641	2.437548	12.514046
H	22.850389	5.317054	11.263951
C	22.732016	4.207671	7.952921
O	22.137254	3.860711	8.910966
C	22.810803	3.623671	6.590817
H	22.961682	2.533212	6.704688
H	23.627890	4.045131	5.988641
H	21.847833	3.799690	6.078483
O	24.900141	5.849891	6.703924
H	27.385709	5.306534	6.881983
H	23.629773	5.947853	6.909769
H	27.106927	5.731697	8.603834
C	26.856679	5.003490	7.803904
C	25.361975	5.037149	7.588420
H	27.181790	4.003397	8.143503
O	24.635012	4.286886	8.311933

8. References

Becke A D (1988) Density-functional exchange-energy approximation with correct asymptotic behaviour. *Phys. Rev. A, General Physics,* 38: 3098. DOI: 10.1103/physreva.38.3098

Rico del Cerro D, Koso T V, Kakko T, King A W T, Kilpeläinen I. (2020) Crystallinity reduction and enhancement in the chemical reactivity of cellulose by non-dissolving pre-treatment with tetrabutylphosphonium acetate. Cellulose, 27, 5545–5562. DOI: 10.1007/s10570-020-03044-6

Eichkorn K, Treutler O, Öhm H, Häser M, Ahlrichs R (1995) Auxiliary basis sets to approximate Coulomb potentials. *Chem. Phys. Lett*. 242: 652. DOI: 10.1016/0009-2614(95)00838-U

Eichkorn K, Weigend F, Treutler O, Ahlrichs R (1997), Auxiliary basis sets for main row atoms and transition metals and their use to approximate Coulomb potentials. Theo. Chem. Acc. 97: 119. DOI: 10.1007/s002140050244

Fernandes AN, Thomas LH, Altaner CM, Callow P, Forsythe V T, Apperley D C, Kennedy C J, Jarvis M C. (2011) Nanostructure of cellulose microfibrils in spruce wood. Proc Natl Acad Sci 108:E1195–E1203. DOI: 10.1073/pnas.1108942108

Gomes T C F, Munir S S (2012) Cellulose-Builder: A toolkit for building crystalline structures of cellulose. *J. Comput. Chem.*, 33: 1338–1346. DOI: 10.1002/jcc.22959; Web Interface: http://cces-sw.iqm.unicamp.br/cces/admin/cellulose/view

Grimme S, Antony J, Ehrlich S, Krieg H (2010) A consistent and accurate ab initio parametrization of density functional dispersion correction (DFT-D) for the 94 elements H-Pu *J. Chem. Phys.* **132**: 154104. DOI:10.1063/1.3382344

Grimme S, Ehrlich S Goerigk L (2011) Effect of the damping function in dispersion corrected density functional theory. *J. Comput. Chem*. 32: 1456. DOI:10.1002/jcc.21759

Hanwell M D, Curtis D E, Lonie D C, Vandermeersch T, Zurek E, Hutchison G R (2012) Avogadro: An Advanced Semantic Chemical Editor, Visualization, and Analysis Platform. *J. Cheminformatics* 4: 17. DOI: 10.1186/1758-2946-4-17

Halgren T A (1996) Merck molecular force field. I. Basis, form, scope, parameterization, and performance of MMFF94. *J. Comput. Chem*. 17: 490. DOI: 10.1002/(SICI)1096-987X(199604)17:5/6<490::AID-JCC1>3.0.CO;2-P

Kay LE, Keifer P, Saarinen T (1992) Pure Absorption Gradient Enhanced Heteronuclear Single Quantum Correlation Spectroscopy with Improved Sensitivity J. Am. Chem. Soc. 114: 10663-10665. DOI: 10.1021/ja00052a088

King AWT, Mäkelä V, Kedzior SA, Laaksonen T, Partl GJ, Heikkinen S, Koskela H, Heikkinen HA, Holding AJ, Cranston ED, Kilpeläinen I (2018) Liquid-state NMR analysis of nanocelluloses. Biomacromolecules 19: 2708-2720 doi: 10.1021/acs.biomac.8b00295

Koso T, Rico del Cerro D, Heikkinen S, et al (2020) 2D Assignment and quantitative analysis of cellulose and oxidized celluloses using solution-state NMR spectroscopy. Cellulose 27:7929–7953. doi: 10.1007/s10570-020-03317-0

Neese F (2018) Software update: the ORCA program system, version 4.0. *WIREs Comput. Mol. Sci*. 8:e1327. DOI: 10.1002/wcms.1327

Oehme D P, Downton M T, Doblin M S, Wagner J, Gidley M J, Bacic A (2015) Unique Aspects of the Structure and Dynamics of Elementary Iβ Cellulose Microfibrils Revealed by Computational Simulations. Plant Phys. 168, 1, 3-17. DOI: 10.1104/pp.114.254664

Paajanen A, Ceccherini S, Maloney T, Ketoja J A (2019) Chirality and bound water in the hierarchical cellulose structure. Cellulose, 26, 5877–5892. DOI: 10.1007/s10570-019-02525-7

Palmer III AG, Cavanagh J, Wright PE, Rance M (1991) Sensitivity improvement in proton-detected two-dimensional heteronuclear correlation NMR spectroscopy J. Magn. Reson. 93: 151-170. DOI: 10.1016/0022-2364(91)90036-S

Perdew J P (1986) Density-functional Approximation for the Correlation Energy of the Inhomogeneous Electron Gas. *Phys. Rev. B, Condensed Matter*, 33: 8822. DOI: 10.1103/physrevb.33.8822

Schäfer A, Horn H, Ahlrichs R (1992) Fully optimized contracted Gaussian-basis sets for atoms Li to Kr. *J. Chem. Phys*. 97: 2571. DOI: 10.1063/1.463096

Schleucher J, Schwendinger M, Sattler M, Schmidt P, Schedletzky O, Glaser SJ, Sorensen OW, Griesinger C (1994) A general enhancement scheme in heteronuclear multidimensional NMR employing pulsed field gradients J. Biomol. NMR 4: 301-306. DOI: 10.1007/BF00175254

Sixta H, Michud A, Hauru L, et al (2015) Ioncell-F: A High-strength regenerated cellulose fibre. Nord Pulp Pap Res J 30:043–057. doi: 10.3183/NPPRJ-2015-30-01-p043-057

Tao J, Perdew J P, Staroverov V N, Scuseria G E (2003) Climbing the Density Functional Ladder: Nonempirical Meta-Generalized Gradient Approximation Designed for Molecules and Solids. *Phys. Rev. Lett*., 91: 146401. DOI: 10.1103/PhysRevLett.91.146401

Wang T, Hong M (2015) Solid-state NMR investigations of cellulose structure and interactions with matrix polysaccharides in plant primary cell walls. J. Exp. Botany 67, 2, 503–514. DOI: 10.1093/jxb/erv416

Weigend F, Ahlrichs R (2005) Balanced basis sets of split valence, triple zeta valence and quadruple zeta valence quality for H to Rn: Design and assessment of accuracy. *Phys. Chem. Chem. Phys*. 7: 3297. DOI: 10.1039/B508541A

Willker W, Leibfritz D, Kerssebaum R, Bermel W (1993) Gradient selection in inverse heteronuclear correlation spectroscopy. Magn. Reson. Chem. 31: 287–292

Wojdyr M (2010) *Fityk*: a general-purpose peak fitting program. J. Appl. Cryst. 43: 1126-1128 https://doi.org/10.1107/S0021889810030499

Wu DH, Chen AD, Johnson CS (1995) An improved diffusion-ordered spectroscopy experiment incorporating bipolar-gradient pulses. J. Magn. Reson. A 115: 260–264 https://doi.org/10.1006/jmra.1995.1176

Zheng J, Xu X, Truhlar D G (2010) Minimally augmented Karlsruhe basis sets. *Theor. Chem. Acc*. 128: 295. DOI: 10.1007/s00214-010-0846-z
